# A New Strategy to Functionalize Exosomes via Enzymatic Engineering of Surface Glycans and its Application to Profile Exosomal Glycans and Endocytosis

**DOI:** 10.1002/advs.202415942

**Published:** 2025-03-19

**Authors:** Sayan Kundu, Jiatong Guo, Md. Shamiul Islam, Rajendra Rohokale, Mohit Jaiswal, Zhongwu Guo

**Affiliations:** ^1^ Department of Chemistry University of Florida Gainesville FL 32611 USA; ^2^ UF Health Cancer Center University of Florida Gainesville FL 32611 USA

**Keywords:** exosomes, extracellular vesicles, carbohydrates, sialic acid, glycoengineering

## Abstract

Exosomes are membrane‐enclosed nanoparticles secreted by cells to mediate intercellular communication. Hence, functionalized exosomes are powerful tools in biology and medicine, and efficient methods to functionalize exosomes are highly desired. In this work, a novel approach is developed to modify and functionalize exosomes based on enzymatic engineering of their surface glycans. It employs a sialyltransferase and an azide‐modified sialyl donor to enzymatically install azido‐sialic acids onto exosomal glycans. The azide tags serve as universal molecular handles to attach various probes, e.g., biotin, protein, fluorophore, etc., by simple and biocompatible click chemistry. This approach is easy and effective, and the modified exosomes are readily retrieved from the plate, enabling the production of functional exosomes in practical scales for various studies and applications. The functionalized exosomes obtained are employed to profile exosomal glycans, disclosing the diverse glycosylation patterns of exosomes of different origins. They also facilitated comprehensive investigations on the cellular uptake of exosomes to disclose macropinocytosis as the main and general uptake route, while other endocytosis pathways are also partially involved in specific exosomes. Additionally, the new exosome functionalization approach has been demonstrated to be widely applicable to exosomes of different origins.

## Graphical abstract

Enzymatic introduction of azido‐sialic acid residues to the glycans on exosomes enables a new and efficient approach for exosomal functionalization with various probes, including biotin and fluorophores, by biocompatible click chemistry, thereby facilitating detailed investigations of exosomes, such as their glycan profiles, cellular uptake, etc., and other applications.

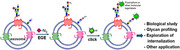

## 1 Introduction

Exosomes, one of the major extracellular vesicles (EVs) secreted by cells, are nanoparticles with a dimension of 50–150 nm that are generally observed in both cell cultures and body fluids.^[^ ^1^ ^]^ They carry many of the molecules in the donor cell, including proteins, glycans, lipids, or nucleic acids, to deliver them to recipient cells.^[^ ^2^ ^]^ Thus, EVs have the notable capability to transmit materials and information among cells.^[^ ^3^ ^]^ The size, composition, and payloads of a specific EV are primarily governed by factors like the donor cell, cell culture condition, and species of cell origin, thus they are biocompatible with the donor.^[^ ^4^ ^]^ Given the pivotal roles of EVs in mediating cellular signaling, studies on EVs hold great promise in deciphering cell interaction and other biological events. Moreover, due to their unique properties, e.g., small size, superb stability and biocompatibility, and unusual capability to bypass the endosomal pathway and directly transfer into cytoplasm,^[^ ^2^, ^3^ ^]^ EVs, especially exosomes, emerge as useful vehicles for medical applications.^[^ ^5^ ^]^ For example, exosomes stand out as one of the favorable means for targeted drug delivery and cancer immunotherapy.^[^ ^6^ ^]^

To use exosomes for various applications, they must be properly functionalized. To this end, many techniques have been explored for exosome modification in the past decade‒a subject of multiple excellent reviews.^[^ ^5^, ^7^ ^]^ Of them, an extensively studied method is genetic engineering, which incorporates a gene in the donor cell for expressing a specific surface protein/peptide to be inherited by exosomes.^[^ ^8^ ^]^ Although it is a robust method to express proteins, it has inherent problems for exosome functionalization. Particularly, challenges arise concerning the intricacy of the processes related to cell transfection/engineering and protein transfer to exosomes. Another approach is based upon metabolic engineering, involving the introduction of a tag to proteins or glycans on donor cells via modulating their synthetic pathways, then facilitating subsequent modification and functionalization of exosomes by the introduced molecular tags.^[^ ^9^ ^]^ Clearly, both approaches are cell‐based, thus they, as well as the resulting exosomes, have specific or limited application scopes, and the transfer of molecular tags from cells to exosomes is difficult to control. The other extensively exploited methods are physical and chemical/biochemical modifications,^[^ ^10^ ^]^ e.g., hybridizing exosome by liposome carrying special functional groups or attaching specific ligands/antibodies to exosome via a chemical or biochemical reaction to facilitate further modification. Although these approaches are cell‐independent and can be used to directly functionalize isolated exosomes, controlling the loading remains a challenge. More significantly, incorporating exogenous lipids by liposomes may affect the stability and other properties of exosomes.^[^ ^7a^ ^]^ Thus, current methods to engineer exosomes have inherent drawbacks or limitations, and more feasible and efficient methods are desired to meet the diverse and growing demand for specifically modified exosomes. To this end, we have developed herein a new approach for exosomal functionalization based upon enzymatic engineering of glycans on the exosome surface.

## 2 Results and Discussion

### 2.1 Enzymatic Glycoengineering of Exosomes

#### 2.1.1 Research Design

Like cells, exosomes also have their surface covered by glycans,^[^ ^11^ ^]^ whose signature is governed by the donor cells.^[^ ^11^, ^12^ ^]^ In literature, many techniques have been developed to edit cell surface glycans for glycobiology.^[^ ^13^ ^]^ Some techniques may be utilized to modify exosomes, thus exosomal glycans hold great promise as molecular handles for exosome engineering. For example, genome editing has been used to engineer EV surface glycans for functional studies,^[^ ^13g^ ^]^ and metabolic glycoengineering (MGE) has been used to prepare exosomes with modified glycans for further functionalization.^[^ ^9^ ^]^ However, as mentioned earlier, the MGE‐based approach must start with donor cells, affecting its efficiency, feasibility, and application scope.

**Figure 1:**
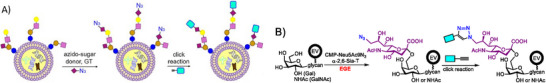
(A) General design for EGE‐based exosome engineering and labeling via enzymatic introduction of an azido‐sugar to the glycans on exosomes and then attaching molecular tags to the azido‐sugar through a biocompatible click reaction, and (B) EGE and labeling of sialoglycans on exosomes using α‐2,6‐Sia‐T and CMP‐Neu5Ac9N_3_ to attach Neu5Ac9N_3_ to the 6‐O‐positions of glycan terminal Gal/GalNAc residues.

In this study, we plan to use enzymatic glycoengineering (EGE)^[^ ^14^ ^]^ to modify glycans on exosomes for their functionalization. A useful feature and advantage of EGE over MGE is that it can be cell‐free. Therefore, we may directly utilize EGE to modify exosomes, such that the approach can be widely appliable to all exosomes or EVs. As generally outlined in **Figure** **1**A, we may employ an enzymatic protocol, e.g., glycosyltransferase (GT) combining with a modified glycosyl donor, to install unnatural sugars onto exosomal glycans. Then, the functional groups put on sugar residues can serve as handles to attach molecular tags for exosome modification using specific conjugation methods. For example, the sugar residue and tag can be modified with azide and alkyne, respectively, to enable widely adopted, biocompatible click chemistry.^[^ ^15^ ^]^ To this end, we are especially interested in exosome sialylation as many bacterial sialyltransferases (Sia‐Ts) are promiscuous and accept unnatural sialyl donors, e.g., cytidine monophosphate‐9‐azido‐neuraminic acid (CMP‐Neu5Ac9N_3_),^[^ ^16^ ^]^ to transfer them to glycans in vitro or on cells and have been commonly used to synthesize complex oligosaccharides.^[^ ^17^ ^]^ Thus, Sia‐Ts may be utilized to link modified sialic acids to galactose (Gal) or *N*‐acetylgalactosamine (GalNAc) units of glycans to enable further modification (Figure 1B), which has been recently verified on a microchip platform.^[^ ^18^ ^]^

#### 2.1.2 Optimization of Protocols to Work with Exosomes On‐Plate

Commercial exosomes of human serum (HS) and other origins (all accompanied by analysis certificates) were used for this research, whose mean size was ca. 90 nm with a dispersion of 50–150 nm, verified by our dynamic light scattering (DLS) studies. To enable exosomal engineering and scalable production, we were initially focused on establishing a practical, streamlined protocol to attach exosomes to polystyrene plates and release them from plates after modification. Although protein biomarkers on the exosome surface may serve this aim,^[^ ^19^ ^]^ they did not exhibit sufficient affinity (Figure S1, Supporting Information). Hence, we turned our attention to Annexin‐V (A5), which can interact with phosphatidylserine (PS) in the membrane bilayer.^[^ ^20^ ^]^ We have verified that A5 can immobilize exosomes onto the plate effectively. Using A5 to immobilize exosomes may have additional advantages. For example, it is not glycosylated, which minimizes potential interference with EGE,^[^ ^18^ ^]^ and A5‒PS interaction is Ca^2+^‐dependent and reversible,^[^ ^21^ ^]^ which enables exosome recovery from the plate for downstream applications.

Specifically, we evaluated first A5 attachment to the plate. In this study, we incubated the plate with various concentrations (2.5, 5.0, and 10 µg mL^−1^) of A5 and then quantified A5 adhesion employing biotinylated anti‐A5 antibody (Ab) and alkaline phosphatase (AP)‐streptavidin (Strep) conjugate by colorimetric measurement of the AP‐catalyzed *para*‐nitrophenyl phosphate (PNPP) hydrolysis. Our results (Figure S2, Supporting Information) disclose a significant increase in the optical density (OD) of A5‐coated plate as compared to negative controls (NCs), suggesting A5 attachment to the plate. Moreover, the OD values for all three concentrations of A5 were similar, hence we chose to use 5.0 µg mL^−1^ A5 in this research.

Next, we assessed A5‐mediated exosome attachment to the plate. In this experiment, plates were coated with A5 (5 µg mL^−1^), followed by incubation with exosome (5 and 12.5 µg mL^−1^). Moreover, some of the plates were treated with ethylene‐diamine tetraacetate acid (EDTA) (10.0 mm) to release the exosome. On‐plate exosomes were assayed with a cocktail of biotinylated anti‐CD63/CD81/CD9 Abs (Table S2, Supporting Information, entries 3a–d), because CD63, CD81, and CD9 are common biomarkers of exosomes.^[^ ^5^, ^22^ ^]^ However, the OD value of exosome‐treated wells was not significantly different from that of the NC or EDTA‐treated wells (Figure S3, Supporting Information), suggesting either no exosome attachment or a low sensitivity of the detection method using anti‐CD63/CD81/CD9 Abs.

To solve the puzzle, we studied the supernatant in EDTA‐treated wells with DLS. Our results (Figure S4, Supporting Information) clearly show the presence of exosomes (an average diameter: ≈120 nm), thereby validating exosome attachment to the plate. To further confirm, we used flow cytometry (FACS) to analyze exosomes in the EDTA supernatant. After successive treatments of A5‐coated plates with exosomes and EDTA, the supernatants from multiple wells were combined and condensed using a protein concentrator (3 kDa cutoff limit) to remove single molecules but retain exosomes. The concentrate was treated with aldehyde‐sulfate latex beads to load exosomes. Exosome detection was achieved via incubating the beads with a cocktail of A488‐labeled anti‐CD63/CD81/CD9 Abs, followed by fluorescent analysis. If exosomes were present in the supernatant, they should decorate the beads to show increased fluorescence. Our results (Figure S5, Supporting Information) prove that the beads incubated with EDTA supernatants of exosome‐treated wells show evidently stronger fluorescence than NCs or native beads, which further verifies the presence of exosomes in the EDTA supernatant. These results collectively prove A5‐mediated attachment of exosomes to plates, thus A5 was used in this research.

#### 2.1.3 Optimization of Protocols for Exosome EGE and Labeling

**Figure 2:**
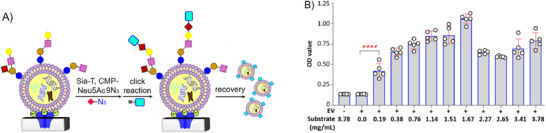
(A) Schematic presentation of the protocol for A5‐mediated exosome adhesion to plates for Sia‐T‐based EGE and labeling of exosomes and release of the functionalized exosomes from plates. (B) Substrate concentration (CMP‐Neu5Ac9N_3_, 0.19–3.78 mg mL^−1^) influence on the efficiency of exosome EGE with fixed concentrations of A5 (5.0 µg mL^−1^), HS exosome (5.0 µg mL^−1^), and Pd‐2,6‐ST (20 µg mL^−1^). Data are shown as the average of five parallel experiments ± standard deviation (SD). Level of statistical significance: ****p < 0.0001.

For exosome EGE and labeling, we are especially interested in using Sial‐Ts to install modified sialic acids, due to the abundance and promiscuity of such enzymes. Our tool enzyme selected was Pd‐2,6‐ST,^[^ ^14^, ^23^ ^]^ a bacterial Sial‐T that accepts artificial sialyl donors (including CMP‐Neu5Ac9N_3_) for α‐2,6‐sialylation (**Figure** **2**A).^[^ ^24^ ^]^ For exosome glycoengineering, after exosome attachment to A5‐coated plates under above‐mentioned conditions, a mixture of Pd‐2,6‐ST and CMP‐Neu5Ac9N_3_ was added to allow for enzymatic transfer of Neu5Ac9N_3_ to exosomal glycans. Thereafter, the plate was washed, and the azide‐tagged exosomes were labeled on‐plate with various molecular probes. In current study, the azido‐exosomes were first labeled with biotin via strain‐promoted azide‐alkyne cycloaddition (SPAAC) with dibenzocyclooctyne‐modified biotin (DBCO‐biotin). The resulting biotinylated exosomes are then conveniently and efficiently functionalized with other probes, leveraging the strong, specific, and robust binding between biotin and avidin or Strep. This approach can be widely applicable for introducing various probes, since Strep‐conjugated biomolecules are easily prepared or commercially available.

To optimize the exosome EGE conditions, we studied the impacts of enzyme and substrate concentrations on the EGE efficiency using appropriate NCs. We varied concentrations of the enzyme or substrate while keeping A5 and exosome concentrations and other experimental parameters unchanged through the process. Our results (Figure 2B) indicate that the OD values of exosomes treated with 0.19–3.78 mg mL^−1^ CMP‐Neu5Ac9N_3_ were significantly higher than that of NCs, proving the successful EGE. Moreover, within the range of 0.19–1.67 mg mL^−1^, the OD value increases in a concentration‐dependent manner, while further increases in the substrate concentration led to a small decrease in the OD value, likely due to substrate or product inhibition of Pd‐2,6‐ST. To study the impact of enzyme concentrations on exosome EGE, we kept the substrate concentration at 1.67 mg mL^−1^. The results (Figure S6, Supporting Information) show the effective labeling of exosomes, proved by higher OD values than those of NCs. All tested enzyme concentrations (0.84–33 µg mL^−1^) exhibit a similar OD value, indicating the high catalytic efficiency of Pd‐2,6‐ST. Therefore, 3.3 µg mL^−1^ of Pd‐2,6‐ST was applied to other studies in this research.

We also designed experiments to optimize other parameters and found that the OD values of Pd‐2,6‐ST/CMP‐Neu5Ac9N_3_‐treated exosomes were significantly higher than those of NCs at all tested temperatures of 4, 25, and 37 °C, with the highest OD observed at 37 °C (Figure S7, Supporting Information). Additionally, heat‐inactivated enzyme did not result in any OD value increase (or exosome EGE), which further excluded the possibility of non‐enzymatic modification. We also observed an impact of the enzyme/substrate incubation time on EGE efficiency (Figure S8, Supporting Information). While significant exosome EGE was observed upon only 15 min of incubation, the OD value reached the maximum in ≈1 h. Extending the incubation time beyond this point did not help further, thus 1 h was selected as the standard enzymatic reaction time in this research.

We finally explored the DBCO‐biotin concentration required for complete functionalization of azido‐exosome. The results (Figure S9, Supporting Information) show that while significant labeling of exosomes occurs with 25 µm DBCO‐biotin, the OD value still grows with the increased DBCO‐biotin concentration to reach a plateau at ≈100 µm. Thus, we used 100 µm DBCO‐biotin for the functionalization of azide‐tagged exosomes in this research.

#### 2.1.4 Recovery of the Functionalized Exosomes

**Figure 3:**
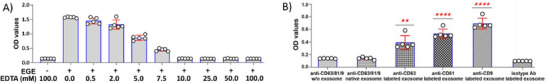
(A) Changes in the OD value after treating the plate containing A5‐anchored exosomes with different concentrations of EDTA to deplete Ca^2+^ ions. (B) Reattachment of EDTA‐released biotinylated exosomes to anti‐CD63, CD81 CD9, and isotype Ab‐coated plates using plates treated without exosome or with native exosome as NCs. The exosomes were assessed by the AP‐Strep/PNPP colorimetric method. Data are shown as the average of five parallel experiments ± SD. Levels of statistical significance: **p < 0.01 and ****p < 0.0001 as compared to NCs.

A facile and efficient method to recover functionalized exosomes from the plate is key for their production and downstream applications. To address this issue, we utilized the reversible nature of Ca^2+^‐mediated A5‒PS interaction.^[^ ^20^, ^21^ ^]^ Depleting Ca^2+^ with EDTA is expected to reverse A5‒PS binding and release exosomes from plates. To verify, after exosomes were attached to A5‐coated plates, EGE‐modified and biotinylated, EDTA was added, which was followed by extensive washing. The exosomes remaining on the plates were applied to AP‐Strep and PNPP treatment and assay. We find that exosome release is proportional to EDTA concentration, reflected by the reduced on‐plate OD value (**Figure** **3**A). Although 5.0–7.5 mm of EDTA can release exosome, only at 10 mm or above, is exosome release complete. This was also verified by fluorescence analysis. After EGE‐modified exosomes were biotinylated and treated with A488‐Strep on‐plate to attach a fluorescent label, the plate with or without EDTA treatment was subjected to fluorescence study. Our results (Figure S10, Supporting Information) show a drastic decrease in the fluorescence intensity upon treatment with 5.0 and 7.5 mm EDTA and complete disappearance of fluorescence at ca. 10 mm. Thus, 10 mm EDTA was used to release exosomes from plates in this research.

We have further characterized the biotinylated and A488‐labeled exosomes in the EDTA supernatant by DLS and found that their hydrodynamic diameters are ≈120 and 150–175 nm (Figure S11, Supporting Information), respectively. The slightly increased size of the A488‐exosomes is probably caused by the presence of Strep‐A488 molecules on the exosomal surface. Additionally, we analyzed the biomarkers on azide‐functionalized exosomes with A488‐labeled Abs and FACS. The results (Figure S12, Supporting Information) confirm the presence of CD81, CD63, CD9, HSP70, and Syntenin, indicating that these biomarkers are not influenced by EGE. The results also support EDTA‐mediated release and recovery of exosomes from the plate.

To further verify exosome EGE and EDTA‐mediated release, we examined the released biotinylated exosomes by enzyme‐linked immunosorbent assay (ELISA). Thus, EDTA supernatants were collected, concentrated, and incubated in plates coated with anti‐CD63, CD9, and CD81 Abs. The presence of biotin on engineered exosomes was established with the AP‐Strep/PNPP colorimetric method. Our results (Figure 3B) show an increased OD value for Pd‐2,6‐ST/CMP‐Neu5Ac9N_3_‐treated exosomes, as compared to NCs without exosome, with native exosome, and using isotype Ab to coat plates. The results prove the presence of exosomes in the EDTA supernatant and the glycoengineering and functionalization of these exosomes to contain biotin.

#### 2.1.5 Validation of Enzymatic Engineering of Glycans on Exosomes

To validate that treating exosomes with Pd‐2,6‐ST and CMP‐Neu5Ac9N_3_ followed by DBCO‐biotin did edit and label glycans, we incubated the labeled exosomes on‐plate using peptide *N*‐glycosidase (PNGase) F that selectively cleaves *N*‐glycans in glycoproteins.^[^ ^25^ ^]^ We anticipated that PNGase F treatment would result in a decrease in the OD value, but this was not observed initially (Figure S13, Supporting Information). While we cannot completely rule out the possibility that the labeling may not occur to glycans, it is more likely a result caused by the low sensitivity of the detection method. Therefore, a more sensitive method to detect exosome is required to provide a more reliable answer to this result.

**Figure 4:**
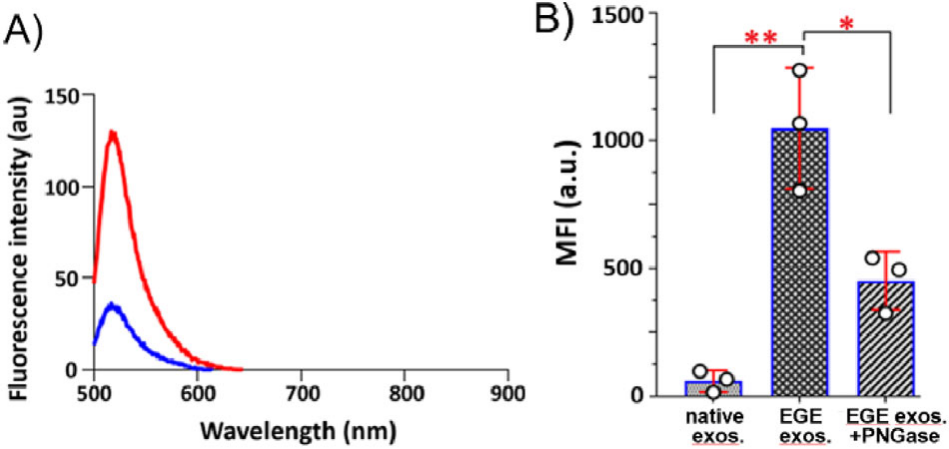
(A) Fluorescence spectra of EDTA‐recovered A488‐labeled exosome without (red) or with (blue) PNGase F treatment. (B) FACS MFI values of EDTA‐recovered native exosome and EGE‐modified exosome with or without PNGase F treatment. Data are shown as the average of three parallel experiments ± SD. Statistical significance levels: *p < 0.05, **p < 0.01.

Accordingly, we explored other methods to analyze EGE‐based labeling of exosomal glycans. One of them utilizes fluorescence labeling and analysis. In this regard, after biotinylated exosomes were treated with PNGase (or without for NC) and A488‐Strep on‐plate, the plate was treated with EDTA. Released exosomes were collected, concentrated ca. 100 times with a centrifugal filter unit, and then applied to fluorescence study. The fluorescence results (**Figure** **4**A) show that PNGase F treatment leads to a four‐fold decrease in the 488‐fluorescence intensity, proving the covalent linkage of A488 to exosomal glycoproteins and its removal upon PNGase treatment. This study also shows that treating exosomes within a single plate well with PNGase produces only weak signals (small changes in the OD value) that cannot be detected because of the low sensitivity of the previous analytical method.

Moreover, we performed FACS analysis of the released exosome. To this end, after on‐plate incubation of the biotinylated exosomes with PNGase and then Cy5‐Strep, they were released with EDTA, concentrated, and loaded onto aldehyde‐sulfate latex beads. The beads were treated with A488‐coupled anti‐CD63/CD81/CD9 Abs, resuspended in PBS, and finally studied with FACS. It is revealed that the beads show strong 488 fluorescence, indicating exosome attachment. Regarding the Cy5 fluorescence that was expected from glycans, the beads with EGE‐modified exosomes had 8‐fold higher mean fluorescence intensity (MFI) than native beads, and PNGase F treatment led to a significant decrease (by ≈2–3 folds) in the MFI (Figure 4B; Figure S14, Supporting Information). These results verify not only Cy5 labeling of EGE‐modified exosomes but also its attachment onto glycans, further supporting the above conclusion. However, some fluorescence remains after PNGase F treatment (Figure 4A,B), which can be attributed to glycolipids and *O*‐glycans that are not influenced by PNGase F, as well as *N*‐linked glycans resistant to PNGase, e.g., those with the fucosylated core.^[^ ^26^ ^]^

We also employed fluorescence imaging to validate EGE‐based fluorescence labeling of exosomes. We collected EDTA‐released exosomes that were incubated with Pd‐2,6‐ST, CMP‐Neu5Ac9N_3_, and A488‐Strep, attached them to a glass coverslip coated with anti‐CD9/CD63/CD81 Abs, and performed fluorescent imaging at 470/525 nm excitation/emission wavelengths. The imaging results (Figure S15, Supporting Information) disclose that EGE‐modified exosomes exhibit strong green fluorescence, but not exosomes without EGE treatment. The results further demonstrate the successful glycoengineering and fluorescence labeling of exosomes, as well as their EDTA‐mediated recovery from the plate.

Finally, we used Western blot to verify EGE‐enabled biotinylation of exosomal glycoproteins. In this study, we glycoengineered and biotinylated exosomes on‐plate and released them with EDTA. Exosomes were concentrated and lysed with the cell lysis buffer containing protease inhibitors. Proteins were isolated, suspended in PBS, and examined by sodium dodecyl sulfate‐polyacrylamide gel electrophoresis (SDS‐PAGE). Biotinylated proteins are shown via incubating the blot with A488‐Strep and then fluorescent gel imaging. The results (Figure S16, Supporting Information) disclose that glycoengineered exosomes, but not native ones, exhibit intense protein bands in Western blot, while for both samples, equal quantities of proteins are loaded into the gel (see silver staining), indicating successful engineering and biotinylation of exosomal glycoproteins.

#### 2.1.6 Stability of the Glycoengineered Exosomes

We used several approaches to study the stability of functionalized exosomes. First, we applied A488‐labeled exosomes to lyophilization, which did not have a major influence on the exosomal size, as indicated by DLS results (Figure S17, Supporting Information). Then, the exosomes were dissolved in PBS or in cell culture medium (OptiMEM + 10% FBS) and physiological condition‐mimicking fluids for prolonged time. DLS results (Figures S18 and S19, Supporting Information) show a slightly enlarged size (≈390 nm) in some cases, but not so much to alter their properties as exosomes. Moreover, as discussed later, exosomes before and after lyophilization or long‐term storage in PBS (for 14 d) exhibited similar endocytosis and cellular organelle targeting capacities.

### 2.2 EGE‐Enhanced Exosome Detection and Its Application to Exosomal Glycan Profiling

The above studies have established a robust and efficient method to tag exosomes based on enzymatic engineering of their glycans. Notably, on‐plate exosomal detection after glycoengineering was straightforward, but detecting native exosomes using labeled Abs under the same conditions was difficult. This suggests that EGE‐based exosome detection is more efficient than Ab‐based ones. We have also confirmed that the commonly used methods to label exosomes with PKH67 (a membrane lipid label)^[^ ^27^ ^]^ and A488‐NHS (a surface protein label) are also less efficient (Figure S20, Supporting Information). These results demonstrate an EGE‐based sensitive detection approach for exosomes. Then, we became interested in gaining insights into exosome EGE and the properties of engineered exosomes using this effective labeling/detection approach.

First, we evaluated the limit of this EGE‐based exosome‐detecting method. We found that plates coated with 1.25 µg mL^−1^ exosomes, which was lower than 5 µg mL^−1^ used earlier, also produced strong signals (Figure S21A, Supporting Information), but at further reduced concentrations, no significant difference from NCs was observed. This suggests a detection limit of 1.25 µg mL^−1^ under current conditions. We proved that this is a concentration way beyond the detection range by the conventional methods using Abs. For example, labeling the plate pretreated with 2.50, 3.75, and 5.00 µg mL^−1^ of exosomes using biotinylated anti‐CD63/CD81/CD9 Abs and analyzing the plates using AP‐Strep/PNPP exhibited no OD changes when compared to NCs (Figure S21B, Supporting Information). In contrast, EGE‐based exosome labeling resulted in strong signals at all three concentrations.

**Figure 5:**
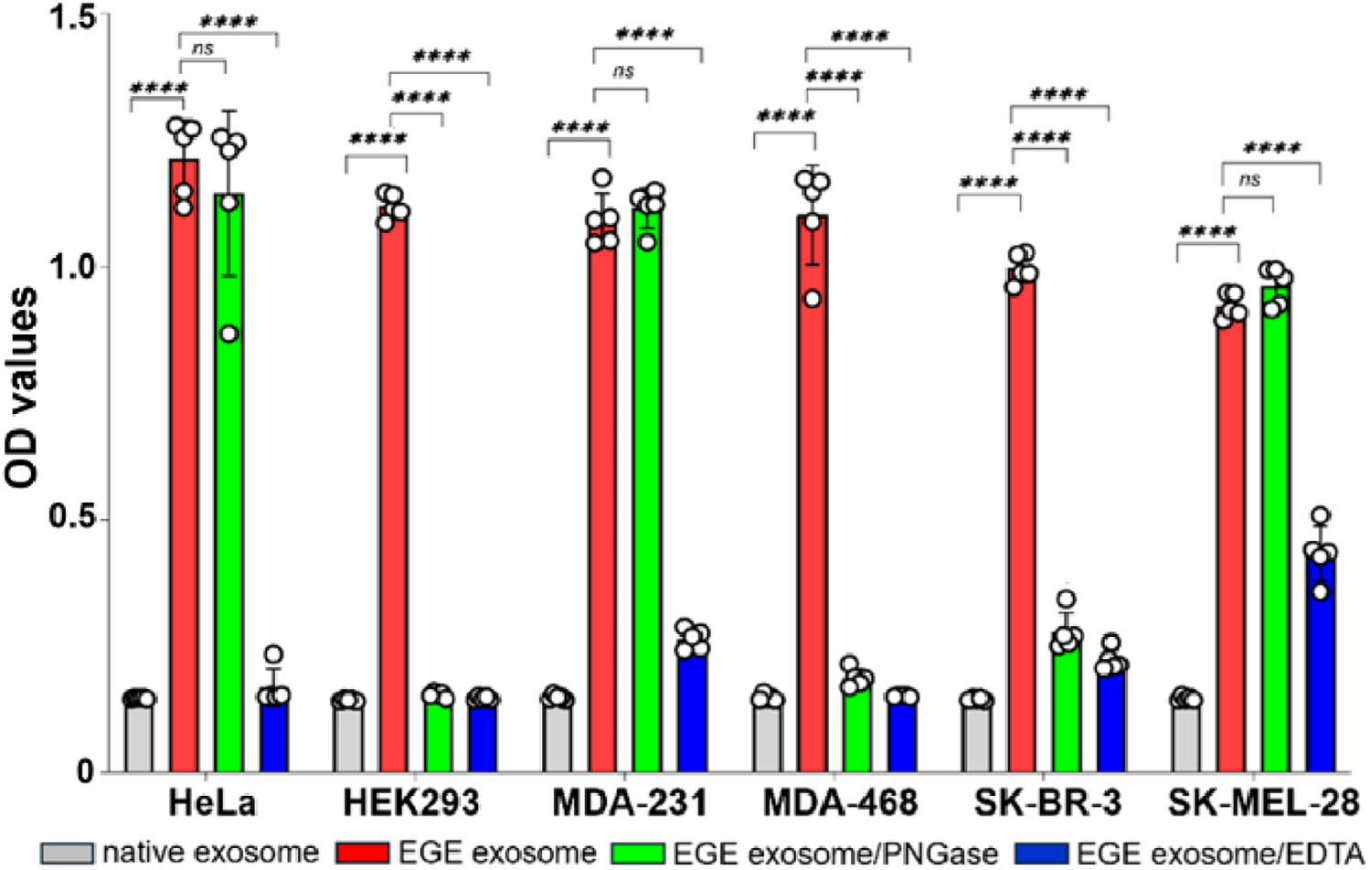
Efficiencies of EGE‐based labeling of exosomes derived from different cell lines and their glycan profiles revealed by EGE, PNGase F‐based *N*‐glycan release, and EDTA‐mediated release of exosome. Data are shown as the average of five parallel experiments ± SD. Statistical significance levels: ns (not significant) *p* > 0.5; ****p < 0.0001.

In theory, this novel EGE‐based exosome labeling method should be applicable to other exosomes too. To verify, we examined the EGE and labeling of commercial exosomes from different sources, such as HEK293, MDA‐MB‐231, MDA‐MB‐468, SK‐BR‐3, and SK‐MEL‐28 cells. Our results (**Figure** **5**; Figure S22, Supporting Information) verify that all these exosomes show significantly increased OD values after EGE and labeling by above protocols, as compared to corresponding native exosomes, proving their successful EGE. Notably, discrepancies in signal intensities are found with the modified exosomes (Figure 5). The results may indicate varied glycosylation patterns among the exosomes of different origins, agreeing with the reports that different cells have different glycan patterns.^[^ ^11^, ^12^, ^28^ ^]^

To gain more insights into the glycan profiles of various exosomes, we incubated biotinylated exosomes from different cell lines with PNGase F (EGE/PNGase exosomes) before treatment with AP‐Strep/PNPP and colorimetric analysis. The results (Figure 5; Figure S22, Supporting Information) were intriguing. Upon PNGase treatment, like HS exosome, exosomes from HeLa, MDA‐MB‐231, and SK‐MEL‐28 cells show no significant decreases in OD (Figure S13, Supporting Information), but exosomes from HEK293, MDA‐MB‐468, and SK‐BR‐3 cells respond particularly well to PNGase, resulting in a decrease in the OD value to almost background level. The results reveal great variations in the glycan profile of tested exosomes. Although, as with HS exosome, no clear OD change after PNGase treatment for the former group of exosomes does not necessarily mean no *N*‐glycan labeling, this result undoubtedly suggests that labeled glycans on exosomes of these cells are more resistant to PNGase F than others. Therefore, EGE of HEK293, MDA‐MB‐468, and SK‐BR‐3 exosomes seems to occur mainly to their *N*‐glycans, while EGE of HS, HeLa, MDA‐MB‐231, and SK‐MEL‐28 exosomes occurs, to a large extent, to their glycolipids, *O*‐glycans, and PNGase‐resistant *N*‐glycans.^[^ ^26b^ ^]^ Clearly, various exosomes possess very different glycan profiles. This finding deserves more detailed investigation that may shed light on the biological significance of exosome surface glycans as well as their functional mechanisms.^[^ ^11^, ^29^ ^]^

The above‐biotinylated exosomes were also incubated with EDTA before treatment with AP‐Strep/PNPP and colorimetric analysis. The results (Figure 5; Figure S22, Supporting Information) disclose significant decreases in the OD value in all cases, proving the successful on‐plate labeling of exosomes, as observed in the experiments above, and their EDTA‐mediated release from the plate. Interestingly, after EDTA treatment, a substantial number of SK‐MEL‐28 exosomes remain on the plate, possibly indicating their interaction with the plate via molecules other than between PS and A5.

### 2.3 Investigation into Cellular Uptake of Exosomes

**Figure 6:**
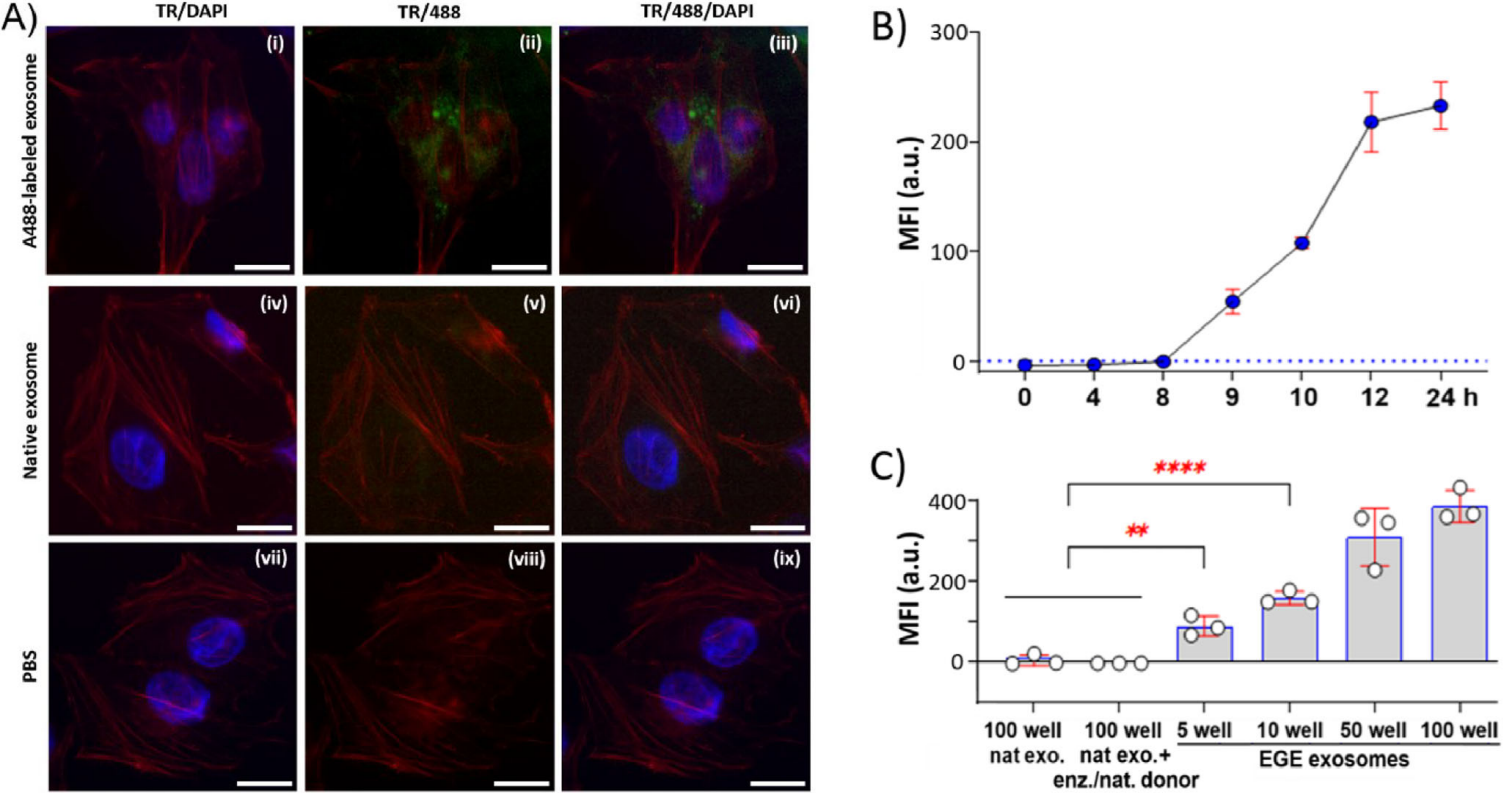
(A) Fluorescence images of HeLa cells incubated with A488‐exosome, native exosome, or PBS (10 h), followed by actin network and cell nucleus staining. TR: actin; DAPI: cell nucleus; 488: exosome; Scale bar: 20 µm. FACS MFI of HeLa cells incubated with (B) A488‐exosome for varied time and (C) native exosome, Pd‐2,6‐ST/native sialyl donor‐treated exosome, or varied concentrations of A488‐exosome. Data are presented as the average of three parallel experiments ± SD. Statistical significance levels: **p < 0.01; ****p < 0.0001.

After EGE‐based exosomal labeling was validated, we applied the method to access biologically useful exosomes, e.g., for exploring exosome endocytosis by cells with fluorescence microscopy and FACS. On‐plate exosome EGE, A488 labeling, retrieving, pooling, and concentration followed the same protocols as above. HeLa cell was the model. Our fluorescence imaging results (**Figure** **6**A; Figure S23, Supporting Information) show that after 10 h of incubation with A488‐exosomes and extensive washing, cells exhibit strong fluorescence (Figure 6Ai–iii), which contrasts to cells exposed to native exosomes and PBS (iv‐ix), proving cellular uptake of A488‐exosomes.

Next, we examined the kinetics of cellular uptake of exosomes. In this context, we incubated cells with A488‐exosomes for different lengths of time (0–24 h). Fluorescence imaging (Figure S24, Supporting Information) and FACS (Figure 6B; Figure S25, Supporting Information) studies reveal that after ca. 8–9 h of incubation, exosome uptake becomes substantial, which agrees with previous reports.^[^ ^30^ ^]^ Furthermore, exosome uptake begins to reach a plateau after 24 h of incubation under the given condition. We also examined the impacts of exosome concentration on the uptake using FACS. As expected, the MFIs of cells incubated with A488‐labeled exosomes increase in an exosome concentration‐dependent fashion (Figure 6C; Figure S26, Supporting Information).

**Figure 7:**
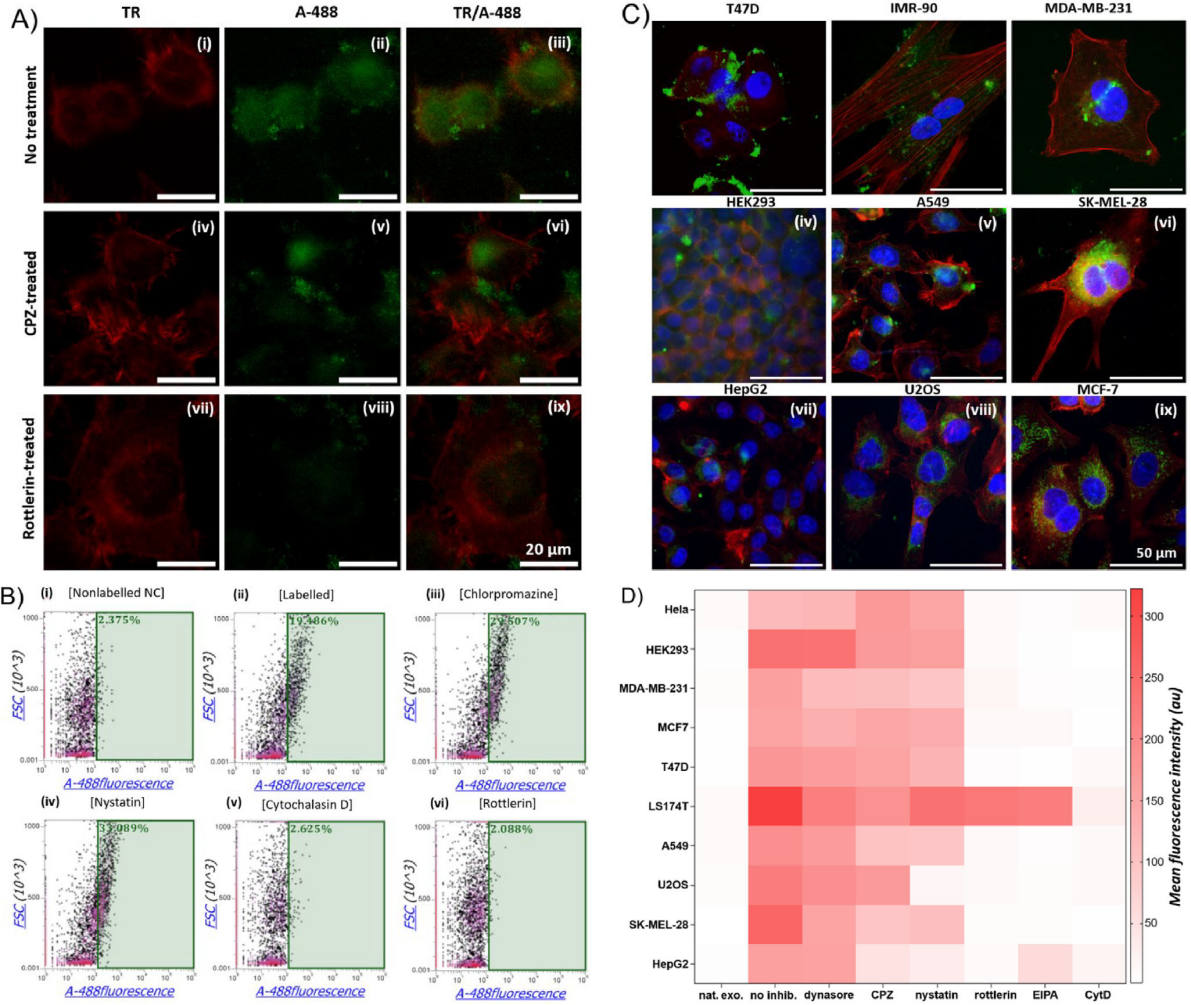
(A) Fluorescent micrographs of HeLa cells incubated with A488‐exosome (10 h) without uptake inhibitor or with clathrin inhibitor CPZ and macropinocytosis inhibitor rottlerin. TR: actin network; A488: exosome fluorescence; TR/A488: overlaps. (B) Single fluorescence (A488) FACS density profiles of HeLa cells incubated with native exosome (nonlabelled) or A488‐exosome (10 h) without or with CPZ, nystatin, CytD, and rottlerin. The cell densities in the positive gate are denoted at the top left corner. (C) Fluorescent micrographs of various cells after incubation with A488‐exosome for 10 h, followed by staining the actin network and cell nucleus. Blue: nucleus; red: actin network; green: exosome. (D) FACS MFI values of cells treated with native or A488‐exosome (10 h) without uptake inhibitor or with inhibitors dynasore, CPZ, nystatin, rottlerin, EIPA, and CytD.

To confirm that exosomes were indeed endocytosed rather than nonspecifically adhered to cells, we conducted in‐depth analyses using endocytosis inhibitors.^[^ ^31^ ^]^ Therefore, prior to exosomes, we pretreated cells with clathrin‐ and caveolin‐mediated endocytosis inhibitors chlorpromazine (CPZ) and nystatin, respectively, and macropinocytosis inhibitors rottlerin, cytochalasin D (CytD), and 5‐(*N*‐ethyl‐*N*‐isopropyl)amiloride (EIPA) (Table S1, Supporting Information). Fluorescence imaging (**Figure** **7**A; Figure S27, Supporting Information) and FACS (Figure 7B; Figure S28, Supporting Information) results demonstrate that inhibiting macropinocytosis, instead of other pathways, blocks exosome endocytosis (Figure 7A‐vii,B‐v/vi), suggesting macropinocytosis‐mediated endocytosis by Hela cells. Macropinocytosis is an actin‐mediated process for nonspecific uptake of extracellular fluid and its contents facilitated by plasma membrane ruffles and cups. Our results corroborate earlier findings that inhibiting actin dynamics impedes exosome uptake by recipient cells.^[^ ^32^ ^]^

The retention time or clearance rate of labeled exosomes in cells was also studied. In this context, after cells were incubated with EGE‐modified A488‐tagged exosomes (10 h), they were washed and cultured in a low serum media (OptiMEM/0.5% FBS) without exosome. The fluorescence of cells was analyzed. FACS results (Figures S29 and S30, Supporting Information) indicate that these exosomes have a rather low clearance rate, with a half‐life time of 8–9 h. Fluorescent imaging results (Figure S31, Supporting Information) agree with FACS results.

Furthermore, we also examined the uptake of exosomes by other cell lines, e.g., IMR‐90, MDA‐MB‐231, HEK293, A549, SK‐MEL‐28, T47D, HepG2, U2OS, and MCF7. Fluorescent imaging results (Figure 7C; Figure S32, Supporting Information) demonstrate the efficient uptake of A488‐exosomes by these cells, underscoring the generality of the above protocols. In addition, FACS results suggest that the cells exhibit distinctive properties in the presence of inhibitors for endocytosis (Figure 7D; Figures S33–S41, Supporting Information), indicating the potential involvement of different endocytosis pathways. Although all cells rely mainly on macropinocytosis, other endocytosis pathways are also important in certain cells. For instance, endocytosis occurs at least partially via caveolin‐ and lipid raft‐mediated pathways and phagocytosis in HEK293 cells. Tested macropinocytosis inhibitors also exhibit unique influences on exosome uptake in LS174T cells. Therefore, exosome uptakes may engage complex and diverse mechanisms, a topic worth more detailed investigation. Nonetheless, our results together with those of others collectively indicate that the primary pathway for exosome uptake is macropinocytosis, whereas other pathways are also involved, to different extents, depending on the cell lines.

**Figure 8:**
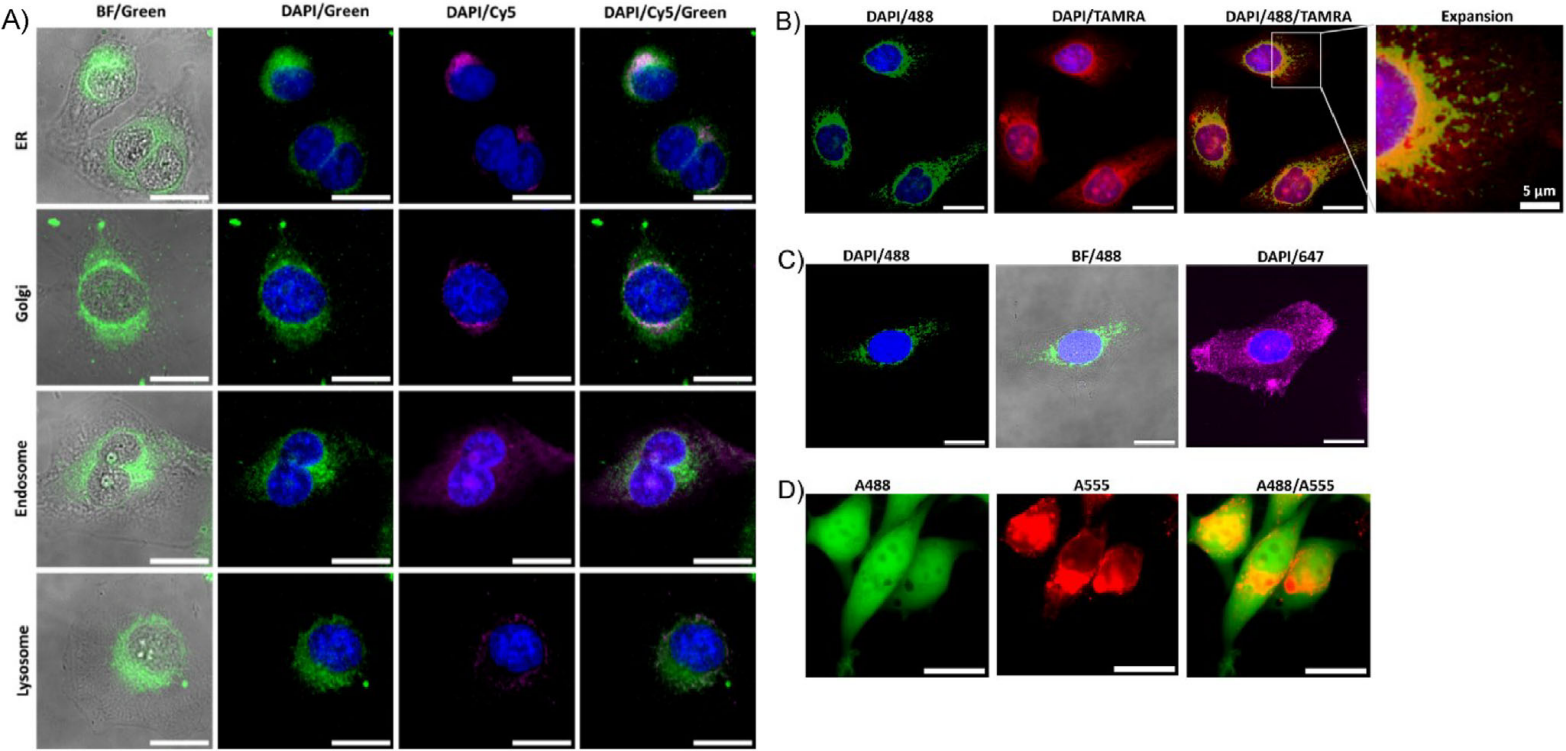
(A) Colocalization of EGE‐modified A488‐exosomes (green) with the cell nucleus (DAPI, blue) and other intracellular organelles, including ER, Golgi, late endosome, and lysosome (Cy5, red). (B) Colocalization of the dually labeled exosomes carrying A488‐glycan and TAMRA‐protein (red) with the cell nucleus. (C) Fluorescence images of endocytosed protein transferrin‐647 (magenta) and A488‐exosome, along with the cell nucleus. (D) Fluorescence images of A488‐labeled surface proteins on/in cells and endocytosed EGE‐modified A555 (red)‐exosome. Scale bar: 20 µm or specified otherwise.

Another notable observation during the study of cellular uptake of exosomes is their nonuniform distribution within the cytoplasm, as they appear to be concentrated in the region near the cell nucleus. This piqued our interest in profiling cellular organelles colocalizing with endocytosed exosomes. Hence, we fixed and permeabilized cells after A488‐exosome treatment and stained their intracellular organelles with Cy5‐Abs of proteins associated with endoplasmic reticulum (ER), Golgi, late endosome, and lysosome. Fluorescent imaging results (**Figure** **8**) indicate the colocalization of A488 with Cy5, especially in ER, Golgi, and lysosome, which are adjacent to or around the cell nucleus. The results suggest that endocytosed exosomes may preferably interact with the intracellular organelles, potentially facilitating the delivery of payloads to these locations, which is partially supported by literature results.^[^ ^30^, ^33^ ^]^

To verify the conclusion, we utilized TAMRA (red)‐NHS^[^ ^34^ ^]^ to non‐selectively label the surface proteins of EGE‐modified exosomes that carried the A488 tag. Treatment of HeLa cells with this dually tagged exosome resulted in the colocalization of both fluorescent tags around the cell nucleus (Figure 8b; Figures S42–S44, Supporting Information). In contrast, treatment of HeLa cells with A488‐exosome and A647 (magenta)‐labeled transferrin, an easily endocytosed exogenous protein,^[^ ^35^ ^]^ did not exhibit colocalization of the two tags (Figure 8c; Figure S45, Supporting Information). These results have validated not only the preferred localization of exosomes around or in the cell nucleus but also their stability and endocytosis as a whole, since if exosomes are degraded before entering cells or quickly after, we should expect a wider spread of the two tags. In addition, we also studied the endocytosis of EGE‐modified A555‐exosomes by cells having surface proteins labeled with A488 and found that A488 had a uniform distribution on or in cells, while A555‐exosome (red) was confined to the perinuclear region (Figure 8d; Figure S46, Supporting Information). The results further prove the selectivity of exosomal endocytosis and suggest that labeling cell surface proteins does not have a major impact on exosomal endocytosis. Finally, we observed that after lyophilization or storage in PBS at 37 °C for 14 d, EGE‐modified A488‐exosomes still possessed the same endocytosis property as freshly prepared exosomes (Figure S47, Supporting Information), which provides additional support for the superior stability of the functionalized exosomes.

## 3 Conclusion

In this research, we have developed a novel EGE‐based method for on‐plate engineering of exosomes. It is based upon enzymatic modifications of exosomal glycans using a Sia‐T, together with an azide‐modified sialyl donor, to introduce azido‐sialic acids to the exosome surface as molecular handles. This enables the linkage of other functionalities, e.g., alkynylated biotin or fluorophores as affinity and fluorescent labels, to the azido‐glycans on exosomes by biocompatible click chemistry. Protocols for the attachment of exosome to polystyrene plate and subsequent on‐plate EGE and functionalization, as well as EDTA‐mediated release of exosomes from the plate, are optimized and streamlined to make this new exosome engineering approach fast, simple, and robust. It is also proved to be effective and consistent in many studies and different exosomes. In addition, we have shown that exosome EGE can be controlled via adjusting the enzyme and substrate concentrations or reaction time. This method makes it possible to study functional exosomes conveniently on‐plate and access functional exosomes in practical quantity for off‐plate in‐depth analyses, e.g., DLS and colorimetric and FACS studies, as well as applications.

We have demonstrated that the EGE‐based method for exosomal labeling produces better signals (by 2–10 folds, Figure S20, Supporting Information) than the methods based on direct modification of exosomal lipids and proteins. In the meantime, glycans are usually more extruded and structurally flexible than lipids or proteins on the exosome surface, facilitating their further modification with large biomolecules, such as proteins and antibodies. On the other hand, compared to MGE‐based exosomal modification, the EGE‐based method is cell‐free and, therefore, more convenient and widely applicable. Moreover, glycan‐, lipid‐ and protein‐based methods are orthogonal, thereby enabling exosome functionalization by different molecules/labels. Thus, this new EGE‐based method for exosome functionalization will find broad and unique applications.

It has been shown that EGE‐based biotinylation and modifications make the detection, observation, and analysis of exosome easier and more flexible and sensitive by various analytical techniques, thereby facilitating more detailed studies. Consequently, we could disclose that, during EGE, exosomes from various cell lines had different sensitivities toward Pd‐2,6‐ST, suggesting the potential difference in glycan composition of these exosomes as Pd‐2,6‐ST substrates. This was further validated by the significantly different responses of engineered exosomes to PNGase F that selectively hydrolyzes glycoprotein *N*‐glycans. For example, EGE‐modified exosomes from HEK293, MDA‐MB‐468, and SK‐BR‐3 cells are highly responsive to PNGase, thus treating these exosomes with PNGase leads to nearly complete removal of all modified glycans, indicating that Pd‐2,6‐ST‐mediated Neu5Ac9N_3_ linkage to these exosomes occurs mainly to *N*‐glycans. In contrast, the HS, HeLa, MDA‐MB‐231, and SK‐MEL‐28 exosomes only partly respond to PNGase F, resulting in partial removal of functionalized glycans, indicating that Pd‐2,6‐ST‐mediated Neu5Ac9N_3_ addition occurs to both *N*‐ and *O*‐glycans and glycolipids in these exosomes. Clearly, exosomes of different origins have distinctive glycan profiles. It is well‐recognized that various cell types can have different glycan patterns, but how much exosomal and donor cell glycan profiles are correlated is still unclear. On the other hand, an answer to this question is important to better understand the functional roles and mechanisms of exosomal glycans. For example, it is reported that *N*‐linked glycans are a determinant for exosome or microvesicle cargo recruitment.^[^ ^36^ ^]^ Thus, detailed profiles of glycans of various exosomes and comparisons of them and to the glycan profiles of donor cells deserve further investigation. This topic is out of scope for the current work that mainly focuses on the development of methods to functionalize exosomes but will be investigated in our lab in the future.

The high sensitivity of this EGE‐based method to detect exosome, as well as the easy access to practical quantities of functionalized exosomes by the new exosome functionalization method, enables our in‐depth studies of exosomes, such as their cellular uptake, using both fluorescence microscopy and FACS. We have verified the effective uptake of functionalized exosomes by various cells, which reach plateaus after 24 h of incubation. Significantly, helped by the inhibitors of different endocytosis pathways, we can gain a better understanding of the uptake mechanisms of exosomes. For the first time, we have provided direct proof that, in all tested cells, macropinocytosis is the main exosome endocytosis mechanism, although other mechanisms may also be involved in some cells. This finding agrees with the literature results.^[^ ^32^ ^]^ In addition, there are also reports that glycans of exosomes are directly involved in their cellular uptake.^[^ ^29^ ^]^ These functionalized exosomes combined with Ab‐mediated intracellular organelle staining are used to show that the internalized exosomes are mainly colocalized with organelles around the cell nucleus. Since where exosomes go after cellular uptake is critical for their payload delivery and other application,^[^ ^37^ ^]^ the finding is important and useful to not only help understand the properties and functions of exosomes but also promote their application. For example, the results can help design new targeted drug delivery systems and cancer immunotherapies using specific exosomes, a direction currently pursued in our lab.

## 4 Experimental Section

### Materials

Nunc 96‐round bottom‐well polystyrene plate, EDTA, aldehyde/sulfate latex beads (4 µm, 4% w/v), the Halt protease inhibitor cocktail (10x), OptiMEM media, glycine, and 0.2 m buffer sol^n^ (pH 2.5) are purchased from ThermoFisher scientific. Recombinant A5 and A5 binding buffer (10x) were purchased from BD Bioscience. Lyophilized exosomes of various origins were purchased from Hansa BioMed (accompanied by Certificate of Analysis–see a sample in Supporting Information) and verified to have the size of 90–100 nm by DLS in the lab. They were dissolved in 1x filtered PBS to form 0.1 mg mL^−1^ stock solutions for various experiments. The coating buffer is 0.1 m NaHCO_3_ (pH 9.6) containing 0.01% (w/v) sodium azide. PBST for washing consists of 1x PBS and 0.05% Tween‐20, which was filtered before usage. The blocking solution is 1% (w/w) BSA in PBST with 0.01% (w/v) sodium azide. PNPP buffer is 0.1 m NaHCO_3_ containing 2.5 m MgCl_2_ (pH 10.3). Enzymatic reaction buffer comprises 100 mm Tris·HCl and 20 mm MgCl_2_ with a final pH value of 8.0–9.0. TBST used for Western blot contains 10 mm Tris·HCl (pH 7.4), 0.9% (w/v) NaCl, and 0.02% (v/v) Tween 20. FACS buffer comprises 1x PBS, 5.0% (w/v) BSA, and 0.5% (w/v) NaN_3_. Protein buffer 1 for Ab storage contains 10 mm sodium HEPES (pH 7.5), 150 mm NaCl, 100 µg mL^−1^ BSA, 50% glycerol, and 0.01% (w/v) NaN_3_. Protein buffer 2 used for the reactions between Ab and DBCO‐NHS ester has the same recipe but is slightly more basic (pH 8.0–8.5). PNGase F was purchased from New England Biolabs. The mammalian cell lysis buffer was from GoldBio. Amicon Ultra Centrifugal Filter (3 kDa MWCO) and (3‐aminopropyl)triethoxysilane (APTES) were from Millipore Sigma. µ‐Slide 8‐well coverslip was purchased from ibidi. Glass coverslip (22 × 22 × 1.5 mm) used in fluorescent imaging was purchased from Fisherband.

Neu5Ac9N_3_ was chemically prepared on multi‐gram scales in an excellent overall yield, and CMP‐Neu5Ac9N_3_ was enzymatically synthesized from Neu5Ac9N_3_ before usage, both by previously reported procedures as shown in Scheme S1 (Supporting Information).^[^ ^16^ ^]^ For the synthesis of latter, Neu5Ac9N_3_ (200 mg) was added to a solution of cytidine triphosphate (CTP) (5.1 equiv, ThermoFisher) in Tris buffer (10 mm, pH 8.8, 500 µL) containing 20 mm MgCl_2_, 0.1 unit mL^−1^ inorganic pyrophosphatase (1.0 unit µL^−1^ stock, 0.5 µL, ThermoFisher), and 0.2 unit mL^−1^ CMP‐sialic acid synthetase NmCSS (10 Unit mL^−1^ stock, 10 µL, Chemily Glycoscience), and the pH value was maintained at 8.0–9.0. The reaction was incubated at 37 °C with shaking (200 rpm) for 5 h and monitored with TLC (elution system: EtOH/1 m NH_4_HCO_3_ 7:3, v/v). Once the reaction was completed, ice‐cold MeOH was added, and the mixture was centrifuged at 4000×g for 15 min. This MeOH addition and centrifugation protocol was repeated two more times before the supernatant was concentrated and resuspended in Tris buffer (2.0 mL). The solution was loaded onto a pre‐equilibrated BioGel P‐2 (Bio‐Rad) column and eluted with double‐distilled water (10 mL elution volume/tube). Fractions containing the desired product were combined and lyophilized to give CMP‐Neu5Ac9N_3_ (167 mg, 44%), which was stored at −20 °C. Their NMR and MS spectra (Figures S48–S52, Supporting Information) matched with those reported for Neu5Ac9N_3_ and CMP‐Neu5Ac9N_3_ in the literature, and their purities were >99 and 90%, respectively. Typically, a 0.1 g mL^−1^ stock solution of CMP‐Neu5Ac9N_3_ in Tris buffer (pH 8.0) was freshly prepared before each experiment and used for enzymatic reactions within 12 h.

Pd‐2,6‐ST was prepared according to a reported method^[^ ^38^ ^]^ using a pET22b vector with the gene custom‐synthesized by GeneArt of ThermoFisher Scientific. Purified Pd‐2,6‐ST was stored in Tris buffer (pH 8.0) at a concentration of 0.5 mg mL^−1^ at 4 °C for short‐term (≈2 weeks) and at −80 °C for long‐term in 50 µL aliquots that were used without undergoing a freeze‐thaw cycle.

HeLa, HEK293, IMR‐90, MDA‐MB‐231, T47D, U2OS, MCF7, LS174T, HepG2, SK‐MEL‐28 and A549 cell lines, culture media including DMEM, Leibovitz's L‐15, RPMI‐1640, McCoy's 5A, F‐12K and Eagle's minimum essential medium (EMEM), and other chemicals such as streptomycin, penicillin and fetal bovine serum (FBS) were purchased from ATCC.

A BioTek plate reader Cytation 1 was utilized for plate‐based colorimetric or fluorescent analyses. For fluorescent imaging, an Olympus IX71 inverted system equipped with LED light (Cool LED, PE‐300), 40×0.75 plan apochromatic objectives, DAPI (Ex/Em: 365/470), green (Ex/Em: 488/530), red (Ex/Em: 565/640) and Cy5 (Ex/Em: 647/685) fluorescence channels, and an Olympus DP23 M color camera. Fluorescent image and intensity analysis was accomplished with Olympus Cellsens Standard 3 software and FIJI/ImageJ software. FACS was carried out with an Attune NxT flow cytometer equipped with blue (Ex: 488 nm) and red (Ex: 638 nm) lasers, and data analyses were performed with the accompanied Attune NxT flow cytometer software. For Western blot, an Invitrogen iBlot2 instrument was used to transfer proteins from gel to membrane.

### Antibody‐Based on‐Plate Exosome Detection

Coating buffer (200 µL) with A5 (0.5 mg mL^−1^ stock solution, 2 µL) was added to each well of a 96‐well plate. The plate was incubated at 4 °C overnight and then at 37 °C for 1 h and washed 3 times with PBST. Blocking solution (300 µL) was added to each well, and the plate was incubated at room temperature (rt) for 45 min, followed by washing three times with PBST. Then, HS exosomes (0.1 mg mL^−1^ stock solution, 10 µL) were added to each well, followed by A5 binding buffer (1x) to make up the volume to 200 µL. The plate was incubated at 37 °C for 2 h with slow shaking (100 rpm) and washed three times with PBST. To detect the attached exosomes, to each well was added PBS (200 µL) containing biotinylated anti‐CD63/CD81/CD9 Abs (5 µg mL^−1^ each, from a 1.0 mg mL^−1^ stock solution in protein buffer 1). The plate was incubated at rt for 1 h with gentle swirling (100 rpm) and then washed 3 times with PBST. Thereafter, AP‐Strep (5 µg mL^−1^) in PBS (10 mg mL^−1^ stock, 200 µL) was added to each well, and the plate was incubated at rt for 1 h with swirling (100 rpm) and washed thoroughly (nine times) with PBST. Finally, a fresh PNPP solution (200 µL, 2 mg mL^−1^ in PNPP buffer) was added to each well, and the plate was incubated at rt for 1 min and finally subjected to colorimetric readout at 405 nm wavelength using a BioTek plate reader.

### Exosome EGE and Colorimetric and Fluorometric Detection of Engineered Exosomes

HS exosomes were attached to the plate and washed by the same procedure as described above. Then, a solution of 1.67 mg mL^−1^ CMP‐Neu5Ac9N_3_ (5.0 µL, 0.1g mL^−1^ stock) and 3.33 µg mL^−1^ Pd‐2,6‐ST (2 µL, 0.5 mg mL^−1^ stock) in 300 µL of enzymatic reaction buffer was added to each well. The plate was incubated at 37 °C for 1 h with swirling at 100 rpm and washed three times. DBCO‐biotin (100 µm) (0.4 µL, 50 mm stock in 200 µL of Tris buffer, pH 7.5) was added to each well, and the plate was incubated at rt for 1.5 h with gentle swirling at 100 rpm followed by three times of washing.

For colorimetric detection of exosomes, PBS (200 µL) containing 5 µg mL^−1^ AP‐Strep (0.1 µL, 10 mg mL^−1^ stock in PBS) was added to each well, and the plate was incubated at rt in dark for 1 h with swirling (100 rpm) and washed nine times. Fresh PNPP solution (200 µL, 2 mg mL^−1^ in PNPP buffer) was added to each well, and the plate was incubated at rt for 1 min and subjected to colorimetric readout at 405 nm wavelength.

For fluorometric detection of exosomes, PBS (200 µL) containing 5 µg mL^−1^ A488‐Strep (1 µL, 1 mg mL^−1^ stock) was added to each well, and the plate was incubated at rt in dark for 1 h with swirling (100 rpm) and washed nine times. Each well was replenished with Tris (200 µL) buffer for 488‐fluorescence recording with a Cytation 1 plate reader using the green filter (Ex/Em: 488/530 nm). In negative controls, exosomes were treated by the same procedure but having CMP‐Neu5Ac9N_3_ and Pd‐2,6‐ST replaced with PBS or heat‐deactivated Pd‐2,6‐ST.

### Exosome Recovery from Plates

HS exosomes were attached to the plate, glycoengineered, and biotinylated (and further labeled with A488‐Strep) by the same protocols described above. Then, Tris buffer (200 µL, pH 8.0) containing 10 mm EDTA (2 µL, 1 m stock solution in Tris buffer) was added to each well, and the plate was incubated at 37 °C for 1.5 h while swirling at 100 rpm. The supernatant from each well was collected and put in a sterilized centrifugal filter unit (3 kDa MW cut‐off) for concentration through centrifugation (2000 rpm) at 4 °C. The exosomes were washed three times with PBS in the same filter unit.

### Preparation of Glycan and Protein Dually Labeled Exosomes

After exosomes were attached to plates and EGE‐modified and then labeled with A488 fluorophore using the protocol described above, each plate well was incubated with coating buffer (200 µL) containing 150 µm of TAMRA‐NHS (1.5 µL, 20 mm stock solution in DMSO, Biotium) with gentle swirling (100 rpm) at rt for 2 h. The plate was washed 3 times with PBST, and the exosomes were then recovered from the plate via EDTA treatment by the protocol described earlier. After the exosomes were washed three times with filtered PBS using a 100 kDa ultrafiltration tube, the solution was collected and applied for the studies described below.

### Labeling of Exosome Lipids^[^ ^27^ ^]^

A 20 µm stock solution of PKH67 dye (5.0 µL) in diluent C (Sigma Aldrich) was added to exosomes (10 µg) suspended in diluent C (0.5 mL), which was well mixed by pipetting. The solution was then incubated in the dark at rt for 20 min. For the negative control, the diluted dye was mixed with PBS only. Thereafter, 1% BSA in PBS (2 mL) was added to both tubes, and the mixtures were centrifuged at 120 000 × *g* and 4 °C for 1 h. The supernatant was discarded, and the pellet was resuspended in PBS buffer (2.0 mL) and centrifuged again at 120 000 × *g* at 4 °C for 2 h. Then, the washing step with PBS was repeated two more times, and finally, the pellet of the exosomes was reconstituted in 0.3 mL of filtered PBS.

### PNGase F Treatment of Exosomes

HS exosome was attached to the plate and glycoengineered by the same protocols described above. Then, glycobuffer 2 (20 µL) and 10 000 units of PNGase F (20 µL, 500 000 units mL^−1^ stock) were added to each well, and the volume was made up to 200 µL using 1xPBS. The plate was incubated at 37 °C for 1.5 h while swirling at 100 rpm and washed three times with PBST. Then, each well was subjected to sequential treatments with DBCO‐biotin and AP‐Strep/PNPP and finally colorimetric readout as described. In negative controls, exosomes were manipulated by the same procedure but having PNGase F replaced with PBS.

### Dynamic Light Scattering Analysis of Exosomes

Released and concentrated exosomes from five plate wells (each containing 1.0 µg exosomes initially) in PBS (50 µL) were diluted to a final volume of 500 µL using PBS buffer and then filtered three times through 0.22 µm PDVF filters. The solution was subjected to DLS analysis on a Malvern DLS instrument.

### Fluorescence Imaging of Exosomes on Coverslips

Ten wells of A488‐labeled exosomes (1.0 µg per well, initially) were prepared, collected, and concentrated in 50 µL of PBS as described. In the meantime, a glass coverslip was cleaned by etching at rt in 1 N HCl and then 1 N NaOH for 30 min each under sterile conditions. The coverslip was washed three times with Dulbecco's PBS and treated with 100% ethanol (2.0 mL) for 30 min. The coverslip was activated by treatment with 2% APTES in ethanol (400 µL) at 60 °C for 1 h, washed with Dulbecco's PBS, and dried. The activated coverslip was placed in a 35 mm dish containing 1.0 mL of anti‐CD63, CD81, and CD9 Ab solution (each 10 µg mL^−1^ from a 1.0 mg mL^−1^ stock solution), followed by incubation at rt overnight. The coverslip was washed three times with PBS (1.0 mL) and incubated with above exosomes in PBS (1.0 mL) at 4 °C in dark overnight. The coverslip was washed thoroughly with PBS and imaged with the GFP fluorescence channel (Ex/Em: 488/530 nm).

### FACS Analysis of Exosomes

FACS analysis of exosomes was achieved after their absorption to aldehyde/sulfate latex beads (4 µm) following previously reported protocols.^[^ ^39^ ^]^ Briefly, latex beads (5 µL, 39 mg mL^−1^ commercial stock) were washed with 1xPBS (1.0 mL) by vortexing, followed by centrifugation at 5000 g for 30 min. The beads were suspended in 1xPBS (500 µL) containing 0.5% BSA. In the meantime, native or glycoengineered exosomes were treated with DBCO‐biotin, PNGase F, or PBS, and then Strep‐Cy5 as mentioned, followed by EDTA treatment to release exosomes. Released exosomes were collected from 100 wells (each initially containing 1 µg exosomes) and concentrated. The concentrated exosomes were suspended in filtered PBS (100 µL) containing 0.5% BSA, added to the bead solution, and incubated at rt for 1 h, followed by overnight incubation at 4 °C with constant shaking. The beads were centrifuged at 21 000 g for 90 min, resuspended in 1 m glycine (500 µL), and incubated at rt for 45 min with gentle shaking. The beads were centrifuged at 21 000 g for 30 min, and the pellet was resuspended in PBS (500 µL) with 0.5% BSA for 30 min and centrifuged again as mentioned above to produce the bead pellet. The beads were then incubated with 488‐conjugated ExoBrite antibodies (Table S2, Supporting Information, entries 3a–d) in PBS (100 µL) containing 0.5% BSA at rt for 1.5 h. After incubation, the beads were washed with PBS three times and resuspended in PBS (1 mL), followed by analysis using a flow cytometer. Side scattering (SSC) was used for all experiments with 300 and 310 Volts for forward scattering (FSC), and a minimum of 15000 events were recorded in each experiment. A FSC area versus FSC height density plot was used to exclude latex bead doublets. Blue and red excitation lasers with emission at 530 ± 30 (BL1 channel) and 670 ± 14 nm (RL1 channel) were used to detect the 488 and Cy5 fluorescence, respectively. The Attune NXT software was used for analysis of FACS data.

### Western Blot

Biotinylated exosomes and the negative controls collected from 100 plate wells (each containing 1 µg exosomes) were concentrated, washed, and resuspended in PBS (100 µL). To this solution was added mammalian cell lysis buffer (400 µL) containing Halt protease inhibitor cocktail (10x, 0.5 µL), followed by incubation on ice for 1 h, sonication with a Qsonica probe sonicator (6 pulses, 60% duty cycle, 30 s each, Amp10), and centrifugation at 5000g. The supernatant was collected and mixed with ice‐cold acetone (2.0 mL) at −20 °C for 1 h to precipitate proteins. The mixture was centrifugated at 21 000 g for 45 min; the pellet was collected and dried at rt overnight and resuspended in PBS (50 µL). Protein concentrations were determined with a BCA protein assay kit. Each sample (10 µg proteins/lane) was mixed with the SDS loading buffer (4x stock), boiled at 95 °C, loaded to SDS‐PAGE gel (containing 10% acrylamide), and run at 50 V for 5 h. Proteins in PAGE gel were transferred to a PDVF membrane. The membrane was treated with 2.5% BSA in PBS at rt for 45 min, washed three times with TBST, and incubated with a 1:1000 dilution A488‐Strep solution (5 mL in TBST from 5 µL of 1 mg mL^−1^ stock) at rt for 1 h. The membrane was washed three times with TBST, and imaged (Ex/Em: 492/508 nm) with a Typhoon gel‐imager.

### Studies on the Stability of Functionalized Exosomes

Modified and labelled exosomes recovered from multiple plate wells were combined, washed, concentrated, resuspended in filtered 1xPBS buffer (0.3 mL), frozen, and lyophilized overnight. The powder was resuspended in PBS buffer (0.3 mL) and then applied to DLS. Alternatively, functionalized exosomes were resuspended in PBS or in cell culture medium OptiMEM (0.3 mL) containing 10% FBS, which was followed by incubation at 37 °C in a 5% CO_2_ incubator under sterile conditions for 24 h with continuous shaking. Solution aliquots were collected and applied to DLS. To test the stability of functionalized exosomes under different physiological conditions, the lyophilized exosomes were resuspended in 1xPBS buffer or simulated fluids (0.3 mL). The mixture was incubated at 37 °C in a 5% CO_2_ incubator under sterile conditions with shaking. Aliquots of the solution were collected and submitted to DLS.

### Cell Culture

According to the literature, HeLa, HEK293, MCF7, and MDA‐MB‐231 cells were cultured in DMEM at 37 °C in a 5% CO_2_ incubator maintaining a water‐saturated atmosphere, whereas LS174T, IMR‐90, HepG2, and SK‐MEL‐28 cells in EMEM; T47D in RPMI‐1640; U2OS in McCoy's 5A medium, and A549 in F‐12K supplemented with 10% (v/v) FBS, 100 µg mL^−1^ streptomycin, and 100 U mL^−1^ penicillin.

### Incubation of Exosomes with cells

An optimal number of cells were seeded in chambers of an 8‐well µ‐Slide (for fluorescence imaging) or in the wells of a 24‐well tissue culture plate (for FACS) and cultured in the proper culture media (as mentioned above) for 24 h, allowing cells to grow to ≈70% confluence. The cell culture media was discarded and replenished with OptiMEM media (300 µL) containing exosomes, which was followed by incubation at 37 °C for desired time in a 5% CO_2_ incubator maintaining a water‐saturated atmosphere. Thereafter, cells were fixed with 70% ice‐cold ethanol at −20 °C for 30 min, washed three times with FACS buffer, and subjected to fluorescent imaging or FACS analysis. In some experiments, additional staining may be needed as shown below.

### Fluorescent Imaging of Cells with Internalized Exosomes

After 5 × 10^3^ cells were seeded in each chamber of a µ‐Slide and cultured to reach 50–60% confluence, the media was discarded, and cells were washed three times with OptiMEM. In the meantime, singly or dually fluorescence‐labeled and non‐labeled exosomes collected from 25 plate wells (each containing 1 µg exosomes) were concentrated to 100 µL as described. The exosome solution was transferred into a sterile 1.5 mL‐centrifuge tube, lyophilized, suspended in OptiMEM media (300 µL), and then added to cells in the µ‐Slide. Cells were cultured at 37 °C in a 5% CO_2_ incubator under sterile condition for 10 h and washed with ice‐cold OptiMEM (3 × 500 µL) and PBS (3 × 500 µL). Cells were incubated in 70% ice‐cold ethanol (300 µL) at −20 °C for 30 min. After washing three times with ice‐cold PBS, cells were subjected to additional staining. To stain the nucleus, fixed cells were incubated with 1xPBS (300 µL) containing 0.5 µg mL^−1^ DAPI at rt for 10 min. To stain actin, fixed cells were incubated with PBS containing 0.3 µL phalloidin Ab at rt for 1 h. To stain organelle, fixed cells were treated with PBS containing a fluorophore‐conjugated organelle Ab marker (1 mL in 1:1000 dilution) at rt for 1 h. After staining, cells were thoroughly washed with ice‐cold PBS and imaged using a 40x objective.

### FACS Analysis of Cell‐Internalized Exosomes

$\approx 5\times 10^{4}$) were seeded into each well of a 24‐well plate, cultured in complete DMEM media for 24 h to reach 80–90% confluence, and washed with ice‐cold OptiMEM media (3 × 300 µL). Concentrated singly or dually labeled and non‐labeled exosomes in OptiMEM (300 µL) prepared by the previous protocols were added. After the plate was incubated for the desired time, cells were scraped from each well and put in ice‐cold PBS (1 mL). The cells were pelleted and consecutively washed with ice‐cold glycine buffer (3 × 500 µL), PBS (3 × 500 µL), and FACS buffer (3 × 500 µL). Cells were fixed with 70% ethanol as mentioned above, washed with ice‐cold FACS buffer (3x), and pelleted. Finally, cells were resuspended in FACS buffer (300 µL) for FACS using the 488‐excitation laser. In each experiment, ≈10 000 events were collected, and the FSC/SSC and fluorescent channel voltage settings were consistent for all experiments.

### Fluorescent Imaging of Internalized Exosomes in Live Cells with Cell Surface Proteins Labeled

$\approx 1\times 10^{3}$) were seeded into each chamber of a µ‐Slide and cultured to reach ≈50% confluence. The culture medium was discarded. The cells were washed three times with OptiMEM media and then incubated with 250 µm 488‐NHS ester (1 µL, 0.1 m stock solution in 1:1 DMSO:water) in OptiMEM (200 µL) at 37 °C in a 5% CO_2_ incubator under sterile conditions for 1 h. The cells were washed with OptiMEM and PBS as previously described. Next, the cells were incubated with fluorescence‐labeled or non‐labeled exosomes (reconstituted in 300 µL of OptiMEM medium) at 37 °C in a 5% CO_2_ incubator under sterile conditions for 10 h. The exosome‐containing culture medium was discarded, and the cells were washed several times with fresh OptiMEM (preheated to 37 °C). The cells were replenished with OptiMEM (300 µL) and imaged within 30 mins using a microscope.

### Cellular Uptake of Exosomes in the Presence of Endocytosis Inhibitors

Cells ($\approx 1\times 10^{6}$) were seeded in each well of a 24‐well plate and cultured in the proper media for 24 h until reaching ≈90% confluence. The culture media was replaced with OptiMEM containing 0.1% dimethyl sulfoxide (DMSO) and an endocytosis inhibitor (the concentration of each endocytosis inhibitor is listed in Table S1, Supporting Information) or PBS, followed by incubation at rt for 4 h. After washing with ice‐cold OptiMEM, the concentrated exosomes in OptiMEM (300 µL) were added. The plate was incubated for 10 h, followed by washing with ice‐cold glycine buffer (3 × 500 µL), PBS (3 × 500 µL), and FACS buffer (3 × 500 µL). Finally, cells were fixed with 70% ethanol, washed, pelleted, and resuspended in FACS buffer for FACS analysis as described previously.

### Statistical Analysis

Statistical analysis of data was performed using the GraphPad Prism 9.0 software. Results are presented as means ± standard deviations and were compared using the two‐tailed Student's *t*‐test. *p* < 0.05 is considered as statistically significant.

## Conflict of Interest

The authors declare no conflict of interest.

## Supporting information



Supporting Information

## Data Availability

The data that support the findings of this study are available in the supplementary material of this article.
