# Supplementary material for: A New Strategy to Functionalize Exosomes via Enzymatic Engineering of Surface Glycans and its Application to Profile Exosomal Glycans and Endocytosis
Source: Adv Sci (Weinh). 2025 Mar 19;12(18):2415942. doi: 10.1002/advs.202415942 (PMC12079434; doi:10.1002/advs.202415942)
Supplement: Supplementary file 1 — Supporting Information [file ADVS-12-2415942-s001.pdf]

ADVANCED SCIENCE
Open Access

Supporting Information

for *Adv. Sci.*, DOI 10.1002/advs.202415942

A New Strategy to Functionalize Exosomes via Enzymatic Engineering of Surface Glycans and its Application to Profile Exosomal Glycans and Endocytosis

*Sayan Kundu, Jiatong Guo, Md. Shamiul Islam, Rajendra Rohokale, Mohit Jaiswal and Zhongwu Guo**

Supporting Information

For

A new strategy to functionalize exosomes via enzymatic engineering of surface glycans and its application to profile exosomal glycans and endocytosis

Sayan Kundu,[a]† Jiatong Guo,[a]† Md. Shamiul Islam,[a] Rajendra Rohokale,[a] Mohit Jaiswal,[a] and Zhongwu Guo[a,b]*

[a] Department of Chemistry, University of Florida, Gainesville, Florida 32611, United States

[b] UF Health Cancer Centre, University of Florida, Gainesville, Florida 32611, United States

* Corresponding author: zguo@chem.ufl.edu

† These authors contributed equally to the current work.

Table of contents

Scheme S1: Synthesis of CMP-Neu5Ac9N3 by a reported procedure SI-3

Figure S1: Exosomal surface protein-mediated exosome attachment to the plate SI-3

Figure S2: A5 attachment to the plate and the influences of exosome concentration SI-3

Figure S3: EDTA-mediated release of exosomes from the plate SI-4

Figure S4: DSL profiles of the exosomes SI-4

Figure S5: FACS results of latex beads loaded with exosomes SI-5

Figure S6: Influences of Pd-2,6-ST concentration on EGE of exosomes SI-6

Figure S7: Influences of temperature on EGE of exosomes...SI-6

Figure S8: Influences of enzymatic reaction time on EGE of exosomes.. ....SI-6

Figure S9: Influences of DBCO-biotin concentration on EGE of exosomes................................SI-7

Figure S10: Fluorescence analysis results of EDTA-released exosomes..................................SI-7

Figure S11: DLS profiles of EDTA-released exosomes...SI-8

Figure S12: FACS of biomarkers present on EGE-modified exosomes......................................SI-8

Figure S13: Impacts of PNGase F on glycoengineered exosomes...SI-9

Figure S14: FACS results of functionalized exosomes released from the plate SI-9

Figure S15: Fluorescent micrographs of exosomes...SI-10

Figure S16: Total proteins and EGE-label proteins of functionalized exosomes..........................SI-10

Figure S17: DLS results of lyophilized functionalized exosomes..SI-11

Figure S18: DLS results of functionalized exosomes stored in PBS......................................SI-11

Figure S19: DLS results of functionalized exosomes stored in simulated physiological buffers….SI-12
Figure S20: FACS result of exosomes with labeled lipids, proteins, and glycans ………………..SI-12
Figure S21: Influences of exosome concentration on EGE efficiency and comparison of two exosome detection methods based on EGE and antibodies……………………SI-13
Figure S22: EGE results of exosomes from different cell lines…………………………………...SI-13
Figure S23: Fluorescent micrographs of HeLa cells treated with A488-exosomes………………SI-14
Figure S24: Fluorescent micrographs of HeLa cells treated with A488-exosomes for different time……………..…………………………………………………….....SI-15
Figure S25: FACS results of HeLa cells treated with A488-exosomes for different time…………SI-16
Figure S26: FACS results of HeLa cells treated with varied concentrations of A488-exosomes ...SI-17
Figure S27. Fluorescent micrographs of HeLa cells treated with A488-labeled exosomes and various endocytosis inhibitors …………………………………………………..SI-18
Figure S28. FACS results of HeLa cells incubated with A488-exosomes and various endocytosis inhibitors………………………………………………………………..SI-19
Figure S29-S30: Fluorescence remained in Hela cells labeled with functionalized exosomes upon incubation in exosome-free media…….…………………………………………SI-20
Figure S31: Fluorescent images of Hela cells labeled with functionalized exosomes upon incubation in exosome-free media……………………………………………………SI-22
Figure S32: Fluorescent micrographs of various cells treated with A488-exosomes………………SI-23
Figure S33-S41: Gated one-fluorescence (488) FACS results of HEK293, MDA-MB-231, MCF7, T47D, LS174T, A549, U2OS, SK-MEL-28, and HepG2 cells incubated with A488-exosomes and various endocytosis inhibitors……………………………..SI-24
Figure S42-S44: Fluorescent images of HeLa cells treated with dually labeled exosomes…………SI-29
Figure S45: Fluorescent images of HeLa cells cultured with A488-glycan-labeled exosomes and A647-lebeled transferrin….………….…………………………………SI-32
Figure S46: Fluorescent images of live HeLa cells containing A488-labeled surface proteins treated with A555-glycan-labeled exosomes……………………………..………SI-33
Figure S47: Fluorescent images of HeLa cells incubated with A488-labeled exosomes after lyophilization and long-term storage in PBS buffer……………………………SI-34
Table S1: Endocytosis inhibitors with concentration reported and used in this study…………….SI-35
Table S2. Proteins and antibodies used in this work……………………………………………SI-35
Figure S48-53: NMR and MS spectra of Neu5Ac9N_3 and CMP-Neu5Ac9N_3…………………………SI-36
Table S3. Catalog numbers of purchased exosomes ………………………………………………..SI-39
The certificate of analysis for the purchased human serum exosomes……………………………..SI-39

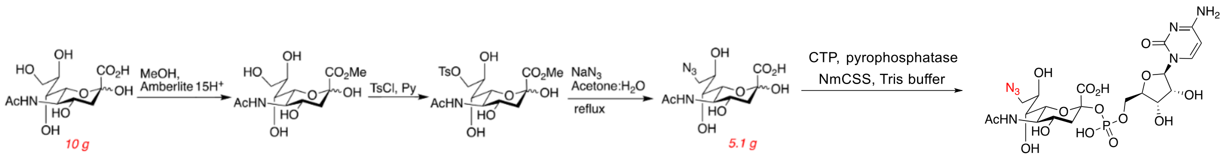

Scheme S1: Synthesis of CMP-Neu5Ac9N_3 by a reported procedure.

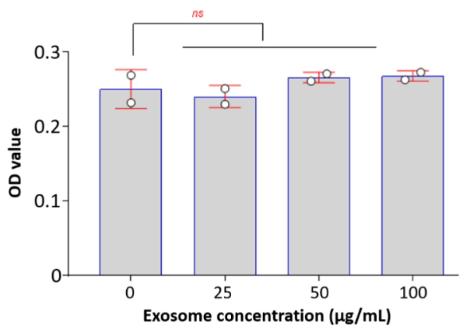

Figure S1: Quantification of exosomal surface protein-mediated exosome attachment to the 96-well plate. After a plate was incubated with various concentrations (0, 25, 50, and 100 μg/mL) of HS exosomes following the protocol described in the experimental section, a biotinylated anti-CD81/63/9 antibody cocktail was added to each well, followed by the addition of streptavidin-AP and PNPP. After incubation, the plate was subjected to colorimetric measurement of the OD values using a microplate reader. Data are shown as the average of two parallel experiments ± SD. ns $p > 0.05$ for the two compared groups.

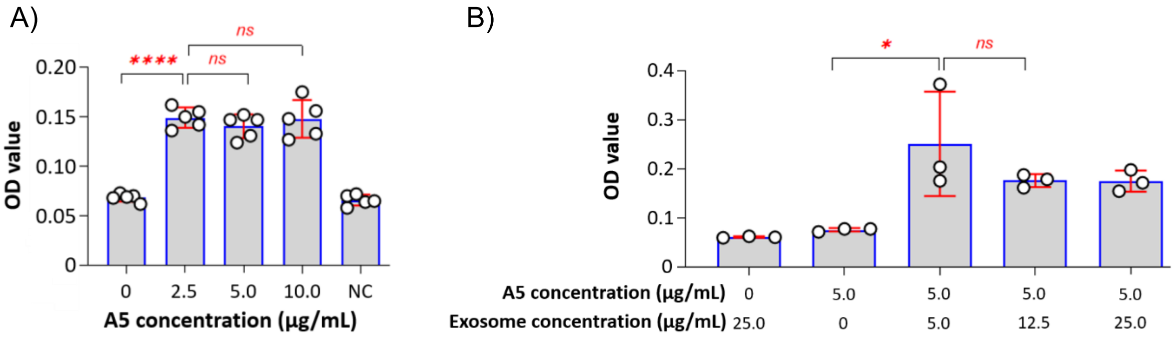

Figure S2: (A) Quantification of A5 protein attachment to a 96-well plate. After a plate was incubated with 0, 2.5, 5.0, and 10.0 μg/mL of A5 following the protocol described in the experimental section, a biotinylated anti-A5 Ab was added to each well, followed by addition of Strep-AP and PNPP. After incubation, the plate was subjected to colorimetric measurement of the OD values using a microplate reader. NC as another negative control: The plate was treated by the same protocol using 10.0 μg/mL of A5 and a biotinylated isotype Ab, instead of a biotinylated anti-A5 Ab for detection. (B) The influences of HS exosome concentration on A5-meidated exosome attachment to the 96 well plate. After incubation with A5 (or without A5) and then with 0, 5.0, 12.5, and 25.0 μg/mL of HS exosomes, a biotinylated anti-CD81/63/9 antibody cocktail was added to each well, and the exosomes on the plate were quantified using the Strep-AP/PNPP assay. Data are shown as the average of five or three parallel experiments ± SD. Level of statistical significance: ns $p > 0.05$, * $p < 0.05$, **** $p < 0.0001$ for the two compared groups.

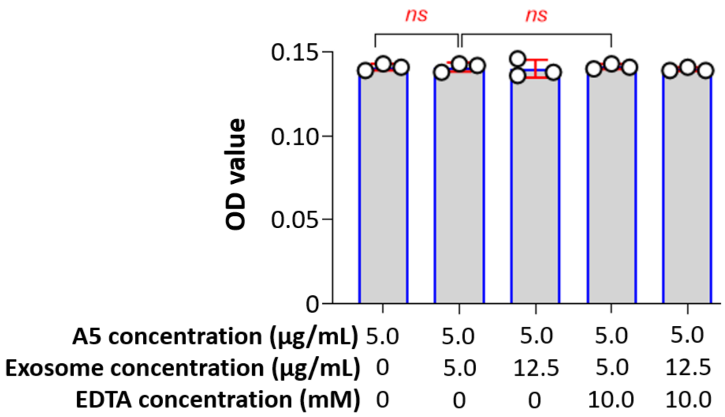

Figure S3: EDTA-mediated release of exosomes attached to plates with A5. After incubation with A5 and then with 0, 5.0, and 12.5 µg/mL of HS exosomes, the plate was treated with 0 or 10 mM EDTA. The exosomes remaining on the plate were quantified using the biotinylated anti-CD81/63/9 antibody cocktail and Strep-AP/PNPP assay. Data are presented as the average of three parallel experiments ± SD. Level of statistical significance: ns $p > 0.05$ for the two compared groups.

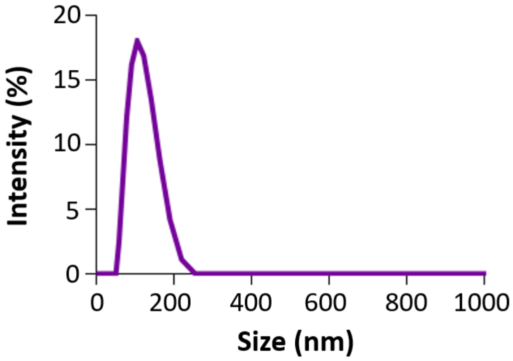

Figure S4: Dynamic light scattering (DLS) profile of the exosomes in the supernatants of EDTA-treated plate wells.

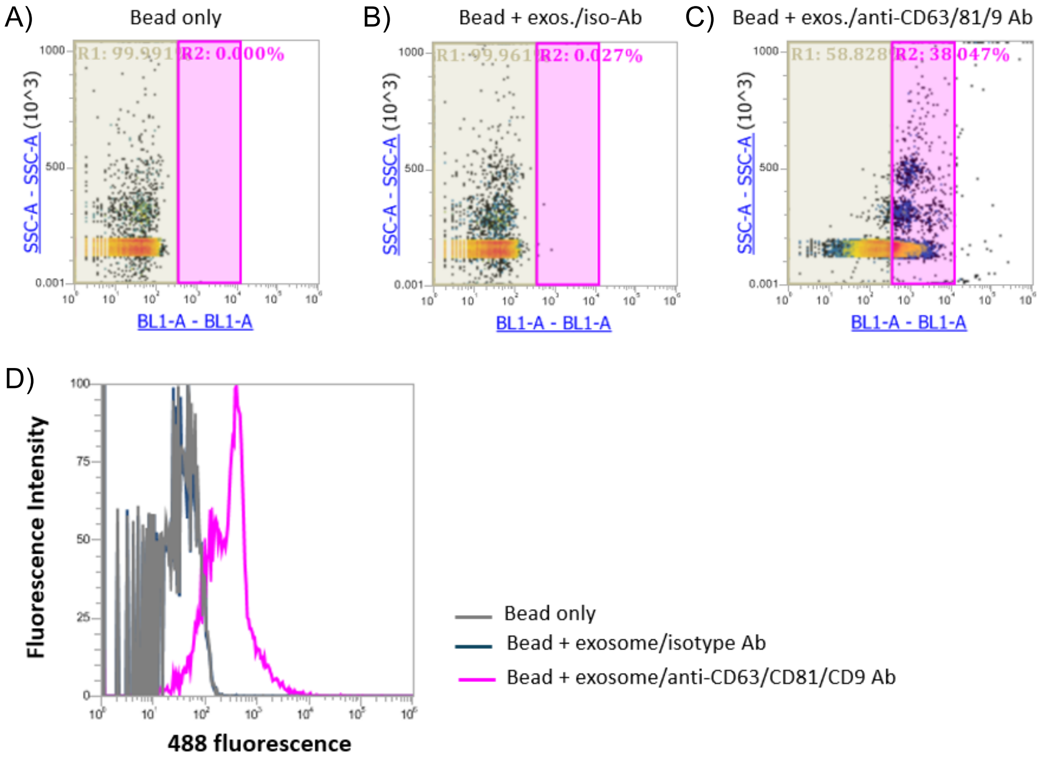

Figure S5: (A-C) FACS results of single-color (488) fluorescence density plots of latex beads only (A), bead-absorbed exosomes from the EDTA supernatants of the plate wells after incubation with A488-isotype Ab (B) or with an A488-anti-CD63/81/9-Ab cocktail (C). X-axis: 488 fluorescence; Y-axis: side scattering (SSC) value. The gate was selected with respect to the beads without exosome treatment. (D) Single parameter 488 fluorescence FACS histograms of the beads alone (grey), beads treated with exosome and 488-isotype Ab (blue), and beads treated with exosome and 488-anti-CD63/81/9-Ab cocktail (pink).

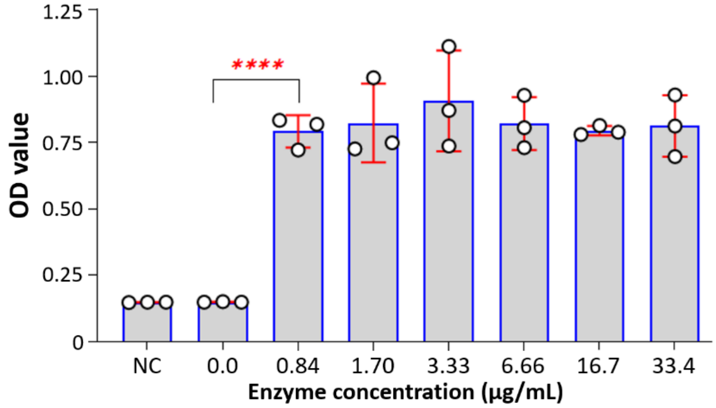

Figure S6: On-plate modification of A5-captured HS exosomes (5 μg/mL) via EGE of exosomal surface glycans using CMP-Neu5Ac9N$_3$ (1.67 mg/mL) and different concentrations of Pd-2,6-ST (0-33.4 μg/mL), followed by reaction with biotin-DBCO and then Strep-AP/PNPP colorimetric assay. Data are shown as the average of three parallel experiments ± SD. NC: the exosomes were treated with PBS replacing CMP-Neu5Ac9N$_3$. Level of statistical significance: ****p < 0.0001 for the two compared groups.

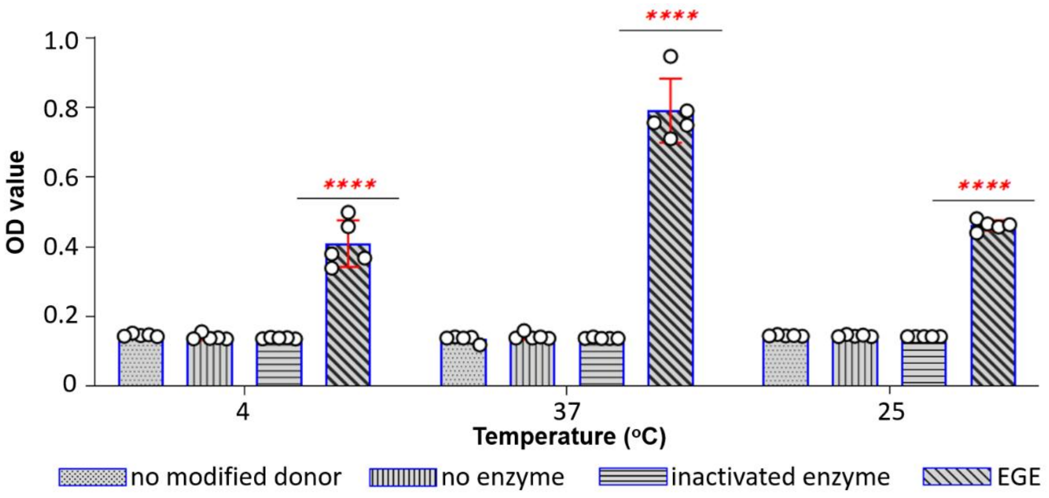

Figure S7: Influences of temperature (4, 37, and 25 ºC) on the on-plate modification of A5-captured HS exosomes (5 µg/mL) through EGE of exosomal surface glycans using CMP-Neu5Ac9N$_3$ (1.67 mg/mL) and Pd-2,6-ST (3.3 µg/mL), followed by reaction with biotin-DBCO and then Strep-AP/PNPP colorimetric assay. NCs: the exosomes were treated with PBS replacing CMP-Neu5Ac9N$_3$, with PBS replacing Pd-2,6-ST, and with inactivated Pd-2,6-ST replacing active Pd-2,6-ST. Data are shown as the average of five parallel experiments ± SD. Level of statistical significance: ****p < 0.0001 compared to other groups at the same temperature.

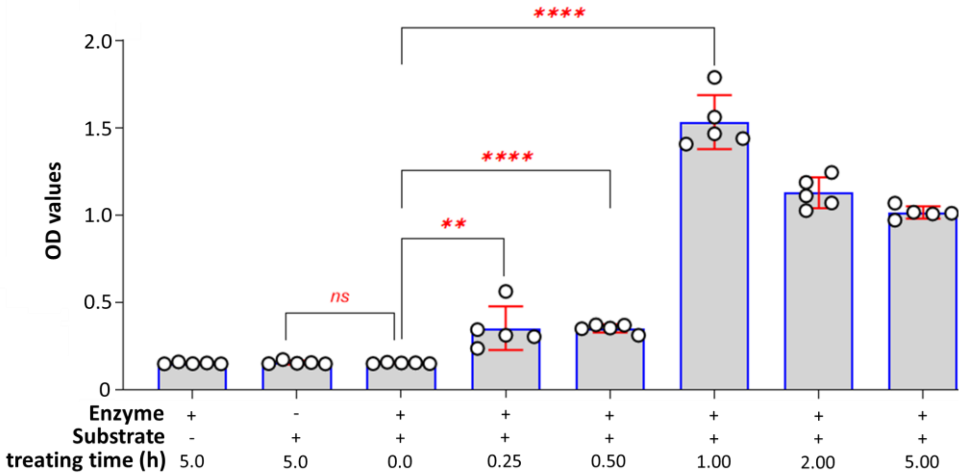

Figure S8: Influences of enzymatic reaction time (0-5 h) on the on-plate modification of A5-captured HS exosomes (5 µg/mL) through EGE of exosomal surface glycans using CMP-Neu5Ac9N$_3$ (0 or 1.67 mg/mL) and Pd-2,6-ST (0 or 3.3 µg/mL), followed by reaction with biotin-DBCO and then Strep-AP/PNPP colorimetric assay. Data are shown as the average of five parallel experiments ± SD. Level of statistical significance: ns p > 0.5, **p < 0.01 and ****p < 0.0001 for the two compared groups.

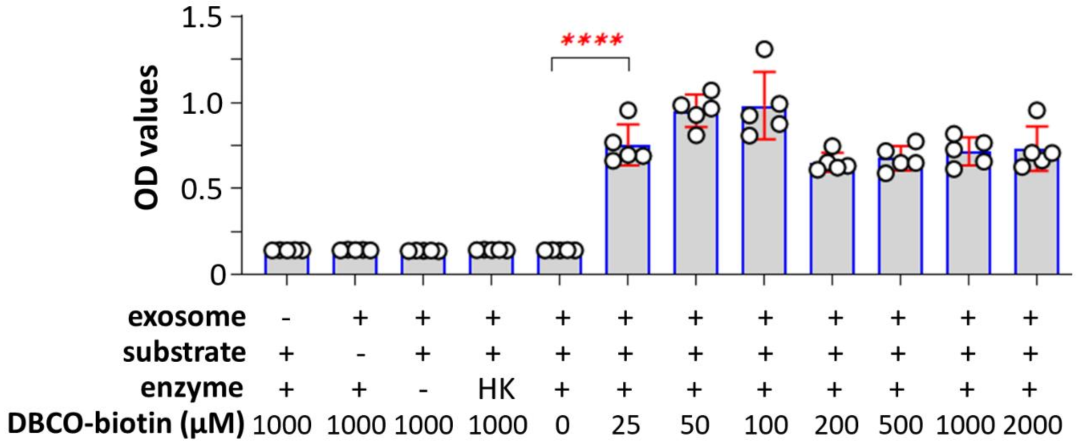

Figure S9: Influences of DBCO-biotin concentration (0-2000 μM) on the on-plate functionalization of glycoengineered HS exosomes (5 μg/mL) using CMP-Neu5Ac9N$_3$ (0 or 1.67 mg/mL) and Pd-2,6-ST (0 or 3.3 μg/mL), followed by reaction with different concentrations of biotin-DBCO and then Strep-AP/PNPP colorimetric assay. HK: heat-killed or inactivated Pd-2,6-ST. Data are shown as the average of five parallel experiments ± SD. Level of statistical significance: ****$p < 0.0001$ for the two compared groups.

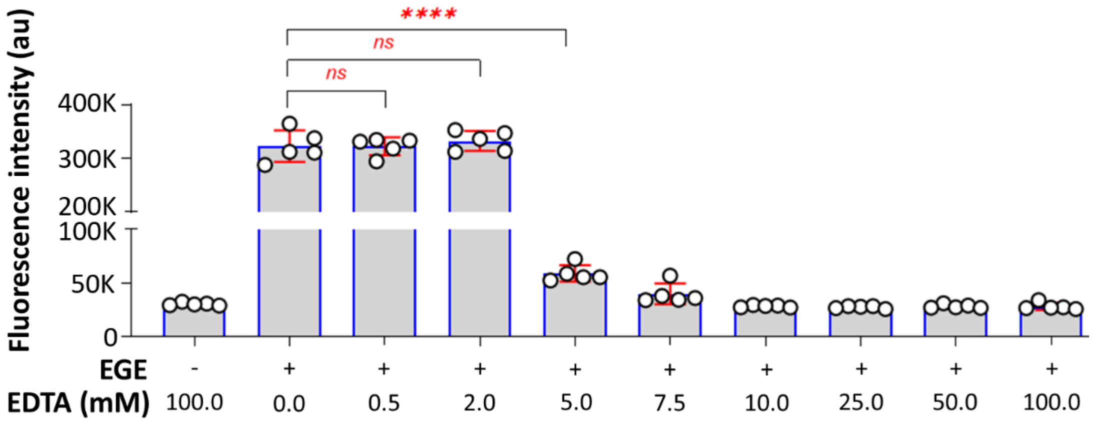

Figure S10: Fluorescence analysis of EDTA-released exosomes from the plate after EGE-based modification. After HS exosomes (5 μg/mL) were glycoengineered with CMP-Neu5Ac9N$_3$ (1.67 mg/mL) and Pd-2,6-ST (3.3 μg/mL), biotin-DBCO (100 μM) was added, which was followed by Strep-A488 (5 μg/mL). Thereafter, the plate was treated with different concentrations of EDTA, and after washing, the fluorescence intensity of each well was recorded. Data are shown as the average of five parallel experiments ± SD. Level of statistical significance: ns $p > 0.05$, ****$p < 0.0001$ for the two compared groups.

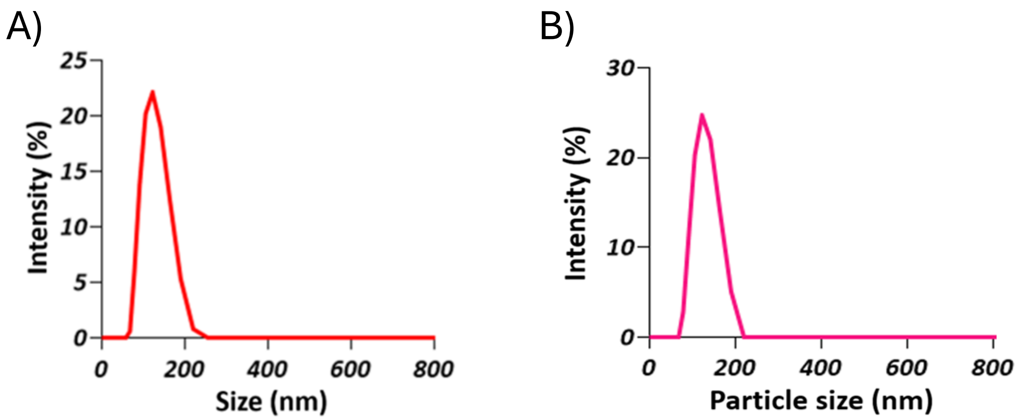

Figure S11: DLS profiles of Biotin-modified (A) and A488-modified (B) exosomes in the EDTA supernatants. After HS exosomes were enzymatically glycoengineered on-plate, modified with DBCO-biotin (A) or modified with DBCO-biotin and then with Strep-A488 (B), the plates were treated with EDTA (10 mM), and the supernatants were collected, condensed, and subjected to DLS analysis.

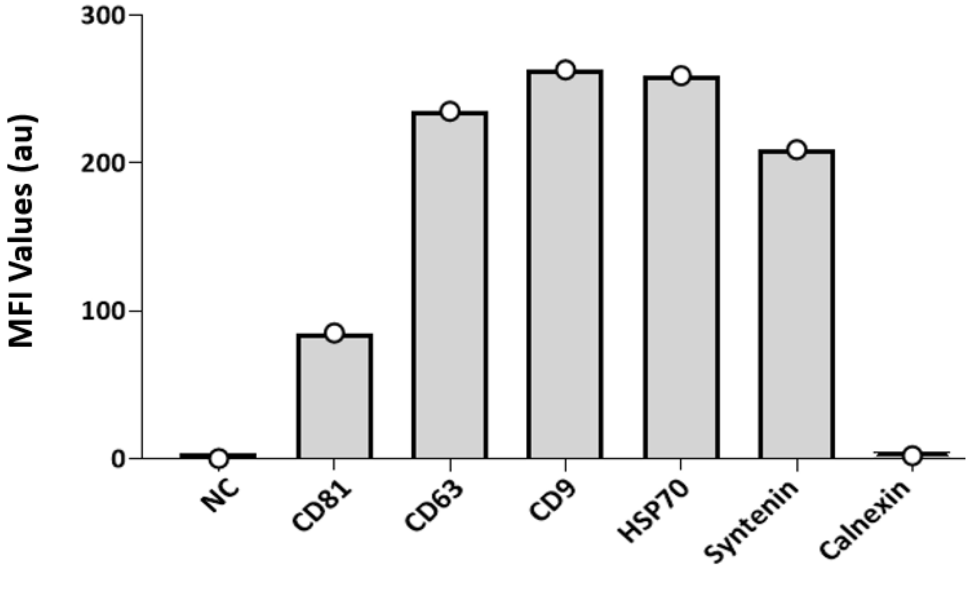

Figure S12: FACS results to determine CD81, CD63, CD9, HSP70, and Syntenin biomarkers (one experiment only) present on the EGE-modified HS exosome surface, using A488-conjugated anti-biomarker antibodies, having exosomes loaded to microbeads and PBS, with Calnexin as negative controls.

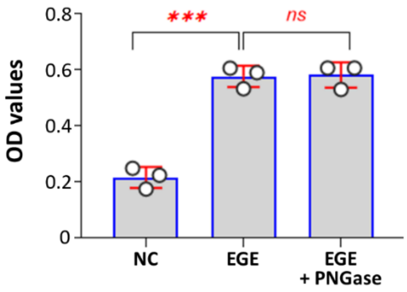

Figure S13: Results of on-plate peptide N-glycosidase (PNGase) F treatments of glycoengineered exosomes on their OD values. After HS exosomes (5 µg/mL) attached to the A5-coated (5.0 µg/mL per well) plate, they were subjected to chemoenzymatic reaction with CMP-Neu5Ac9N$_3$ (1.67 mg/mL) and Pd-2,6-ST (3.33 µg/mL). Then, the plate was treated with PNGase F, followed by functionalization with biotin-DBCO (100 µM) and then detection using the AP-Strep/PNPP colorimetric assay, which is compared to similarly glycoengineered and functionalized exosomes without PNGase F treatment. Data are shown as the average of three parallel experiments ± SD. NC is exosomes similarly treated without EGE. Level of statistical significance: ns $p > 0.05$, ***$p < 0.001$ for the two compared groups.

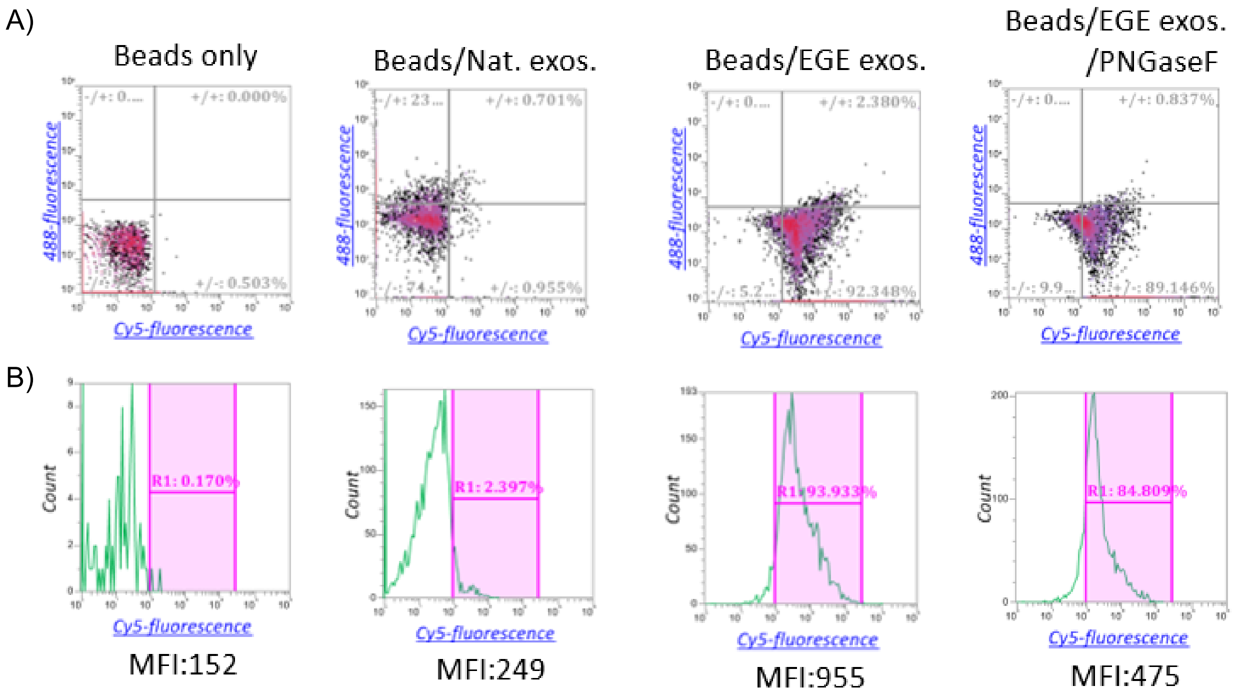

Figure S14: (A) Dual color-flow cytometry density plots and (B) the corresponding Cy5-fluorescence histograms of glycoengineered and functionalized exosomes released from the plate using EDTA after attachment to aldehyde/sulfate latex beads. After HS exosomes (5 µg/mL) attached to A5-coated (5.0 µg/mL per well) plates, they were subjected to EGE using CMP-Neu5Ac9N$_3$ (1.67 mg/mL) and Pd-2,6-ST (3.33 µg/mL). The plates were then treated with PBS (EGE exos.) or PNGase F (EGE exos./PNGaseF), followed by functionalization with biotin-DBCO (100 µM) and then with Cy5-Strep. The exosomes were released from the plate with EDTA (10 mM), collected, condensed, and incubated with the beads. Subsequently, the beads were incubated with A488-conjugated anti-CD81/63/9 Ab cocktail, and finally analyzed using a flow cytometer. The NCs include beads without exosome treatment (beads only) and beads treated with native bead without EGE (beads/nat. exos.) X axis: Cy5-fluorescence intensity showing functionalized exosomes; Y axis: 488-fluorescence intensity showing the labeled native CD81, CD63, and CD9 proteins on the exosomes with Ab for (A) and the counts of events for (B), as well as the MFI values at the bottom for (B).

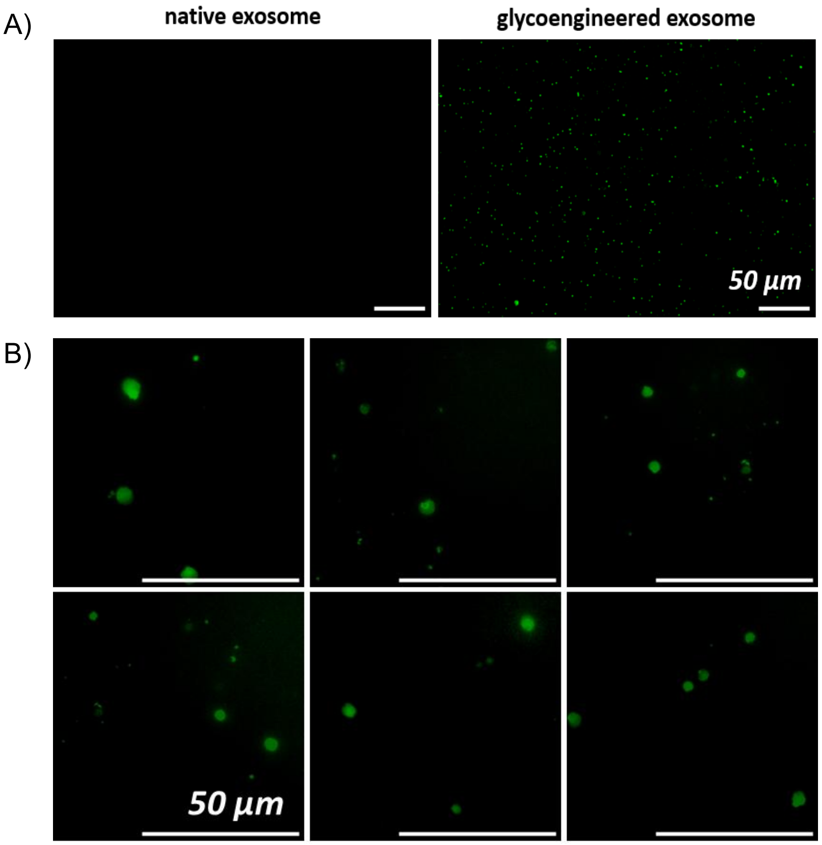

Figure S15: Fluorescent micrographs of (A) native exosomes (left) and functionalized exosomes (right) using Pd-2,6-ST/CMP-Neu5Ac9N$_3$ and DBCO-biotin/A488-streptavidin in the supernatant after EDTA treatment with 10x objective lens, having exosomes attached to imaging glass coverslips with anti-CD63/81/9 Ab, and (B) functionalized exosomes in the EDTA supernatant with 60x oil objective lens (samples from six independent experiments). Ex/Em wavelength: 488/510 nm; scale bars are 50 µm.

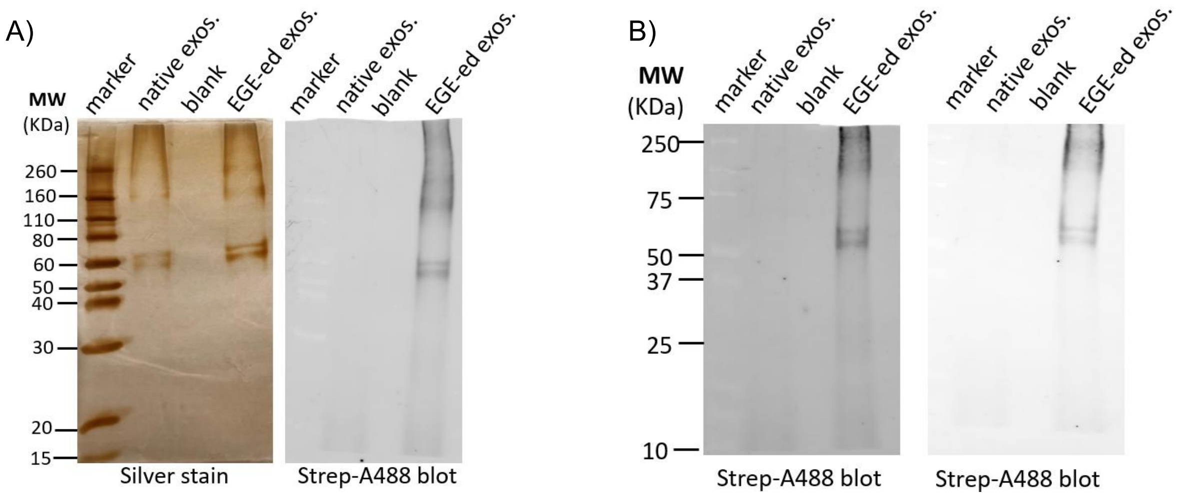

Figure S16: (A) Silver staining of all proteins (left panel) and Western blot of biotinylated proteins using Strap-A488 to show biotinylated proteins derived from native and glycoengineered/biotinylated exosomes, respectively. (B) Strep-A488 Western blot images of biotinylated proteins from native and glycoengineered exosomes: results of two additional independent experiments.

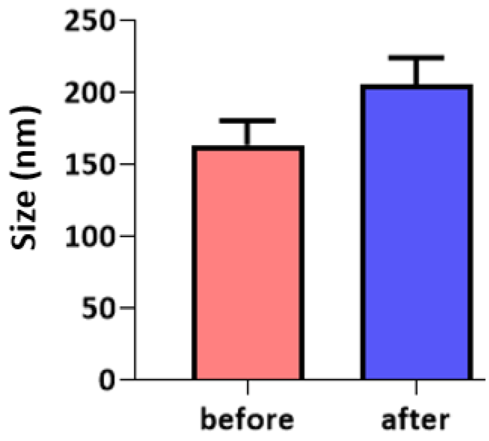

Figure S17: DLS results showing the average hydrodynamic diameters (triplicate experiments) of EGE-modified A488-labeled HS exosomes before (red) and after (blue) lyophilization. Their difference is not statistically significant ($p > 0.05$).

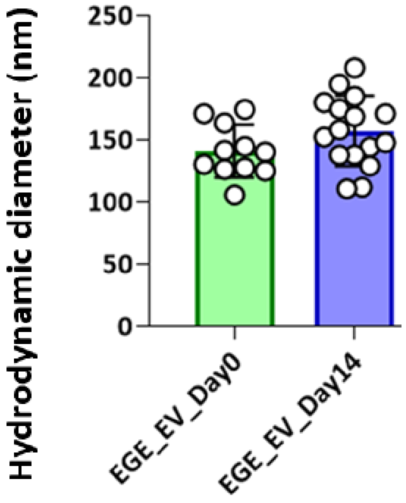

Figure S18: DLS results showing the average hydrodynamic diameter (multiple experiments) of EGE-modified A488-labeled HS exosomes freshly prepared (green, Day0) or after storage in PBS buffer at 37 °C in 5% CO_2 for 14 d (blue, Day14). Their difference is not statistically significant ($p > 0.05$).

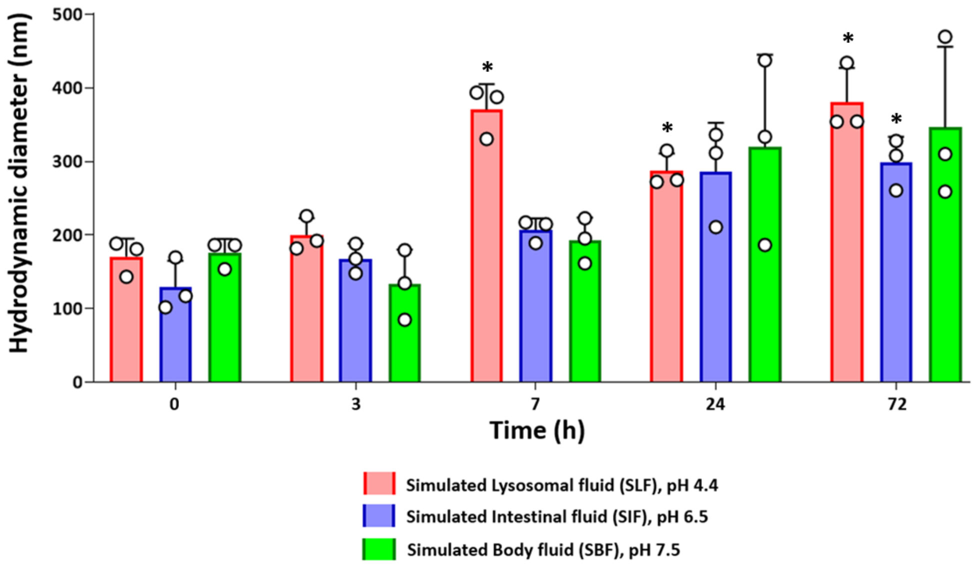

Figure S19: DLS results showing the average hydrodynamic diameters (triplicate experiments) of EGE-modified A488-labeled HS exosomes treated with various simulated physiological buffers, including lysosomal, intestinal, and body fluids, for different periods of time. Data are shown as the average of three parallel experiments ± SD. * Difference from the data of the same group at 0 h is statistically significant ($p < 0.05$).

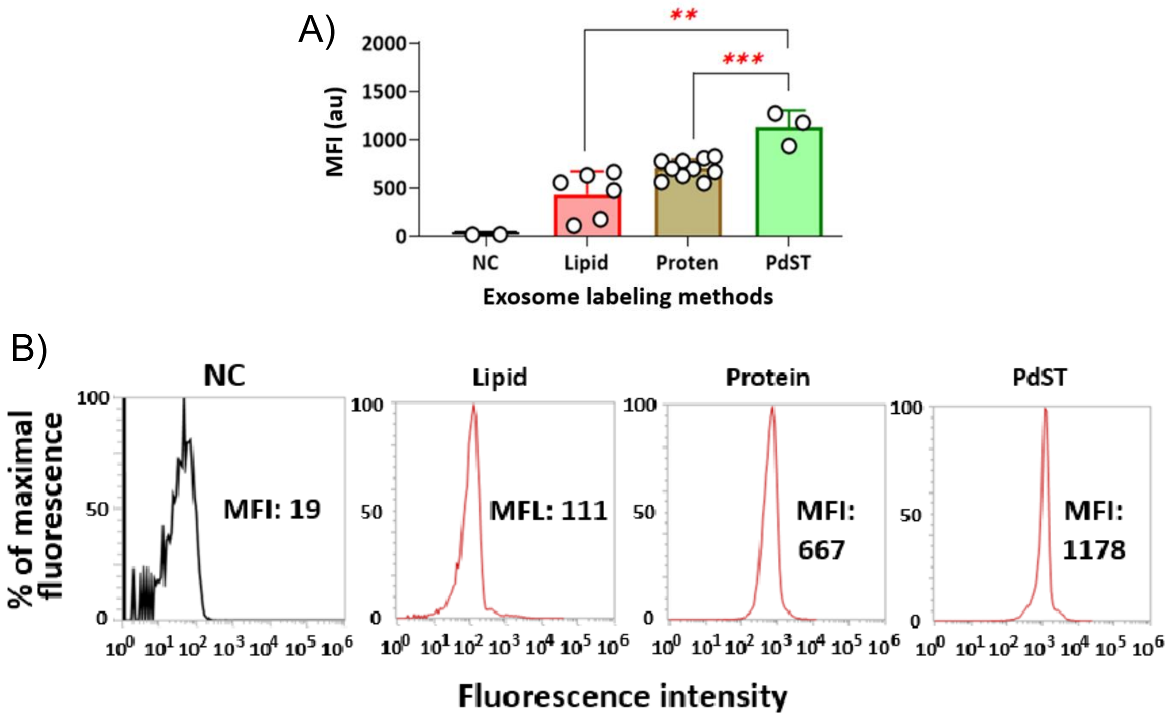

Figure S20: (A) FACS results of different labeling methods for exosomes: using PKH67 dye to label membrane lipids, A488-NHS to label proteins, and Pd-2,6-ST to modify glycans followed by labeling with A488-DBCO. FACS was performed using exosome-loaded beads with the 488-channel. The negative control (NC) was native exosome without labels. (B) Representative FACS histograms showing the fluorescence intensity profiles of NC and exosomes with lipid, protein, and glycan labels. Level of statistical significance: $^{**}p < 0.01$ and $^{***}p < 0.001$.

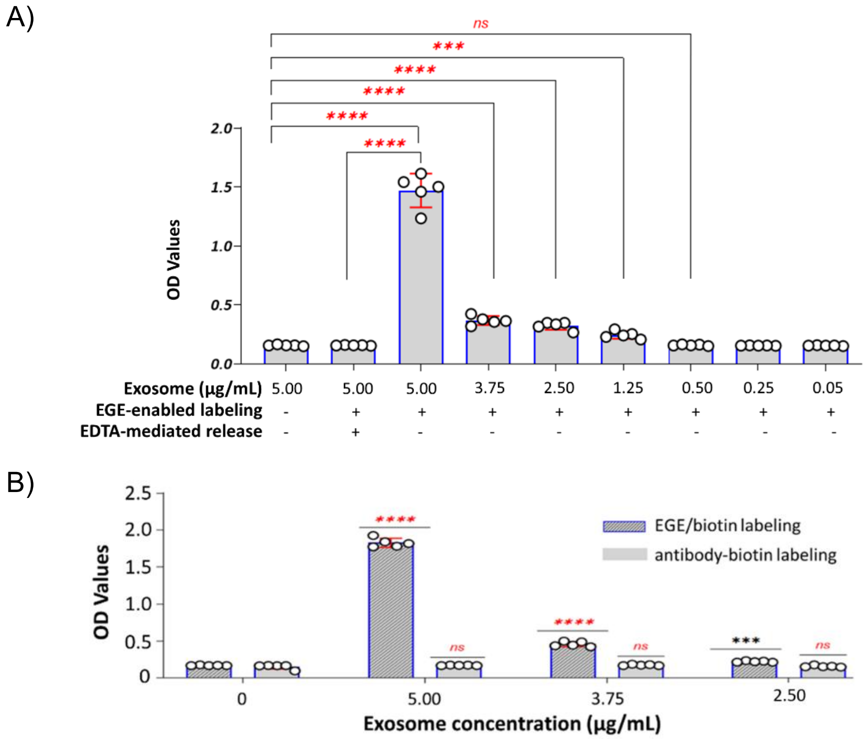

Figure 21: (A) AP-Strap/PNPP-based quantification of the efficiency of exosome EGE using different concentrations of HS exosomes with native exosomes without EGE and EDTA-treated plates as negative controls. (B) Comparison of two exosome detection methods using a biotinylated anti-antibody CD81/63/9 Ab cocktail (antibody-biotin labeling) and using EGE and DBCO-biotin (EGE-biotin labeling). Data are shown as the average of five parallel experiments ± SD. Level of statistical significance: ns $p > 0.05$, ***$p < 0.001$, and ****$p < 0.0001$ for the two compared groups.

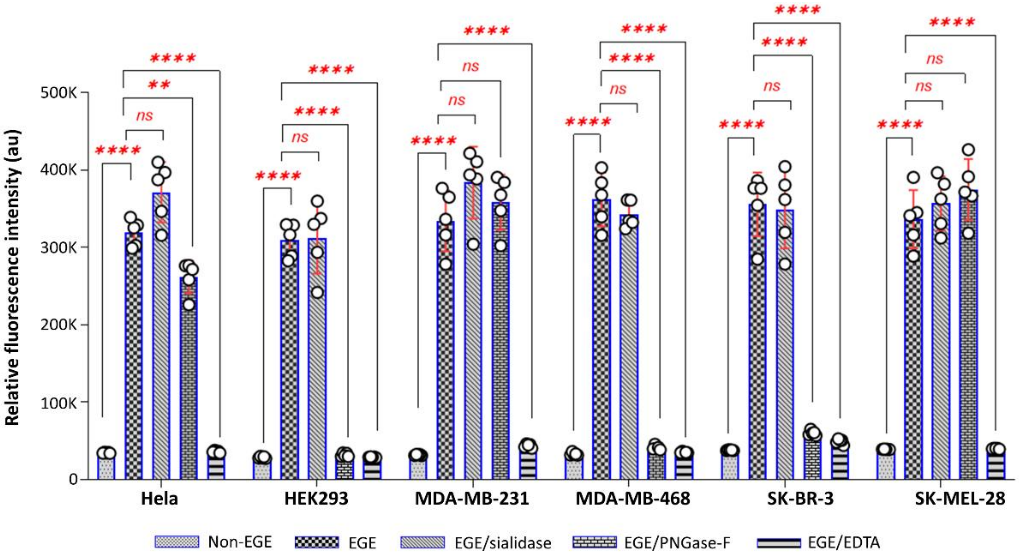

Figure S22: EGE results of commercial exosomes derived from different cell lines, including HeLA, HEK 293, MDA-MB-231, MDA-MB-468, SK-BR-3, and SK-MEL-28, as well as the influence of sialidase treatment (EGE/sialidase) that is supposed to not have a significant impact, PNGase F treatment (EGE/PNGase F), or EDTA treatment (EGE/EDTA), as compared to native exosomes (non-EGE), after on-plate functionalization with DBCO-biotin and Strep-A488 of five independent experiments. Data are presented as the average of five parallel experiments ± SD. Level of statistical significance: ns $p > 0.05$, ***$p < 0.001$, and ****$p < 0.0001$ for the two compared groups.

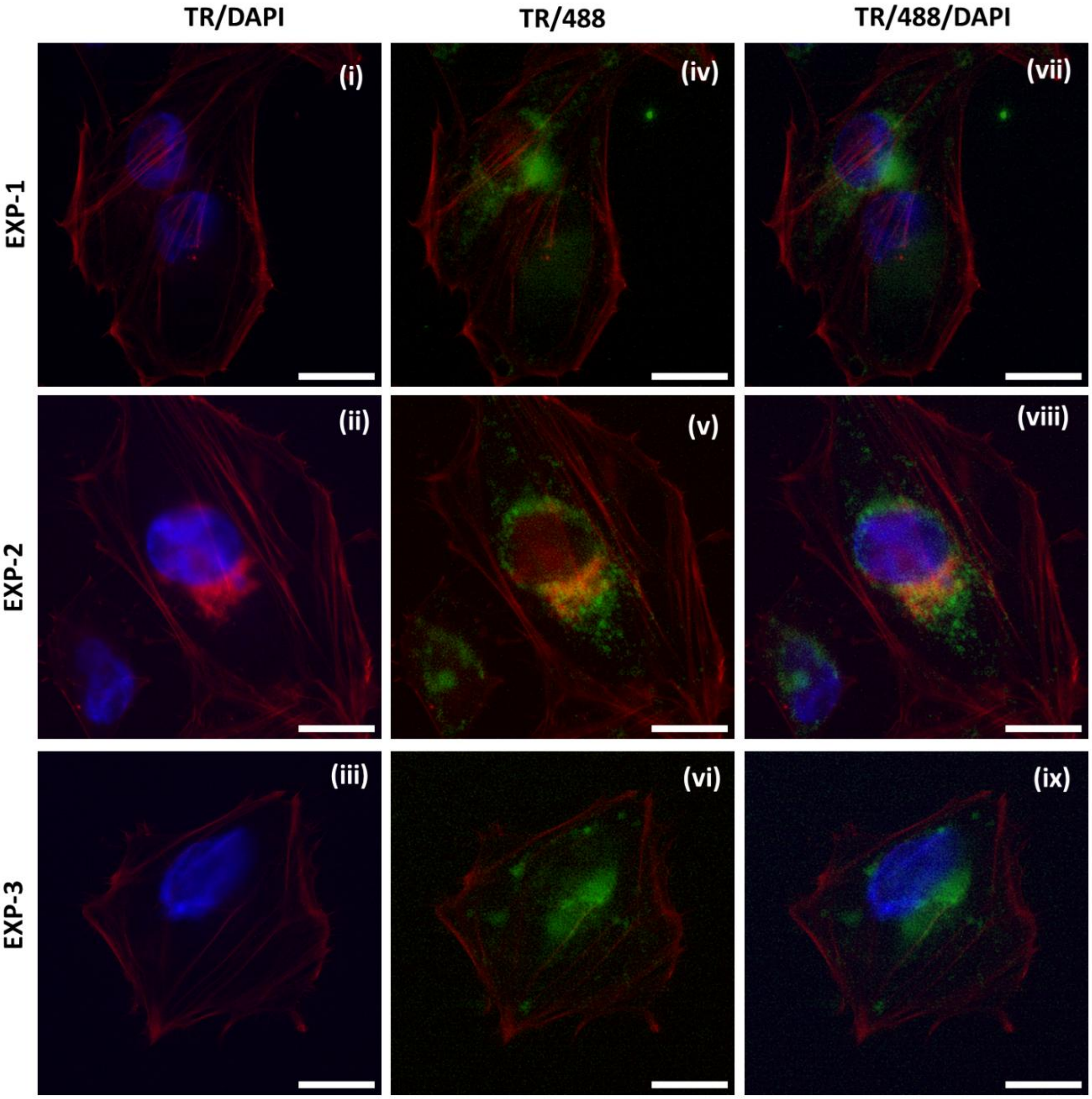

Figure S23: Fluorescent micrographs from three independent experiments to repeat the uptake of A488-labeled exosomes by HeLa cells after incubation for 10 h. The bar: 20 nm.

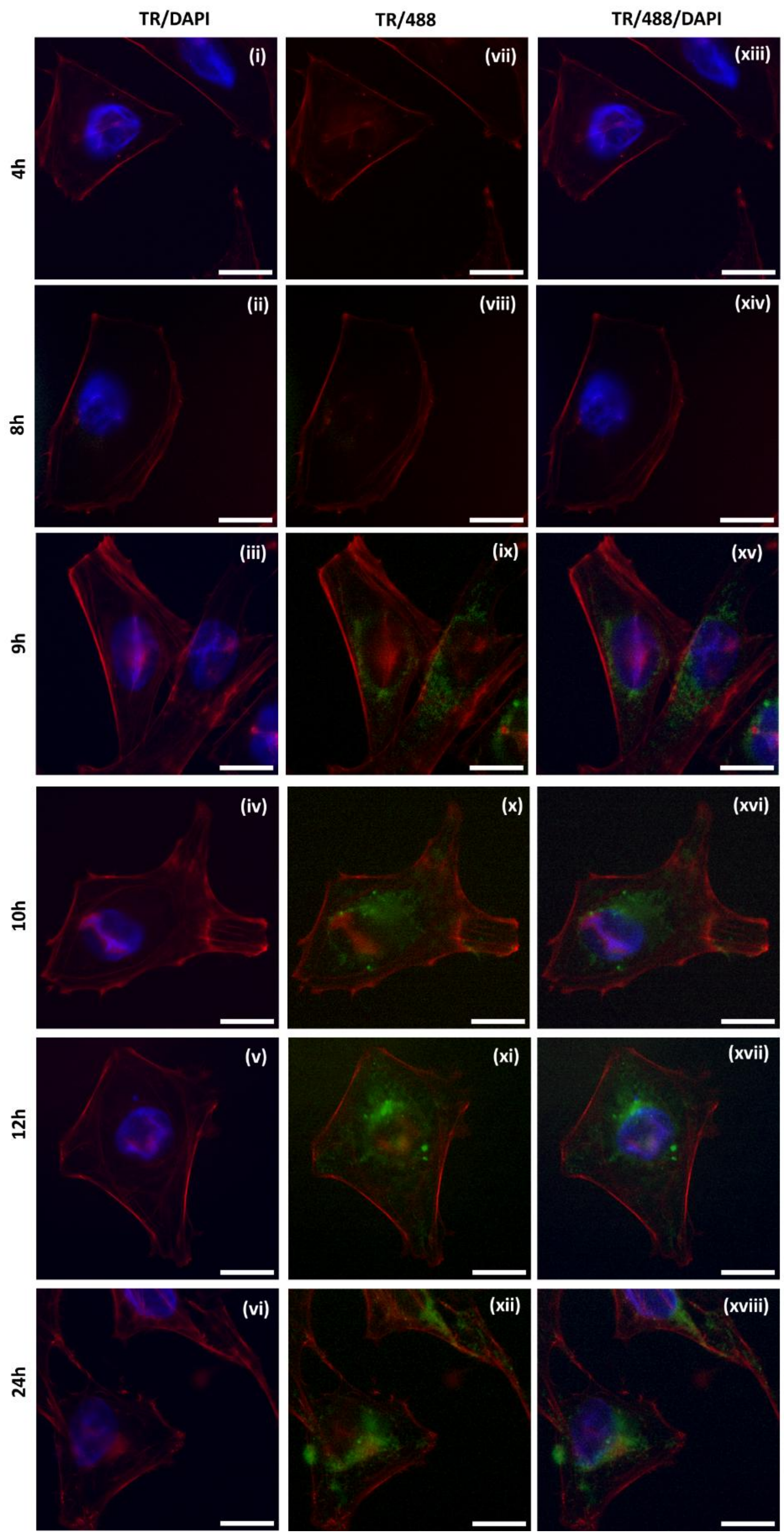

Figure S24: Fluorescent micrograph of HeLa cells treated with A488-fluorescent exosomes for different periods of time from 4 h to 24 h. The bar: 20 nm.

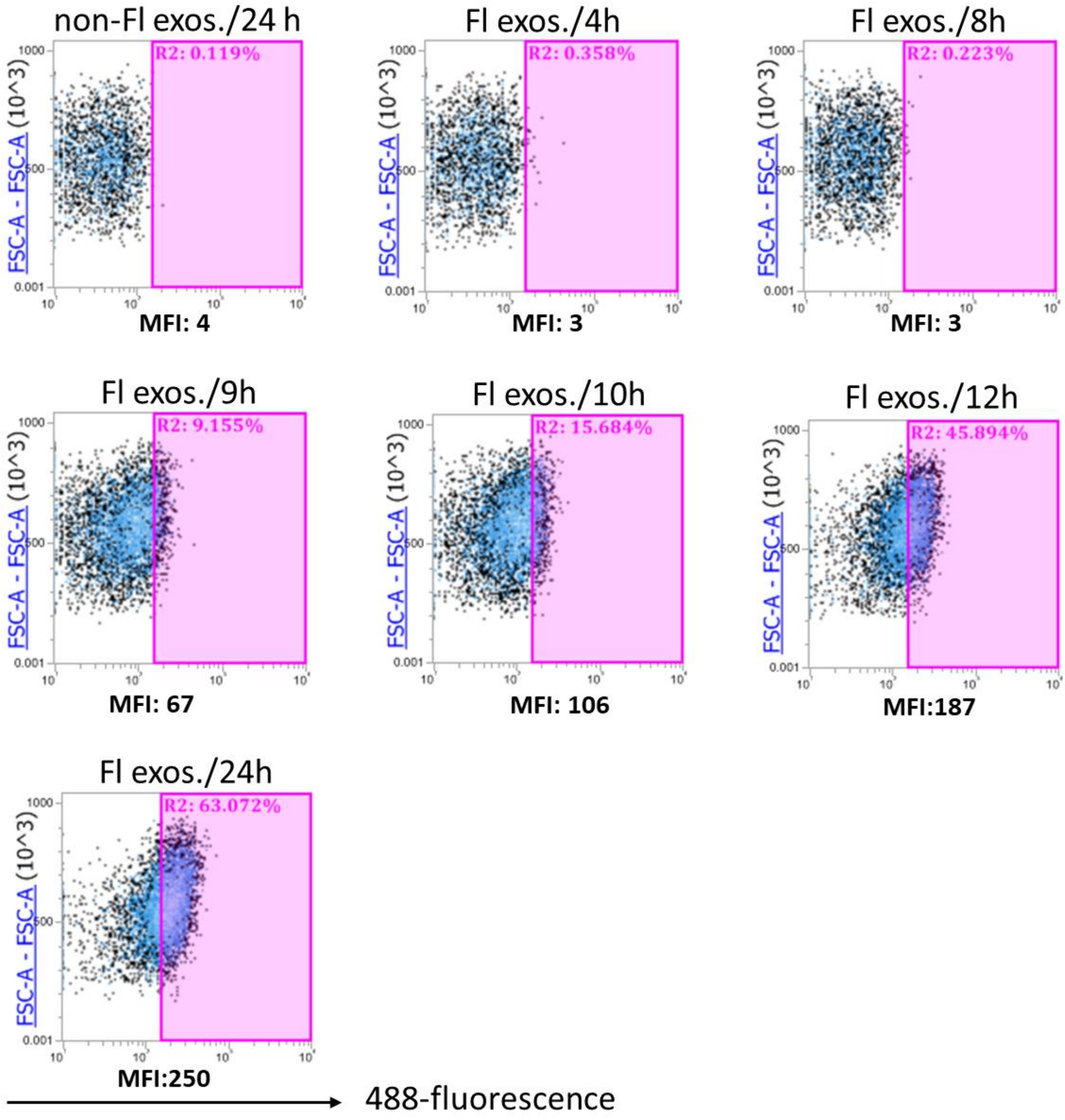

Figure S25: The representative one fluorescence (488) FACS density plots of HeLa cells incubated with A488-labeled exosomes for different periods of time (4-24 h), with corresponding MFI value, along the 488-fluorescence channel, stated at the bottom of each plot. HeLa cells incubated with native nonfluorescent exosomes (non-fl exos./24h) were used as the NC. The 488-positive gate was set up with respect to the NC; the percentage of 488-positive cell population is given in each plot.

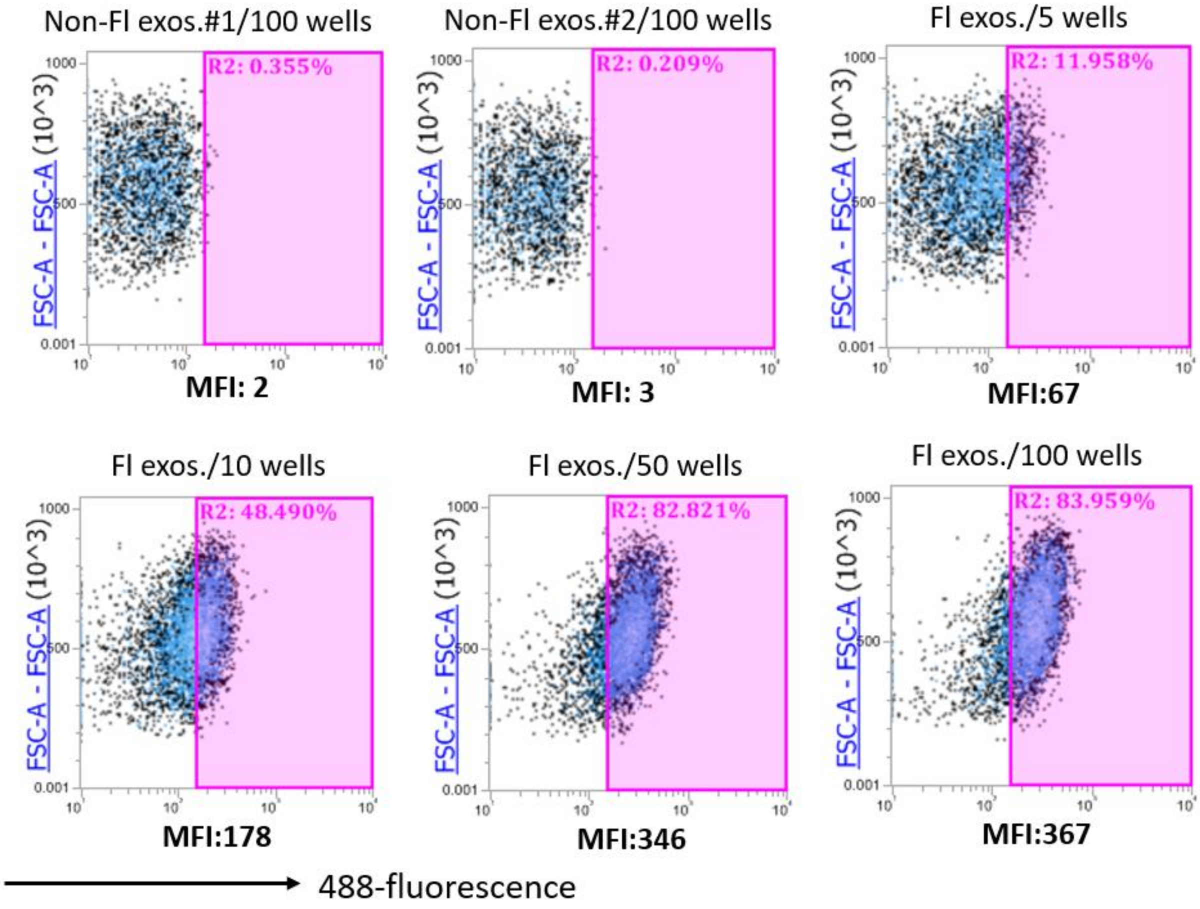

Figure S26: The representative one fluorescence (488) FACS density plots of HeLa cells incubated with different concentrations of A488-labeled exosomes (derived from 5-100 plate wells) for 12 h, with corresponding MFI value, along the 488-fluorescence channel, stated at the bottom of each plot. HeLa cells incubated with native nonfluorescent exosomes (non-fl exos.#1/100 wells) or glycoengineered exosomes missing DBCO-biotin treatment (non-fl exos.#2/100 wells) were utilized as the NCs. The 488-positive gate was set up with respect to both NCs, and the percentage of 488-positive cell population is given in each plot.

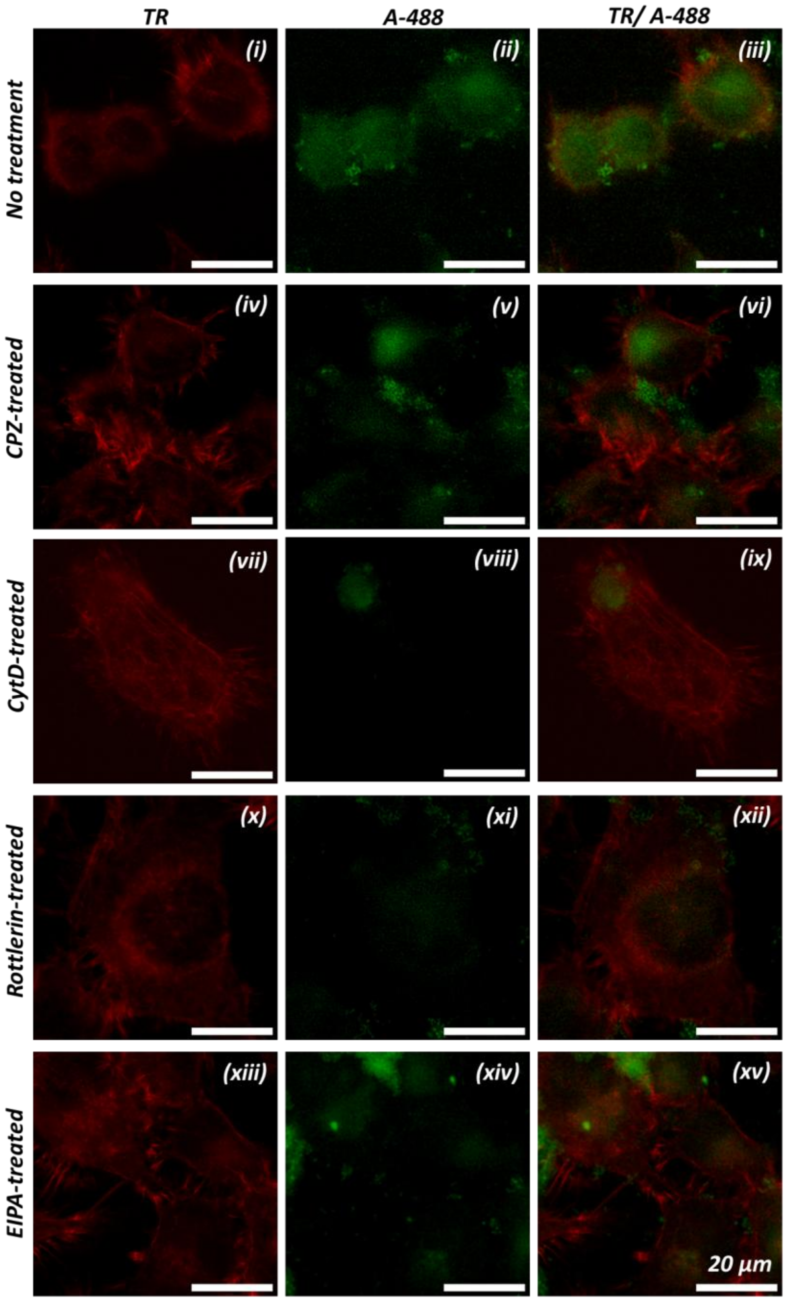

Figure S27. Fluorescent micrographs of HeLa cells treated with A488-labeled exosomes (10 h) without internalization inhibitors or with clathrin inhibitor CPZ and macropinocytosis inhibitors CytD, rottlerin, and EIPA, respectively. TR: actin network; A488: exosome; TR/A488: overlaps.

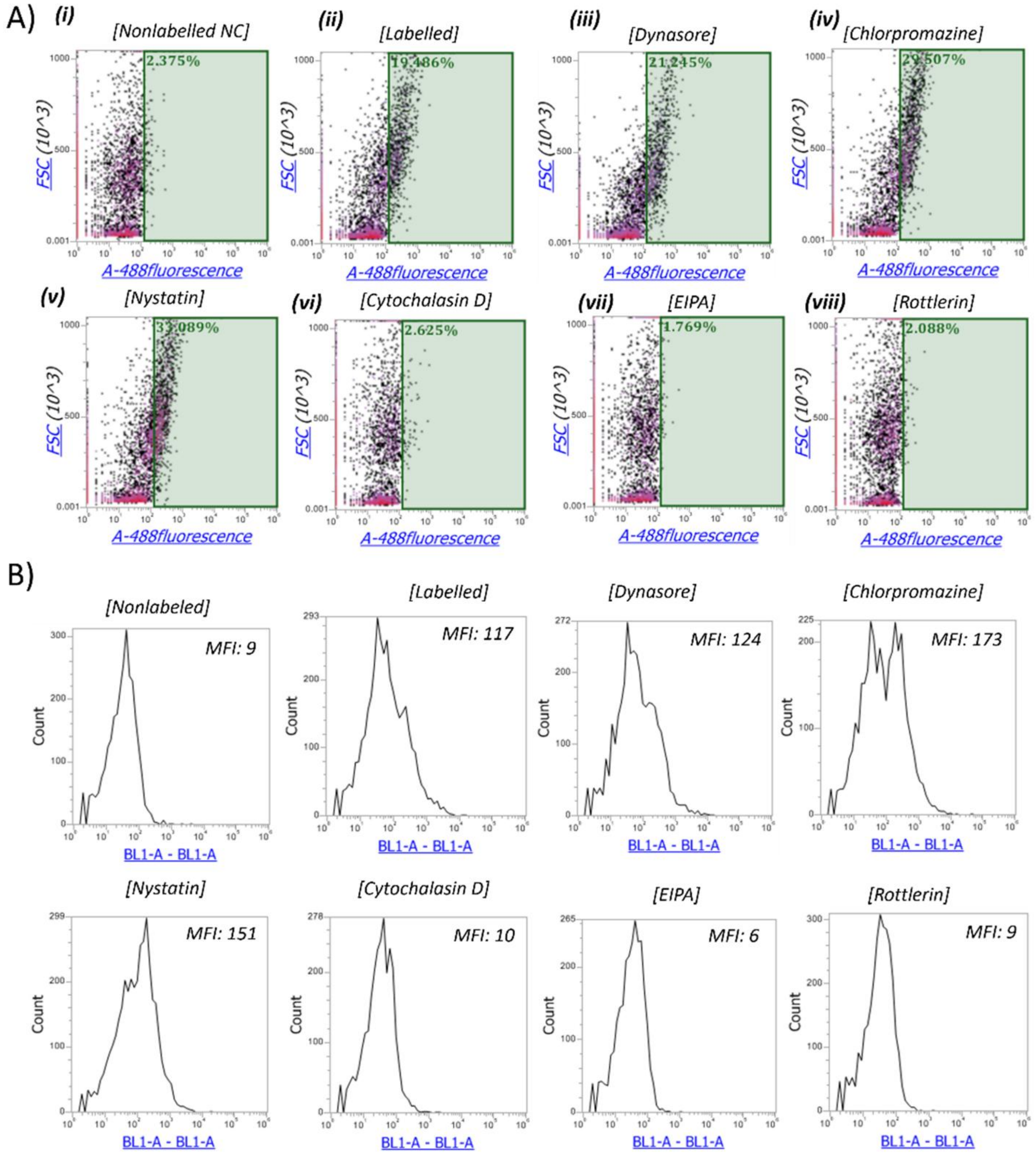

Figure S28. (A) Plots of gated one fluorescence (488) FACS density and (B) FACS histograms of HeLa cells incubated with A488-labeled exosomes (10 h) without internalization inhibitor or with clathrin inhibitors dynasore and CPZ, caveolae inhibitor nystatin, and macropinocytosis inhibitors CytD, rottlerin, and EIPA, respectively. TR: actin network; A488: exosome; TR/A488: overlaps.

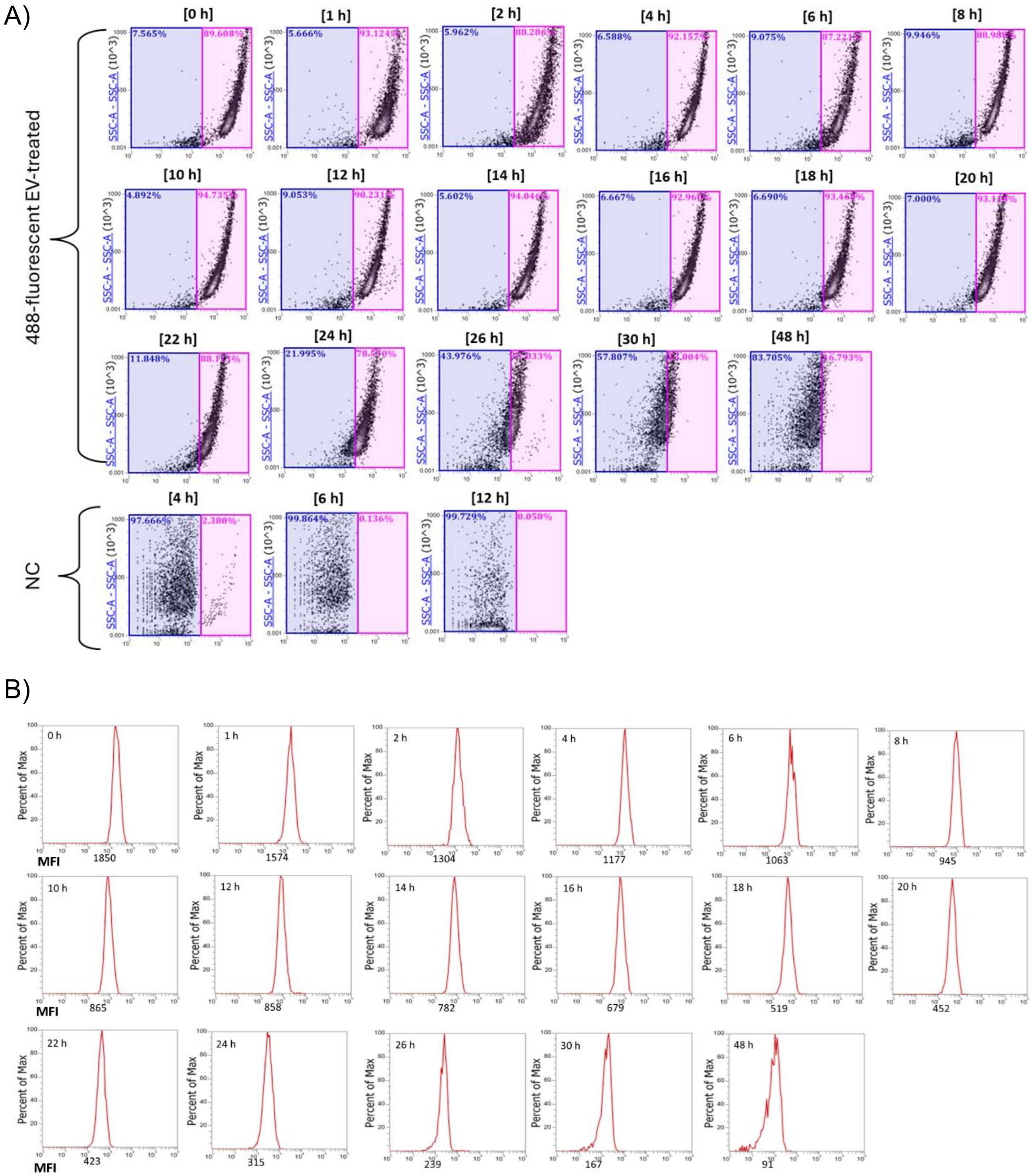

Figure S29: Fluorescence remained in Hela cells labeled with EGE-modified A488-tagged exosomes upon incubation in exosome-free low serum cell culture media (OptiMEM/0.5% FBS) for various lengths of time: (A) representative FACS density plots with the percentage of positive cells in the gated area given in the figure, and (B) representative FACS histograms showing the fluorescence intensity profiles and MFI values of cells at different time points. Cells incubated with native non-fluorescent exosomes at three different time points (4, 6, and 12 h) were used as the negative control (NC). The 488-positive gate (shown in pink) was set based on the NC, and the percentage of the 488-positive cell population is indicated in each plot.

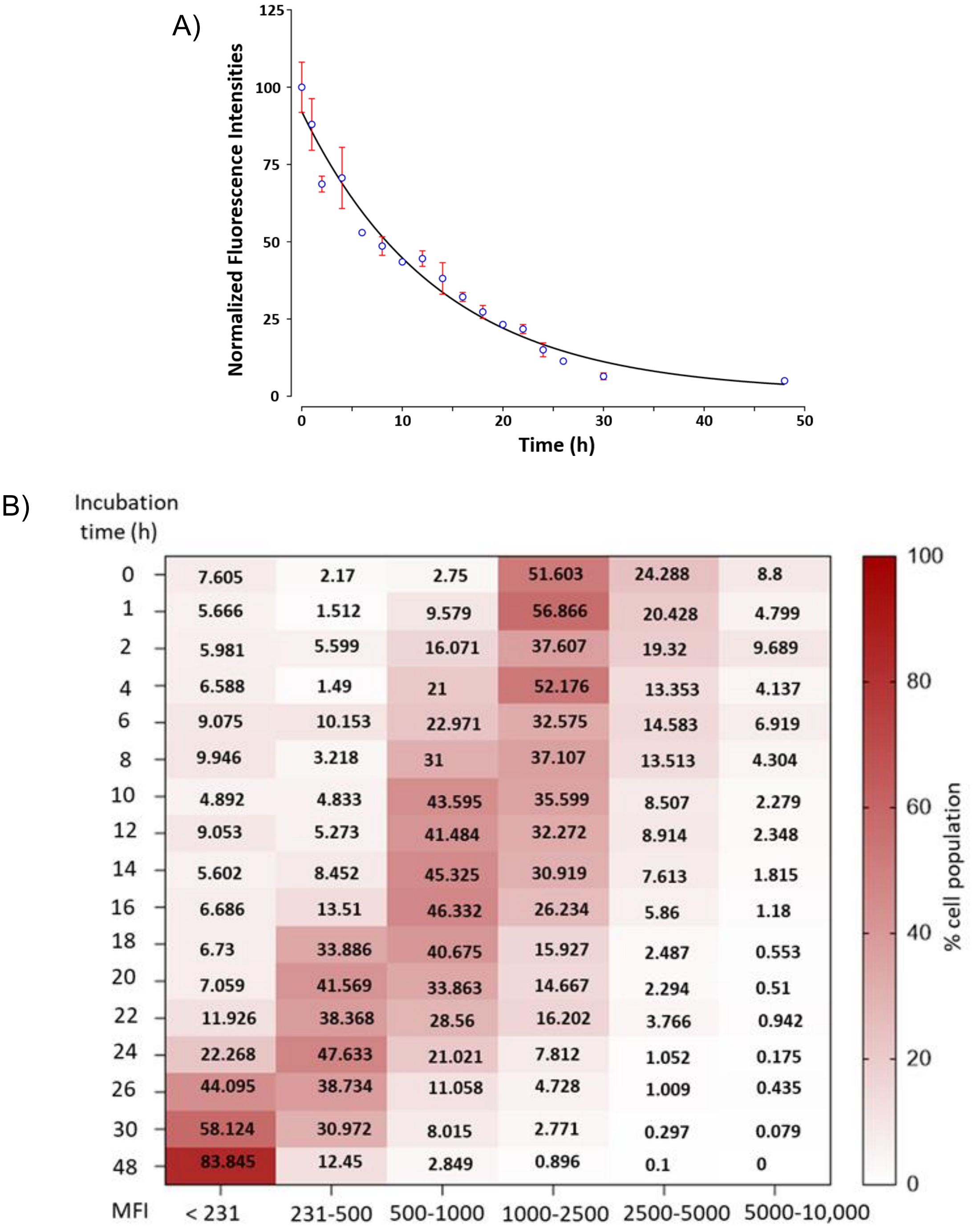

Figure S30: Fluorescence remained in Hela cells labeled with EGE-modified A488-tagged exosomes upon incubation in exosome-free low serum cell culture media (OptiMEM/0.5% FBS) for various lengths of time with the data from Figure S29 (A) in the form of a time-dependent decaying curve and (B) the heat map profile showing the cell density (as percentage of the total cell population on the right) and incubation time (Y-axis, lefts) against MFI values of cells (X-axis). The color in the map shows the cell density or population as indicated. Data are shown as the average of three parallel experiments ± SD.

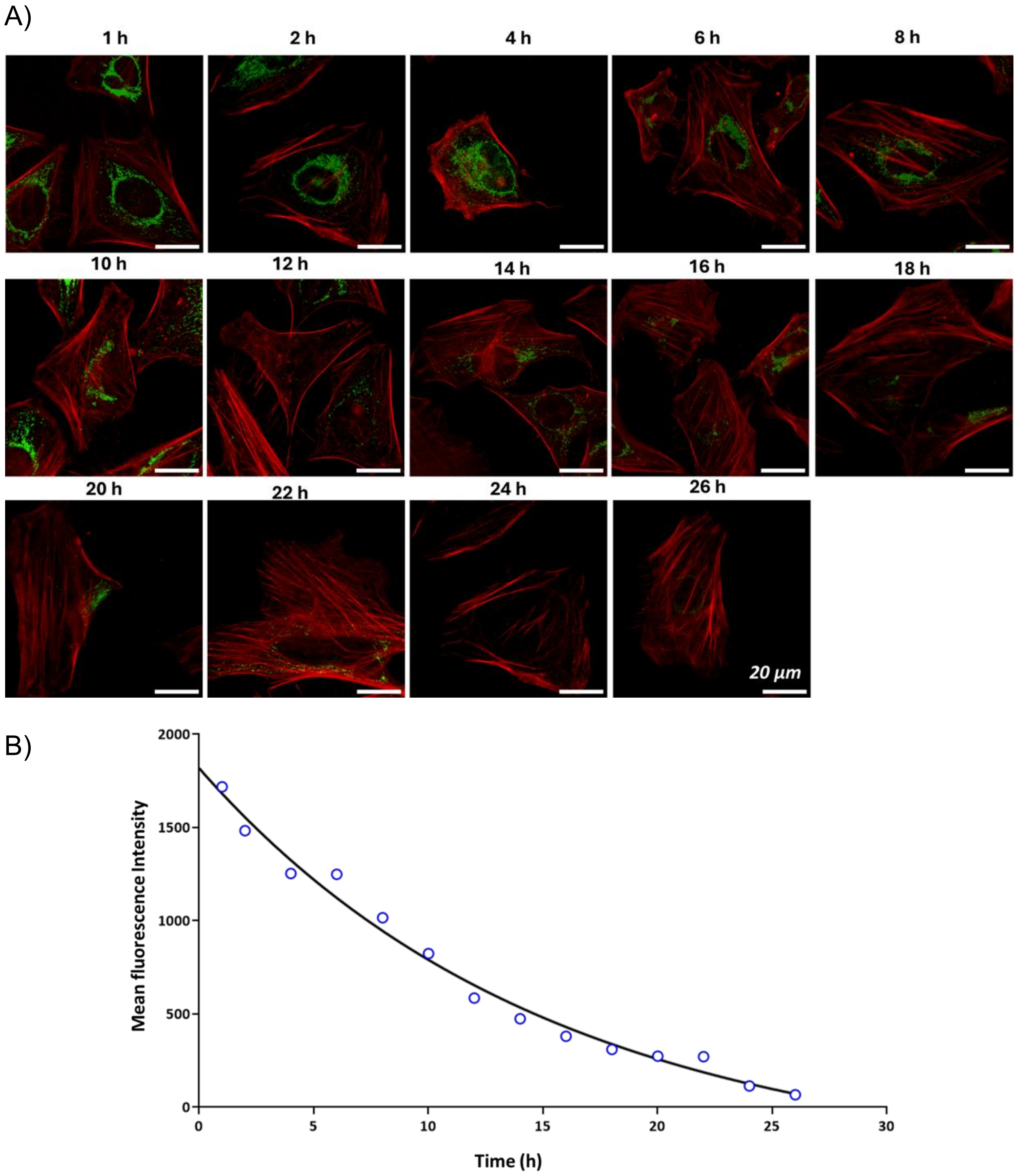

Figure S31: (A) Representative fluorescent images of HeLa cells labeled with EGE-modified A488-tagged exosomes upon incubation in exosome-free low serum cell culture media (OptiMEM/0.5% FBS) for various lengths of time, green (488-labeled exosome) and red (stain of cellular actins), and (B) the corresponding plot of the MFI values against time, with MFI values calculated from the fluorescent micrographs. Data are extracted from a single experiment.

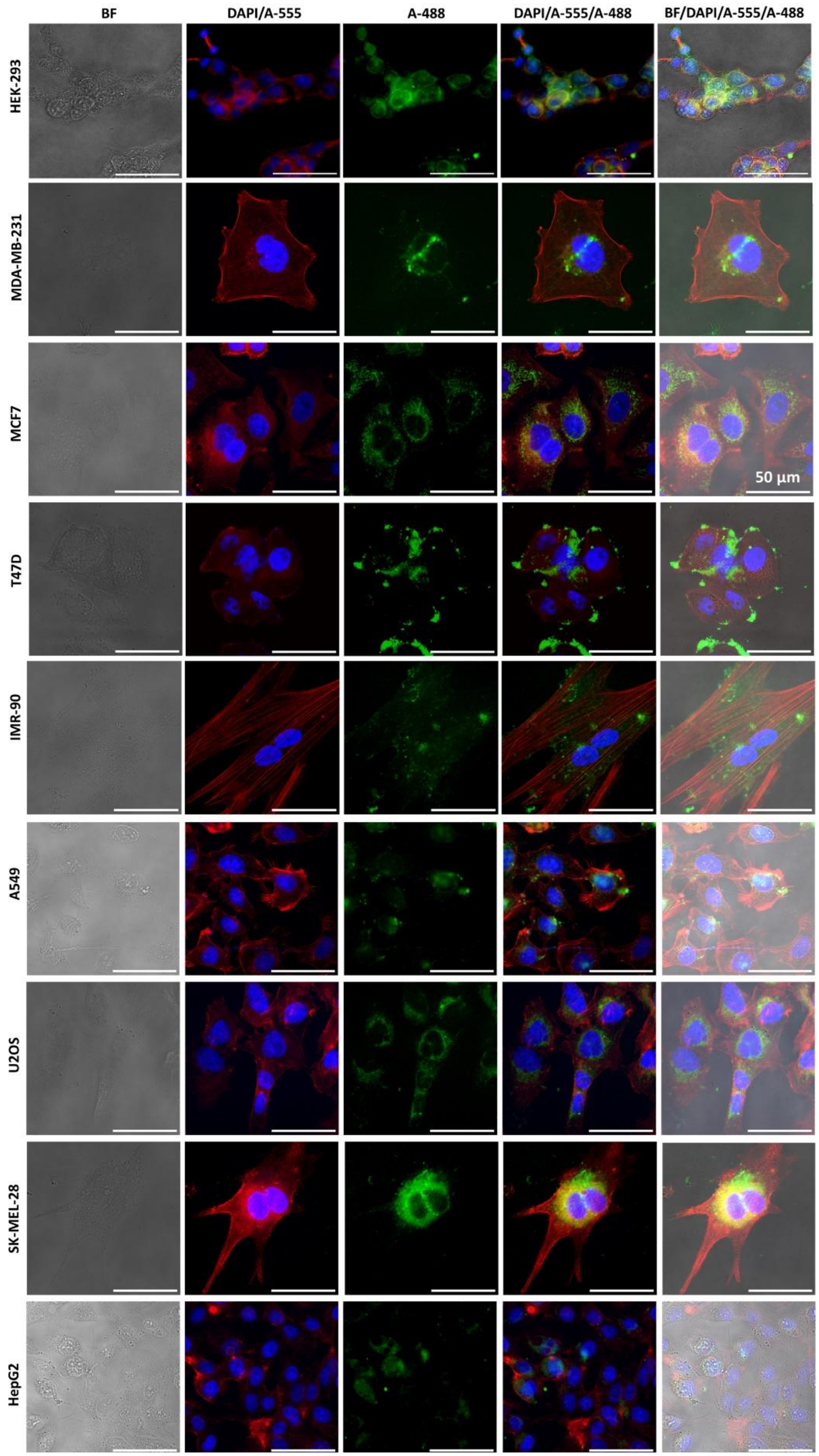

Figure S32: Fluorescent micrographs of various cells, as stated on the left side of each row, treated with 488-fluorescence labeled exosomes for 12 h. Green: exosome; Blue: nucleus; Red: cellular actin network.

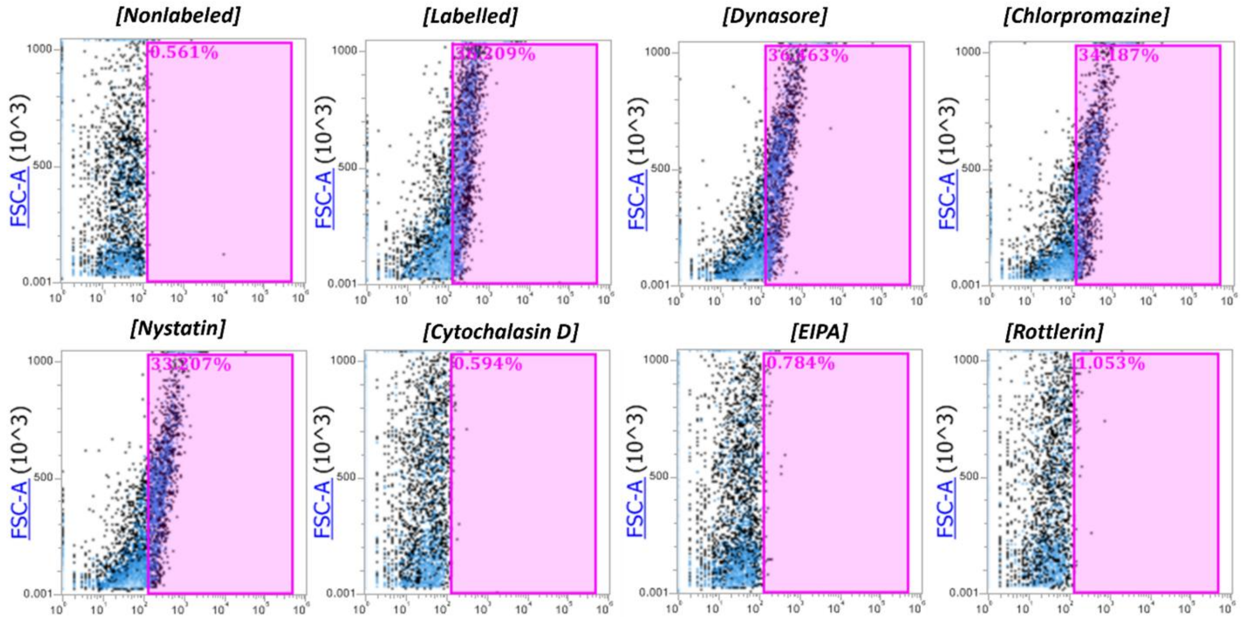

Figure S33: Gated one fluorescence (488) FACS density plots of HEK293 cells incubated with A488-labeled exosomes (12 h) without endocytosis inhibitor or with clathrin inhibitors dynasore and CPZ, caveolae inhibitor nystatin, and macropinocytosis inhibitors CytD, rottlerin, and EIPA, respectively. The 488-positive gate was set up with respect to the NC (Nonlabelled), and the percentage of 488-positive cell population is given in each plot.

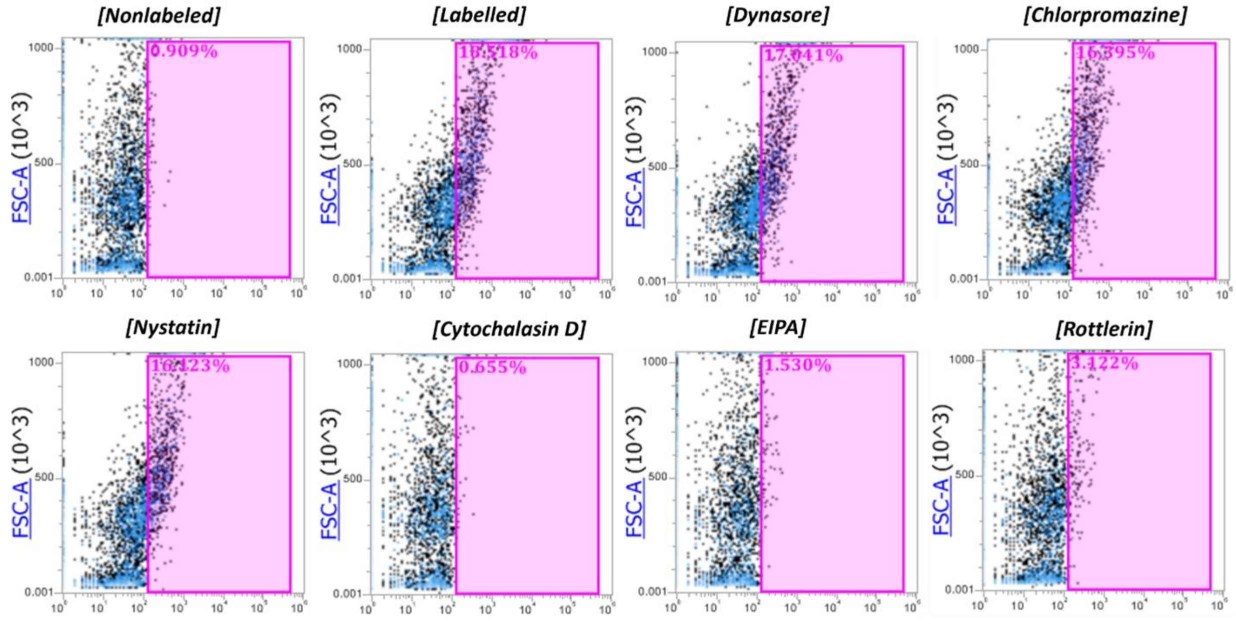

Figure S34: Gated one fluorescence (488) FACS density plots of MDA-MB-231 cells incubated with A488-labeled exosomes (12 h) without endocytosis inhibitor or with clathrin inhibitors dynasore and CPZ, caveolae inhibitor nystatin, and macropinocytosis inhibitors CytD, rottlerin, and EIPA, respectively. The 488-positive gate was set up with respect to the NC (Nonlabelled), and the percentage of 488-positive cell population is given in each plot. endocytosis inhibition.

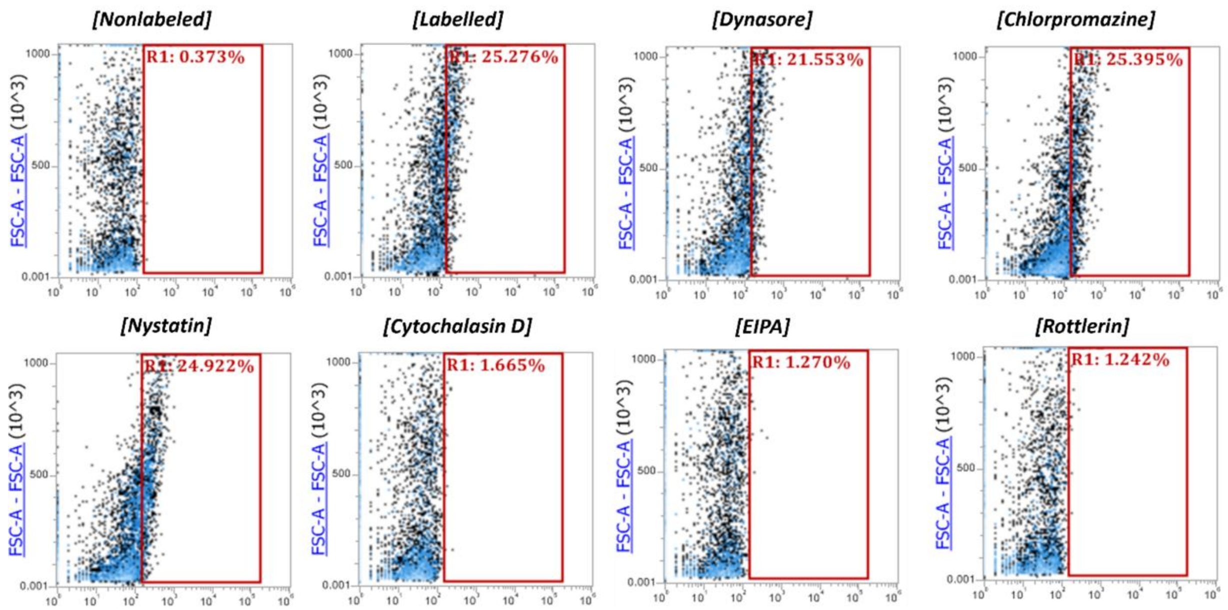

Figure S35: Gated one fluorescence (488) FACS density plots of MCF7 cells incubated with A488-labeled exosomes (12 h) without any endocytosis inhibitor or with clathrin inhibitors dynasore and CPZ, caveolae inhibitor nystatin, and macropinocytosis inhibitors CytD, rottlerin, and EIPA, respectively. The 488-positive gate was set up with respect to the NC (Nonlabelled), and the percentage of 488-positive cell population is given in each plot. endocytosis inhibition.

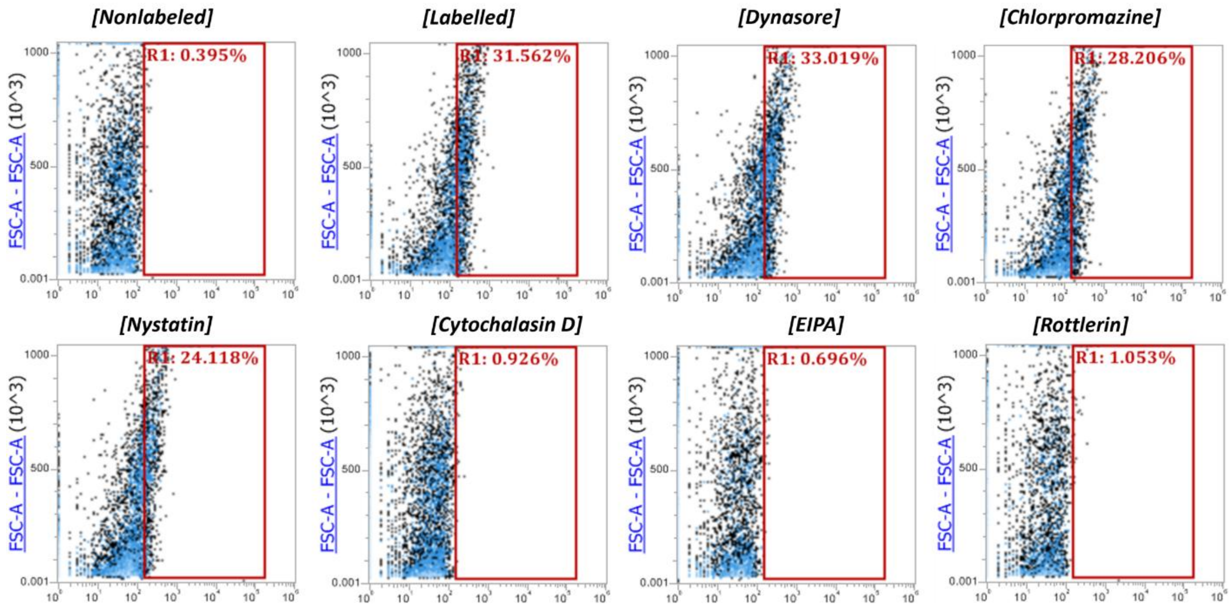

Figure S36: Gated one fluorescence (488) FACS density plots of T47D cells incubated with A488-labeled exosomes (12 h) without any endocytosis inhibitor or with clathrin inhibitors dynasore and CPZ, caveolae inhibitor nystatin, and macropinocytosis inhibitors CytD, rottlerin, and EIPA, respectively. The 488-positive gate was set up with respect to the NC (Nonlabelled), and the percentage of 488-positive cell population is given in each plot.

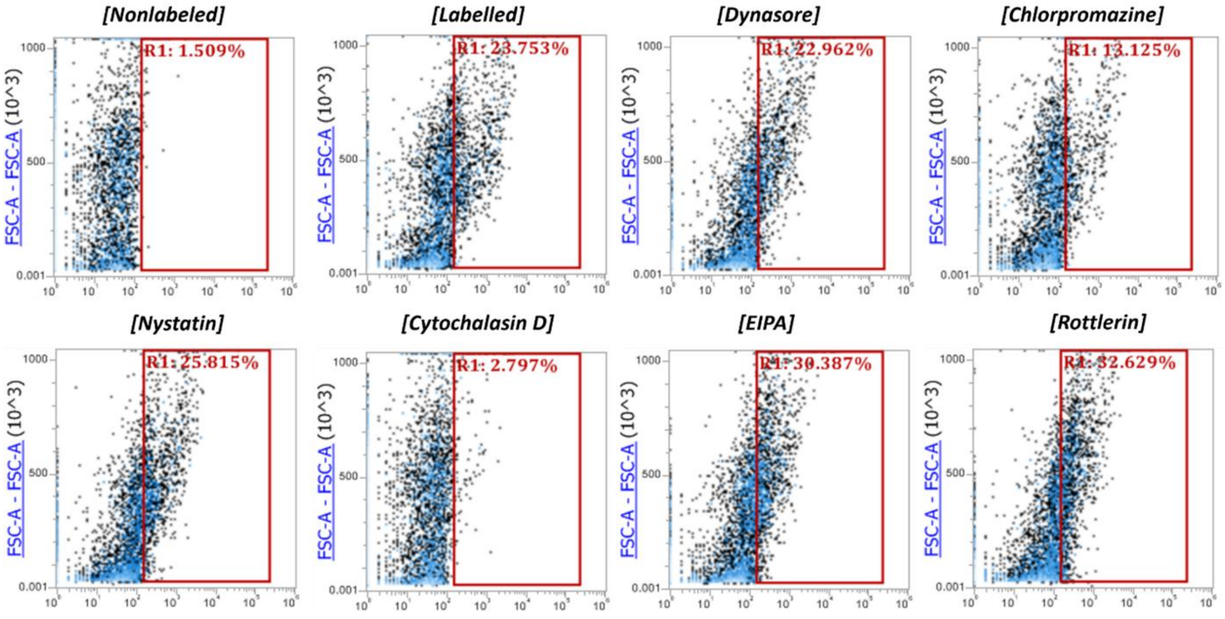

Figure S37: Gated one fluorescence (488) FACS density plots of LS174T cells incubated with A488-labeled exosomes (12 h) without any endocytosis inhibitor or with clathrin inhibitors dynasore and CPZ, caveolae inhibitor nystatin, and macropinocytosis inhibitors CytD, rottlerin, and EIPA, respectively. The 488-positive gate was set up with respect to the NC (Nonlabelled), and the percentage of 488-positive cell population is given in each plot.

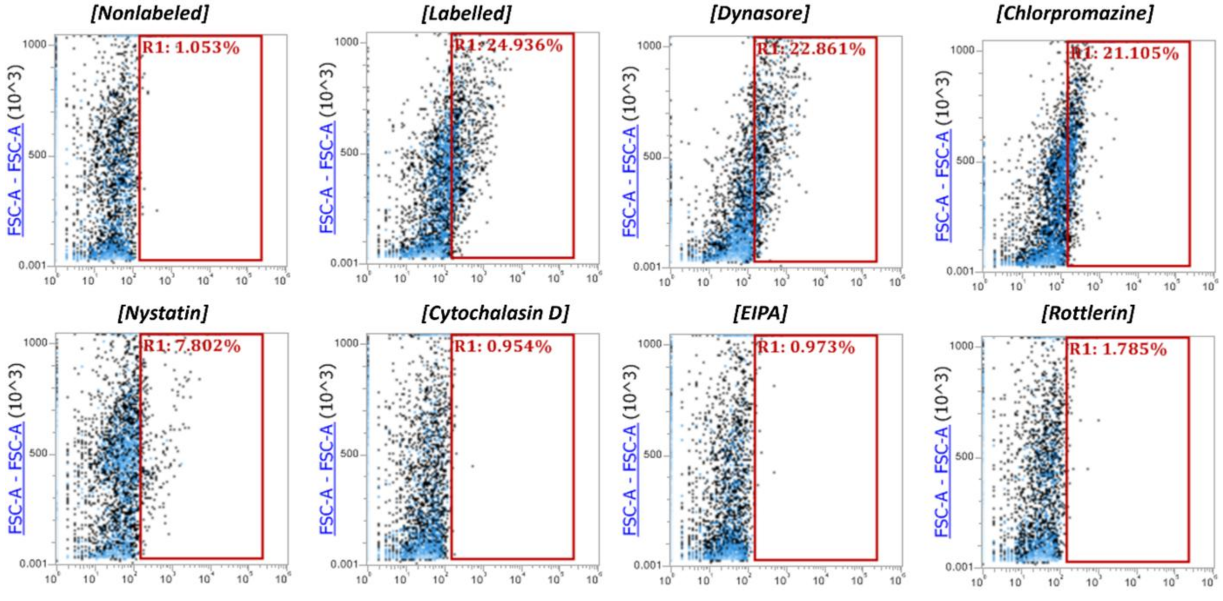

Figure S38: Gated one fluorescence (488) FACS density plots of A549 cells incubated with A488-labeled exosomes (12 h) without any endocytosis inhibitor or with clathrin inhibitors dynasore and CPZ, caveolae inhibitor nystatin, and macropinocytosis inhibitors CytD, rottlerin, and EIPA, respectively. The 488-positive gate was set up with respect to the NC (Nonlabelled), and the percentage of 488-positive cell population is given in each plot. endocytosis inhibition.

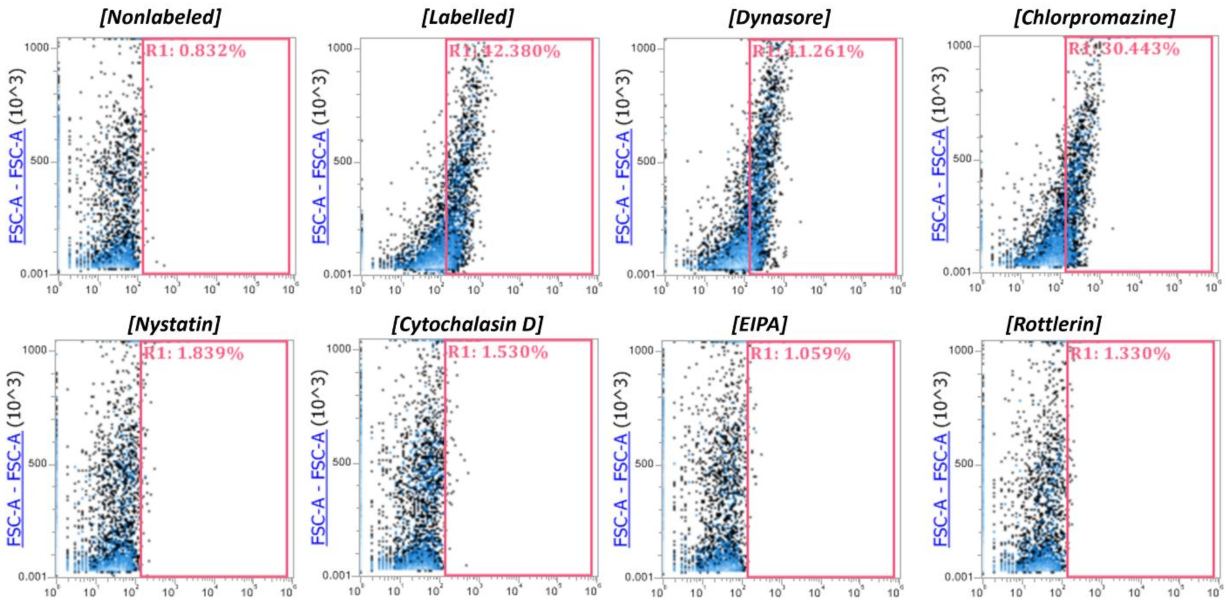

Figure S39: Gated one fluorescence (488) FACS density plots of U2OS cells incubated with A488-labeled exosomes (12 h) without any endocytosis inhibitor or with clathrin inhibitors dynasore and CPZ, caveolae inhibitor nystatin, and macropinocytosis inhibitors CytD, rottlerin, and EIPA, respectively. The 488-positive gate was set up with respect to the NC (Nonlabelled), and the percentage of 488-positive cell population is given in each plot.

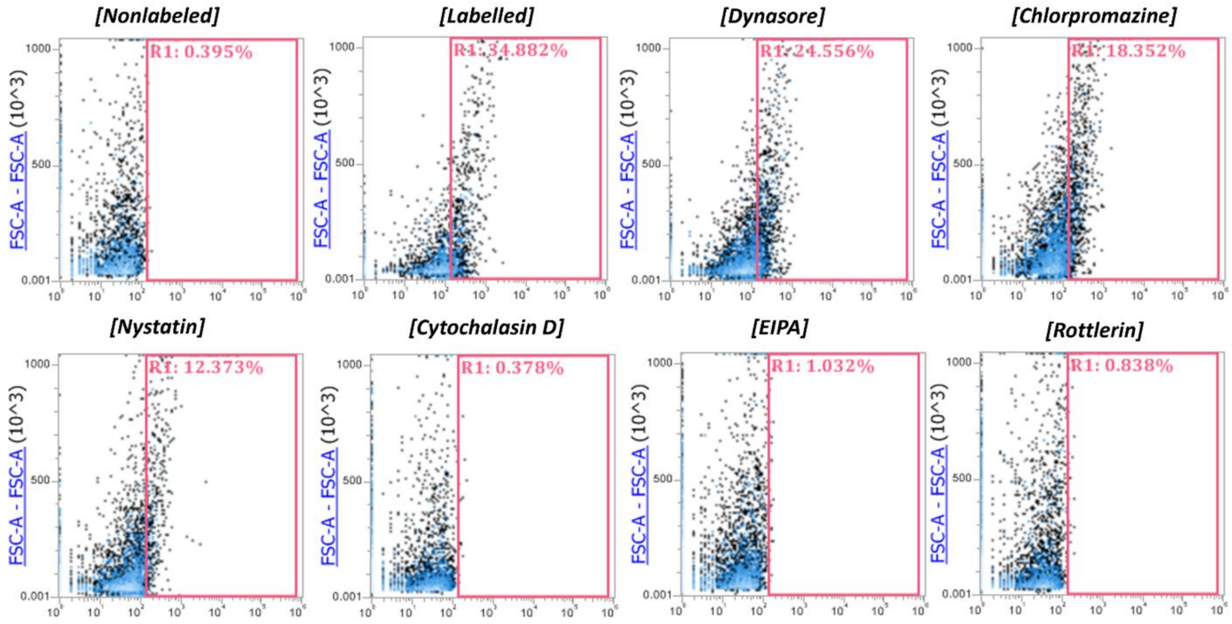

Figure S40: Gated one fluorescence (488) FACS density plots of SK-MEL-28 cells incubated with A488-labeled exosomes (12 h) without endocytosis inhibitor or with clathrin inhibitors dynasore and CPZ, caveolae inhibitor nystatin, and macropinocytosis inhibitors CytD, rottlerin, and EIPA, respectively. The 488-positive gate was set up with respect to the NC (Nonlabelled), and the percentage of 488-positive cell population is given in each plot.

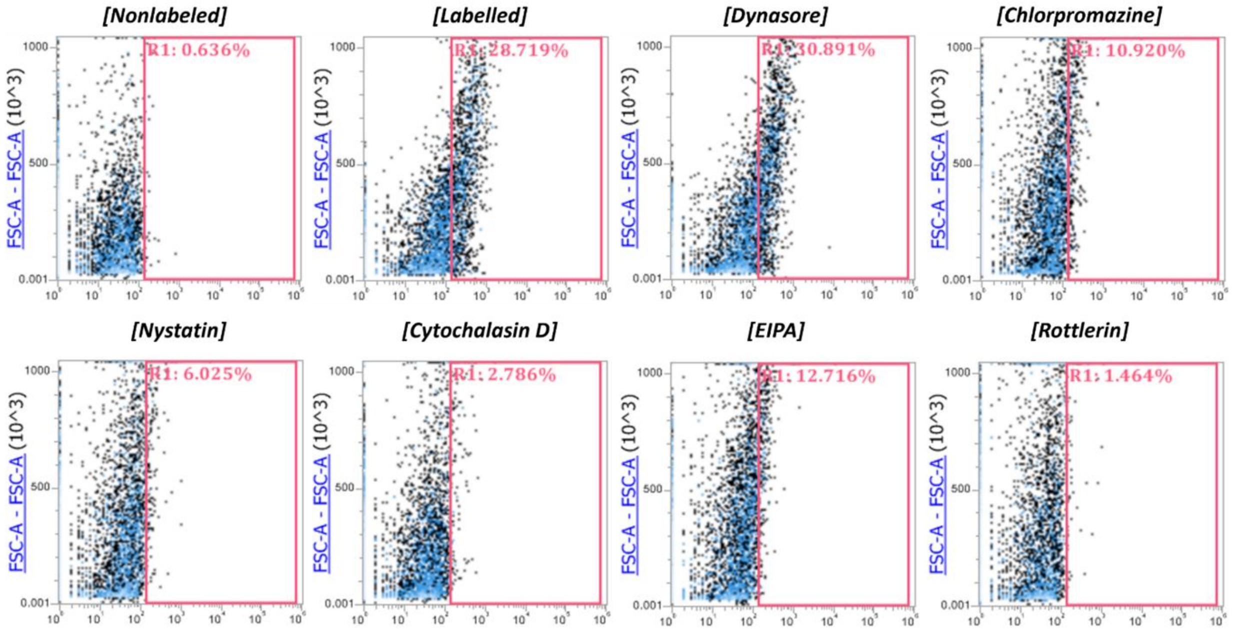

Figure S41: Gated one fluorescence (488) FACS density plots of HepG2 cells incubated with A488-labeled exosomes (12 h) without any endocytosis inhibitor or with clathrin inhibitors dynasore and CPZ, caveolae inhibitor nystatin, and macropinocytosis inhibitors CytD, rottlerin, and EIPA, respectively. The 488-positive gate was set up with respect to the NC (Nonlabelled), and the percentage of 488-positive cell population is given in each plot.

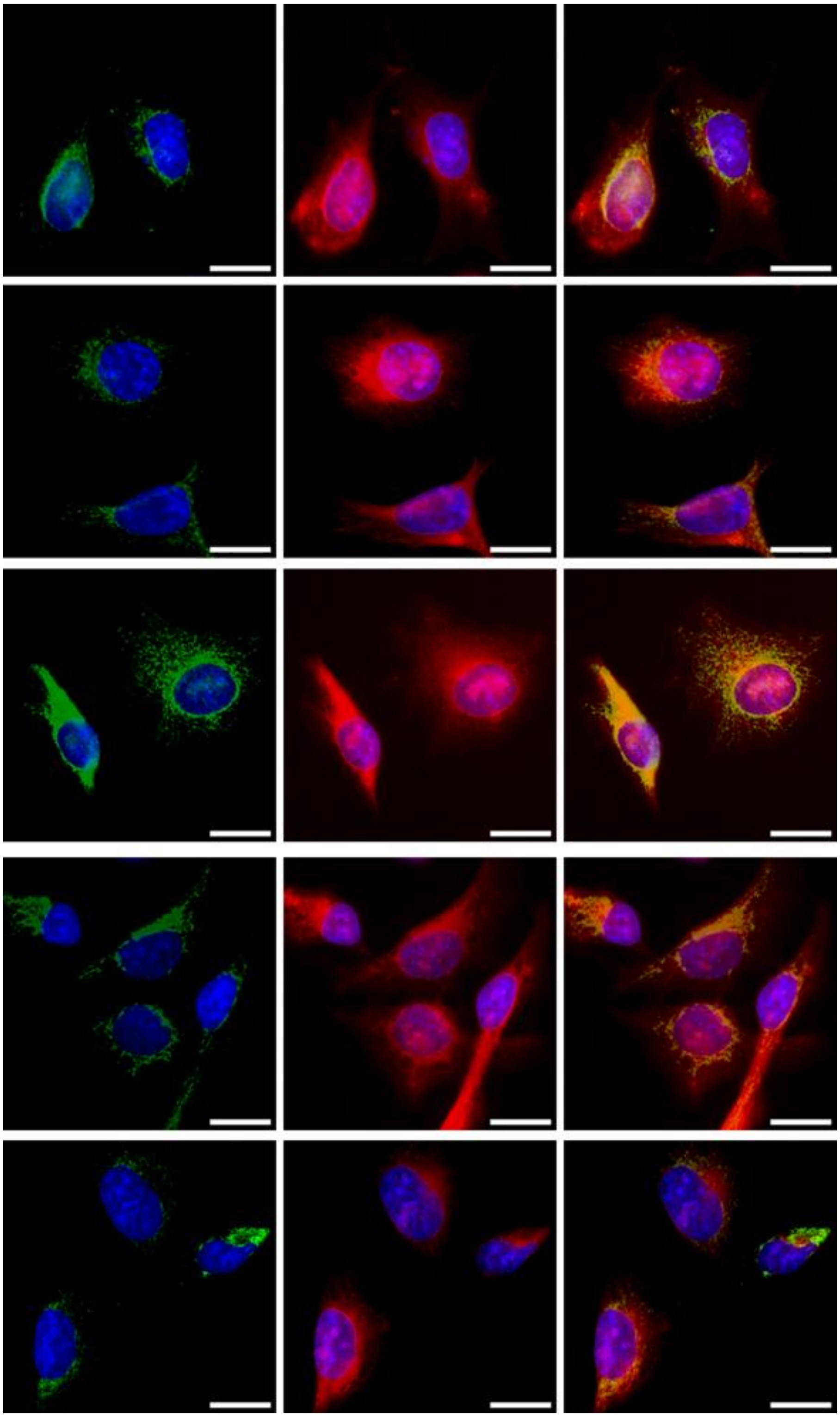

Figure S42: Fluorescent images of HeLa cells treated with EGE-modified A488-glycan (green) and TAMRA-protein (red) doubly labeled exosomes for 10 h, and the cells were then stained with DAPI for the cell nucleus (blue), fixed, and imaged using different fluorescence channels, as well as the merged fluorescence images from different channels. The bar: 20 μm. (from experiment #1)

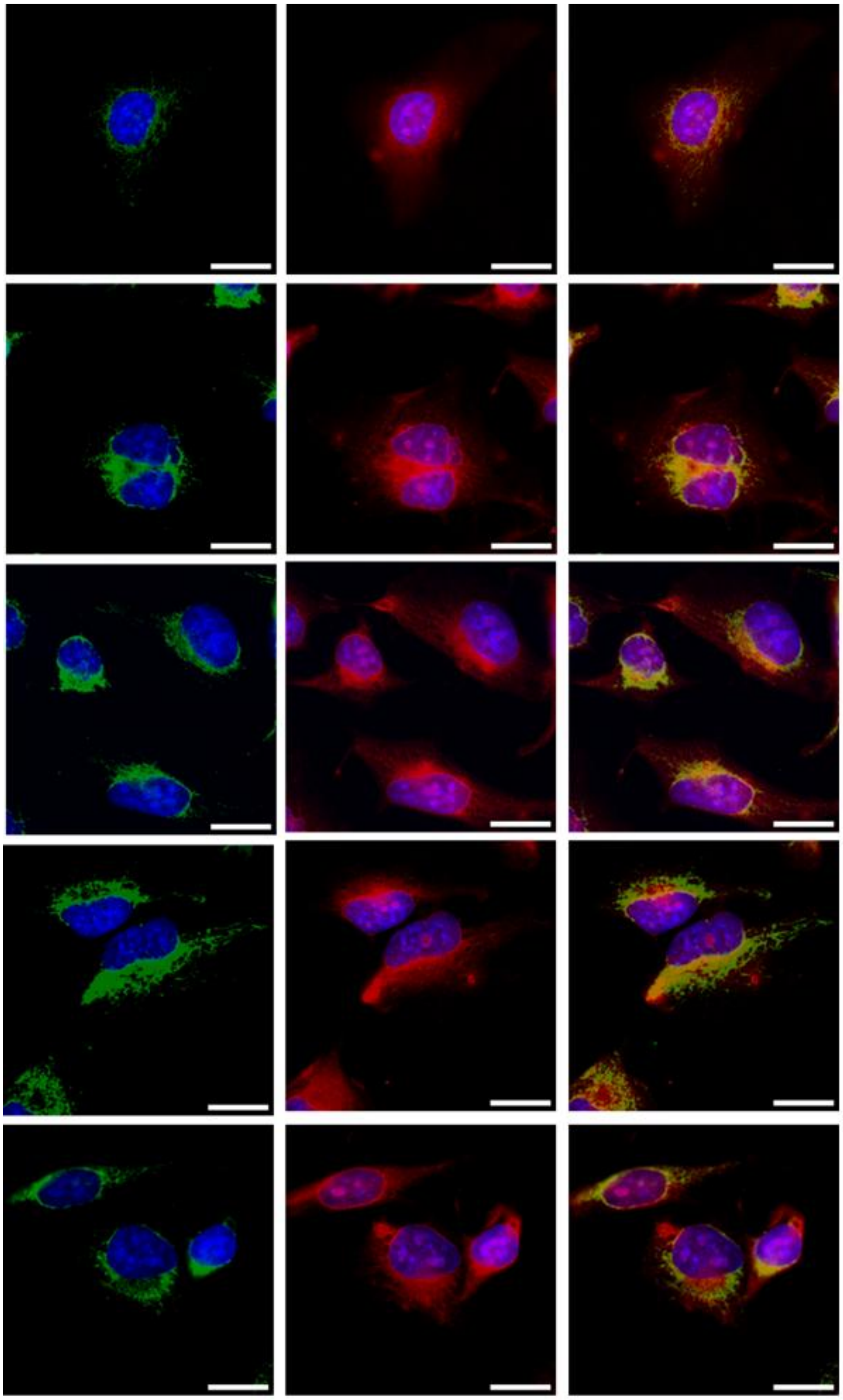

Figure S43: Fluorescent images of HeLa cells treated with EGE-modified A488-glycan (green) and TAMRA-protein (red) doubly labeled exosomes for 10 h, and the cells were then stained with DAPI for the cell nucleus (blue), fixed, and imaged using different fluorescence channels, as well as the merged fluorescence images from different channels. The bar: 20 μm. (from experiment #2)

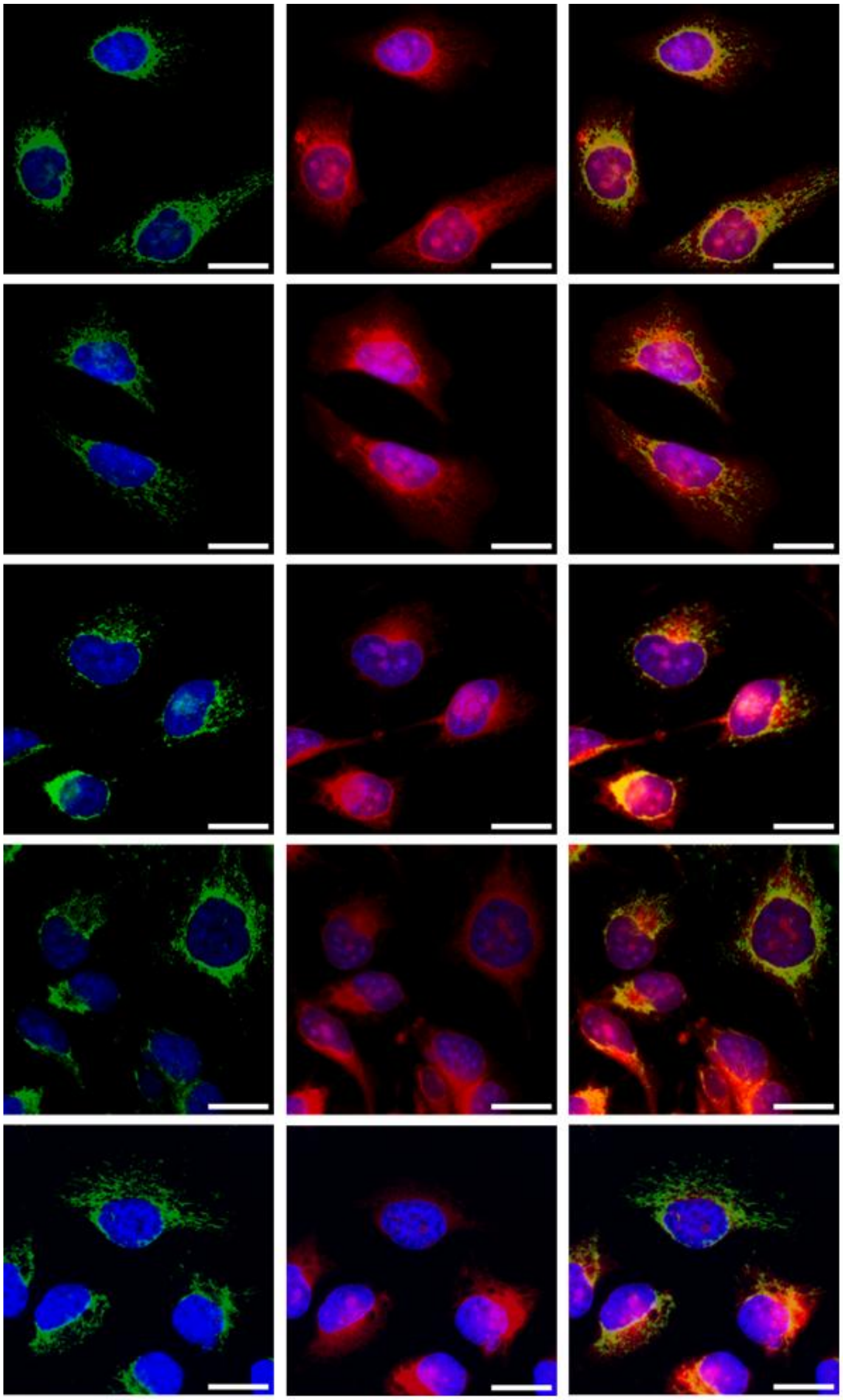

Figure S44: Fluorescence images of HeLa cells treated with EGE-modified A488-glycan (green) and TAMRA-protein (red) doubly labeled exosomes for 10 h, and the cells were then stained with DAPI for the cell nucleus (blue), fixed, and imaged using different fluorescence channels, as well as the merged fluorescence images from different channels. The bar: 20 μm. (from experiment #3)

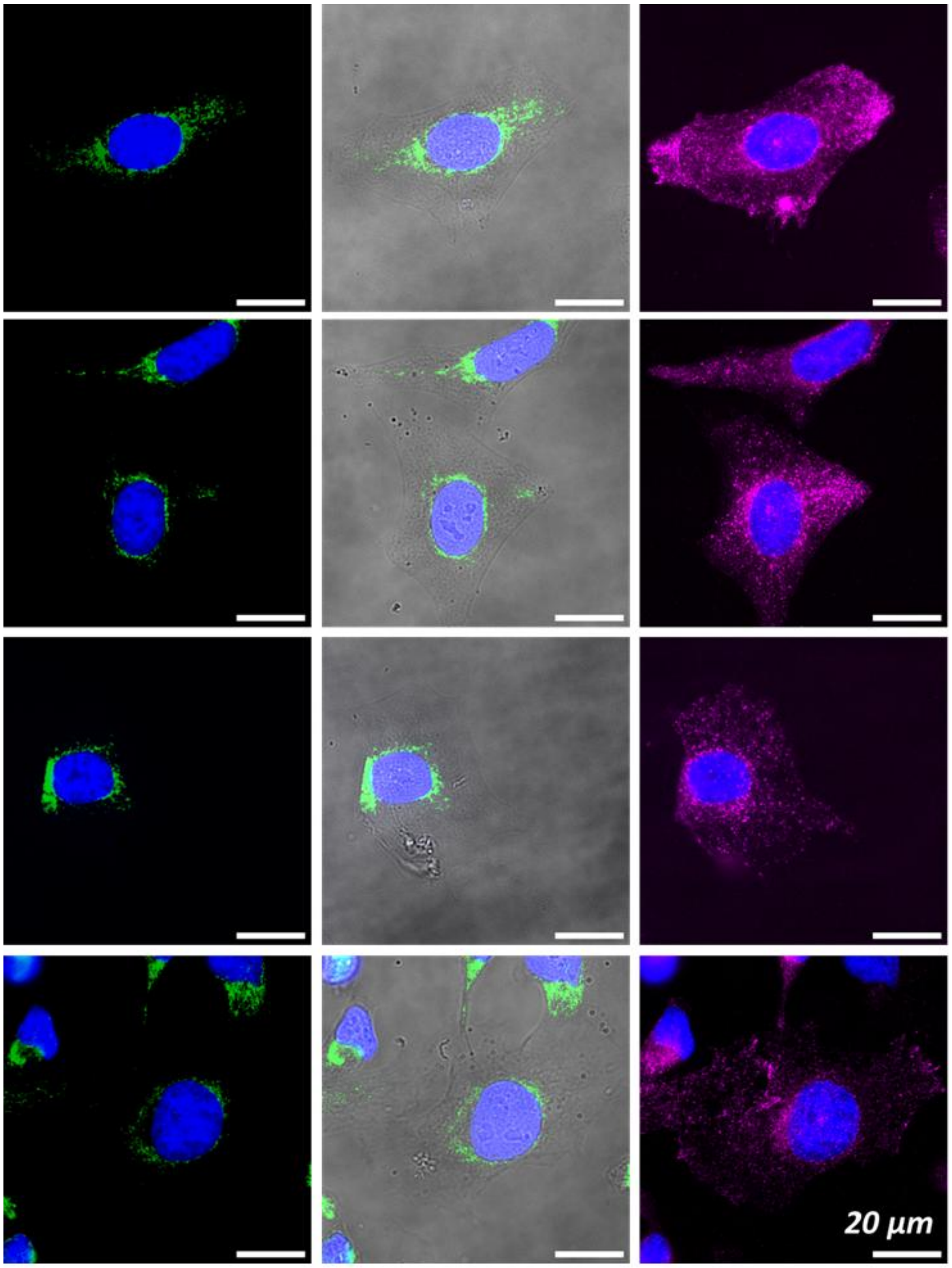

Figure S45: Fluorescence images of HeLa cells cultured with both EGE-modified A488-glycan (green)-labeled HS exosomes and A647-conjugated (magenta) transferrin for 10 h, which was followed by staining with DAPI for the cell nucleus (blue), fixation, and imaging using different fluorescence channels, as well as the merged fluorescence images from different channels. The bar: 20 μm.

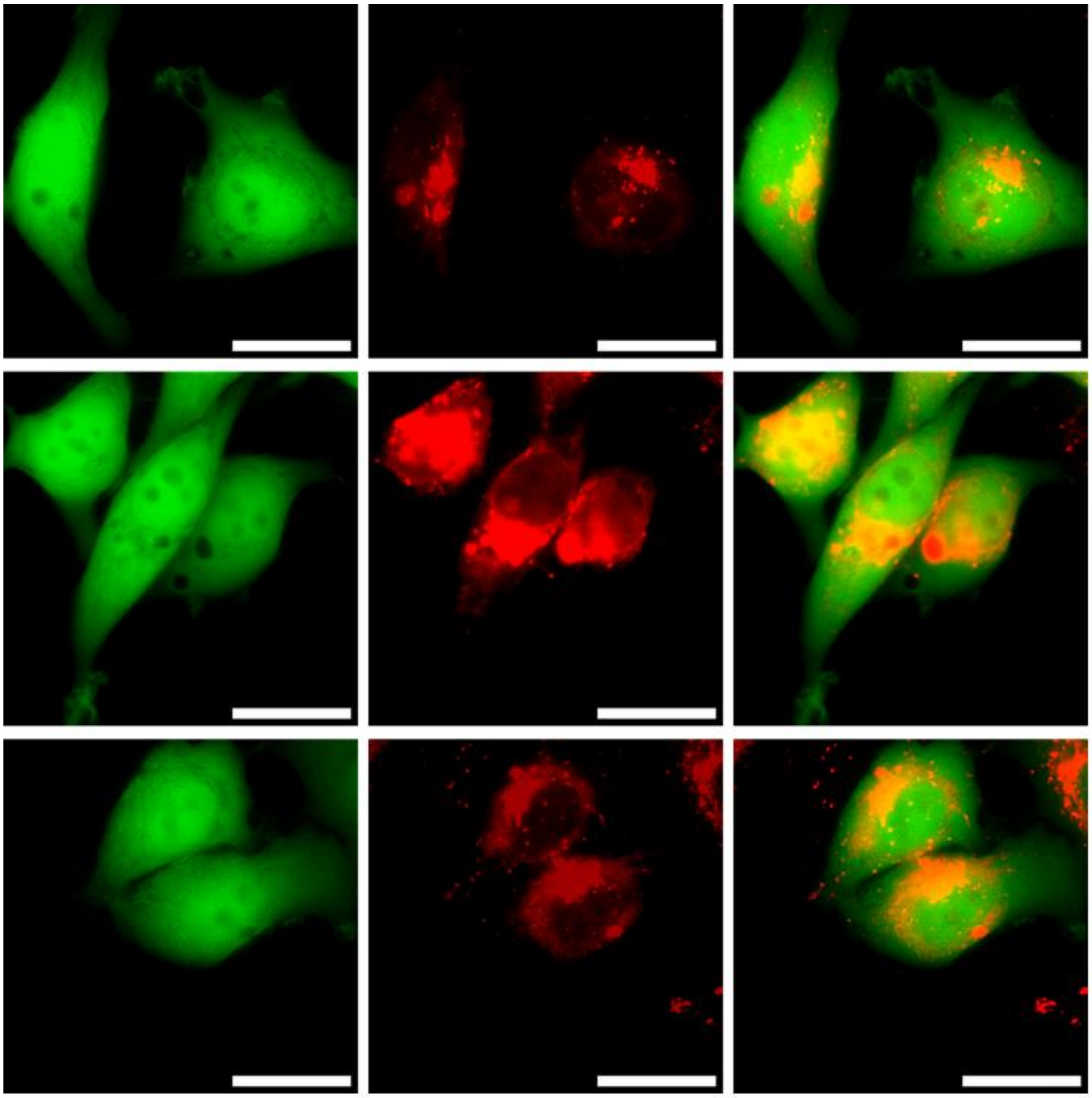

Figure S46: Fluorescent images of live HeLa cells having surface proteins labeled with A488 (green) after culturing with EGE-modified A555-glycan (red)-labeked HS exosomes for 10 h, as well as the merged fluorescence images. The cells were imaged using both fluorescence channels. The bar: 20 μm.

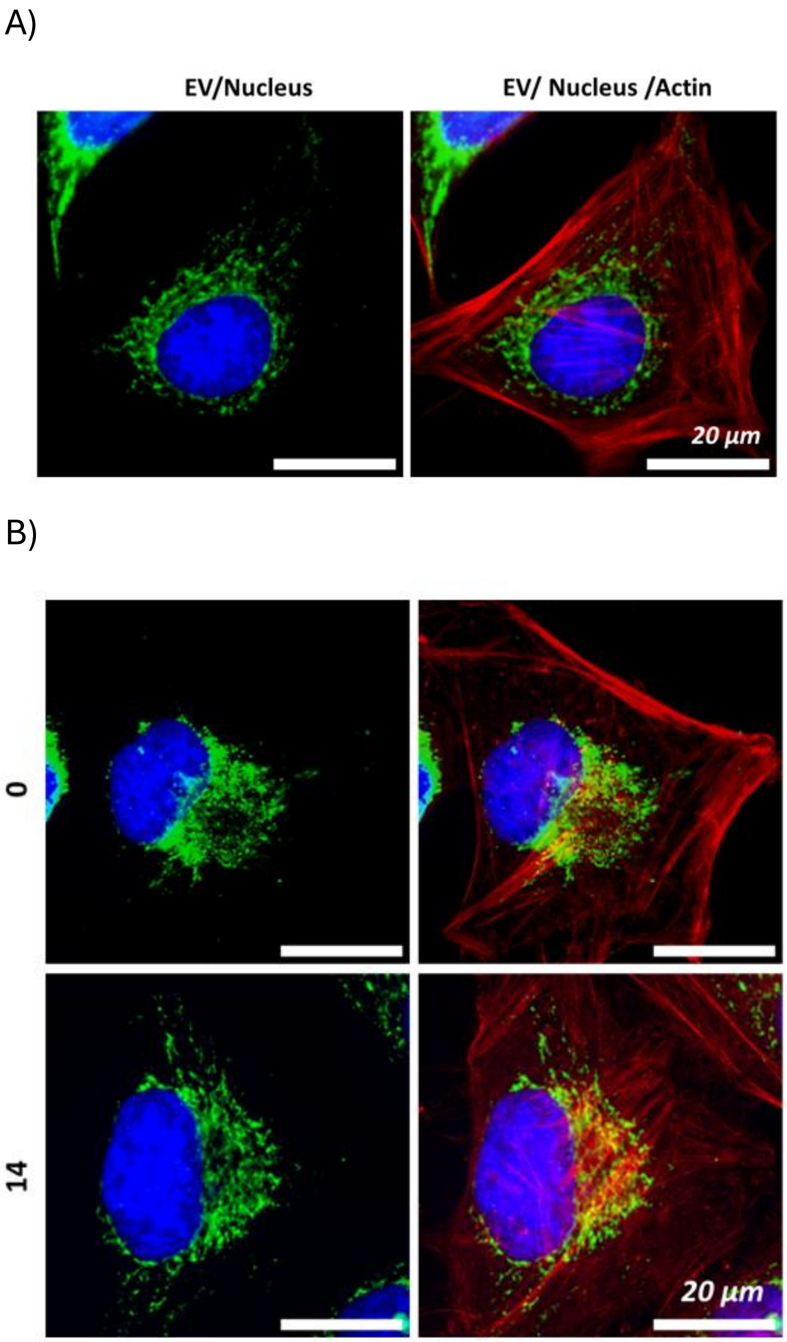

Figure S47: Fluorescence images of HeLa cells treated with (A) lyophilized EGE-modified A488-labeled HS exosomes, same as that in the experiment for Figure S17, and (B) EGE-modified A488-labeled HS exosomes stored in PBS at 37 ºC for 14 d, same as that in the experiment for Figure S18. The cells were incubated with exosomes for 10 h and then subjected to staining of actin and cell nucleus, washed, fixed, and imaged using the blue (nucleus), green (exosome), and red (actin) channels. The bar: 20 μm.

Table S1: Endocytosis inhibitors with concentration reported and used in this study*

Targeted pathway	Inhibitors	Concentration used in this study
Clathrin-mediated endocytosis	Chlorpromazine (CPZ)	50 µM
	Dynasore	100 µM
Caveolae-mediated endocytosis	Nystatin	50 µg/mL
Macropinocytosis	Cytochalasin D (CytD)	2.5 µM
	5-(*N*-ethyl-*N*-isopropyl) amiloride (EIPA)	30 µM
	Rottlerin	10 µM

* Reference: M. Itagaki, Y. Nasu, C. Sugiyama, I. Nakase, N. Kamei, *FASEB J.* **2023**, *37*, e22764.

Table S2. Proteins and antibodies used in this work

Entry	Target proteins or organelles	Antibody used	Vender: Catalogue No.
1	*For attachment of exosomes to coverslips or plate wells*		
1a	CD9 protein	CD9 Monoclonal antibody	Proteintech: 60232-1-Ig
1b	CD63 protein	CD63 Monoclonal antibody	Proteintech: 67605-1-Ig
1c	CD81 protein	CD81 Monoclonal antibody	Proteintech: 66866-1-Ig
2	*For on-plate detection of exosomes using the colorimetric method*		
2a	CD81 protein	Biotinylated anti-CD81 antibody	Ancell™: 302-030
2b	CD63 protein	Biotinylated anti-CD63 antibody	Ancell™: 215-030
2c	CD9 protein	Biotinylated anti-CD9 antibody	Ancell™: 156-030
3	*For detection of released exosomes by flow cytometry*		
3a	CD81 protein	ExoBrite™ 490/515 CD81 Flow Antibody	Biotium: P005
3b	CD63 protein	ExoBrite™ 490/515 CD63 Flow Antibody	Biotium: P004
3c	CD9 protein	ExoBrite™ 490/515 CD9 Flow Antibody	Biotium: P003
3d	Isotype control	ExoBrite™ 490/515 IgG1 Isotype Control	Biotium: P008
4	*For organelle colocalization study*		
4a	Endoplasmic reticulum (ER)	Alexa fluorophore® 647 Anti-Calreticulin [EPR3924]	Abcam: ab196159
4b	Golgi	Alexa fluorophore® 647 Anti-GM130 [EP892Y]	Abcam: ab195303
4c	Early endosome	Alexa fluorophore® 647 Anti-EEA1 [EPR4245]	Abcam: ab196186
4d	Late endosome	Alexa fluorophore® 647 Anti-RAB7 [EPR7588(B)]	Abcam: ab310133
4e	Lysosome	Alexa fluorophore® 647 Anti-LAMP1 [EPR21026]	Abcam: ab237303
4f	Actin network	CoraLite®594-Phalloidin (red)	Proteintech: PF00003

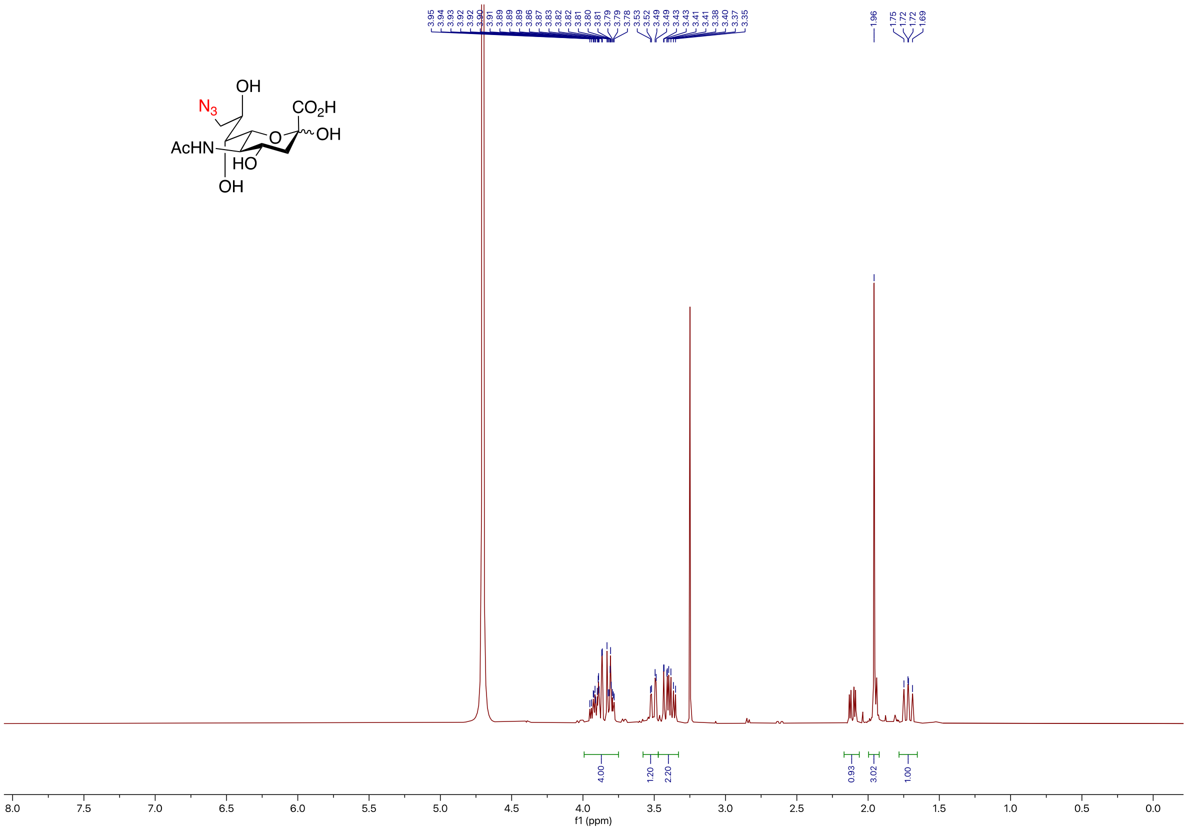

Figure S48: ^{1}H NMR (600 MHz, D_2O) spectrum of Neu5Ac9N_3.

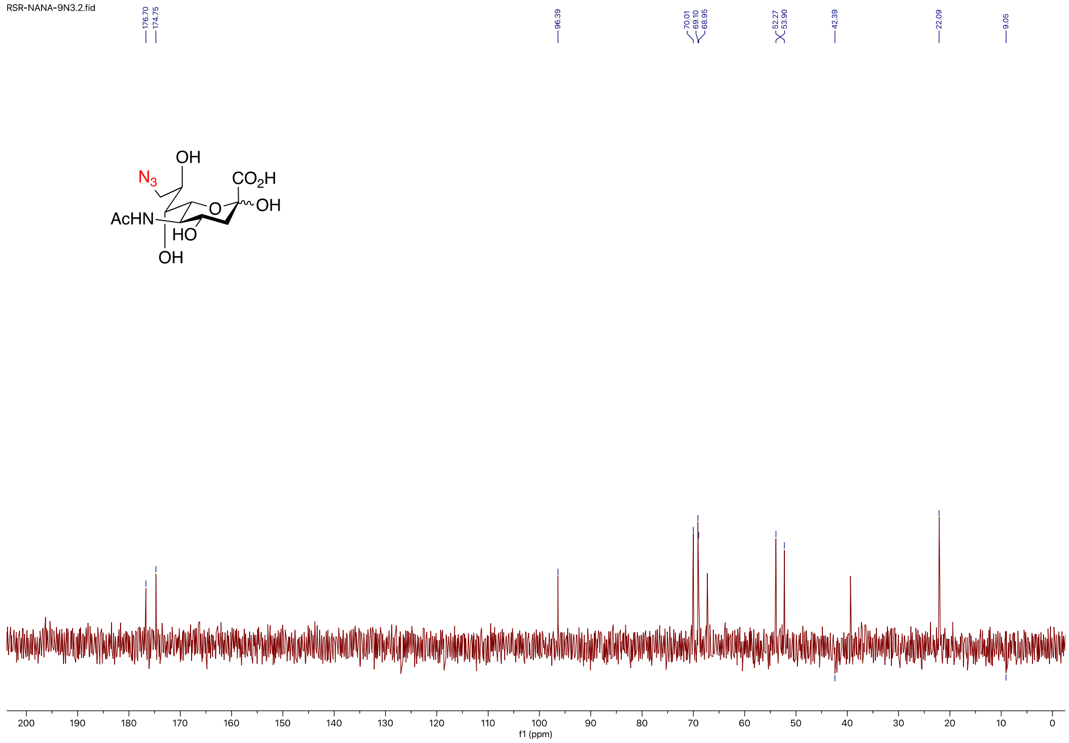

Figure S49: ^{13}C NMR (151 MHz, D_2O) spectrum of Neu5Ac9N_3.

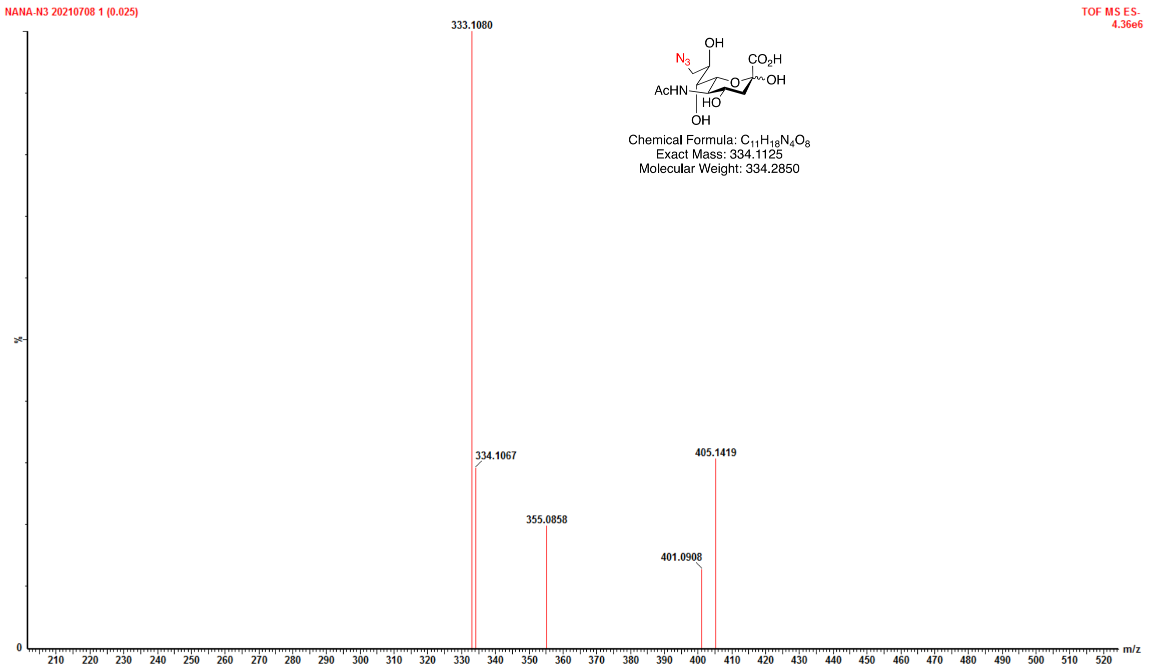

Figure S50: High resolution ESI(-)-TOF MS spectrum of Neu5Ac9N$_3$.

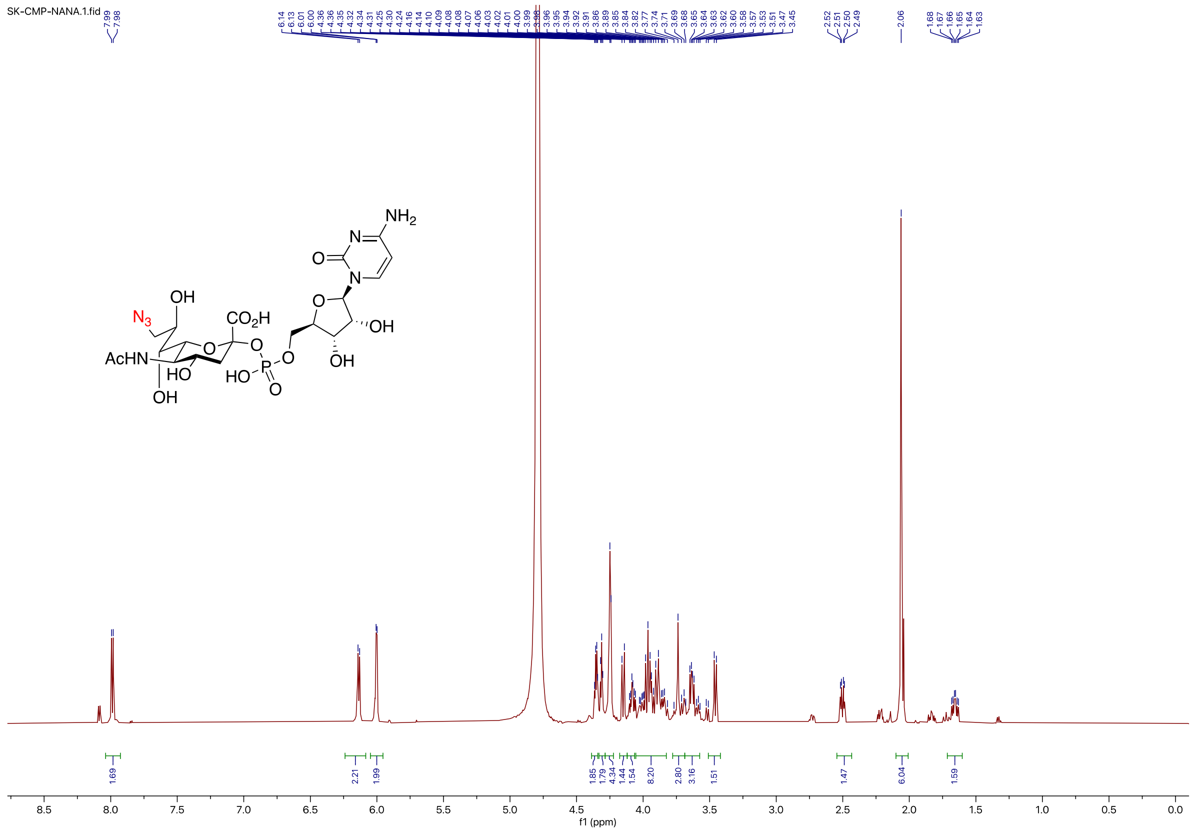

Figure S51: ^{1}H NMR (600 MHz, D_2O) spectrum of CMP-Neu5Ac9N$_3$.

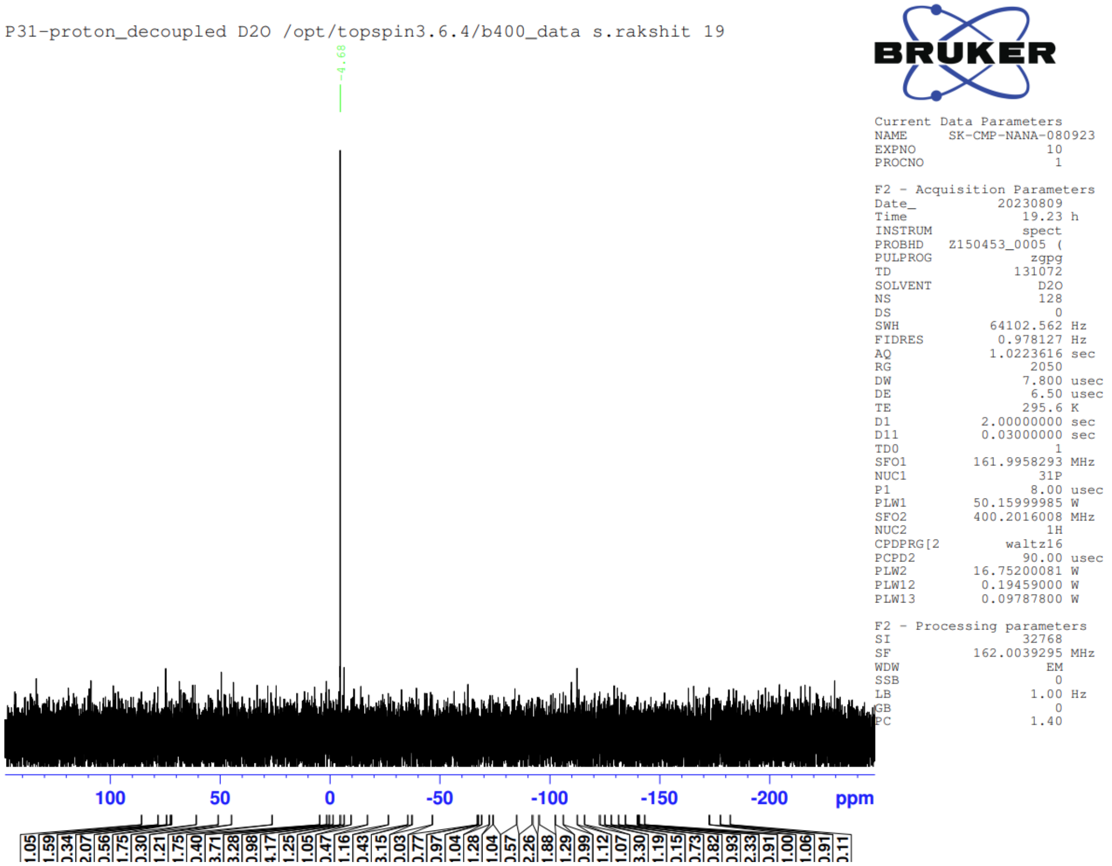

Figure S52: ^{31}P NMR (242 MHz, D_2O) spectrum of CMP-Neu5Ac9N_3.

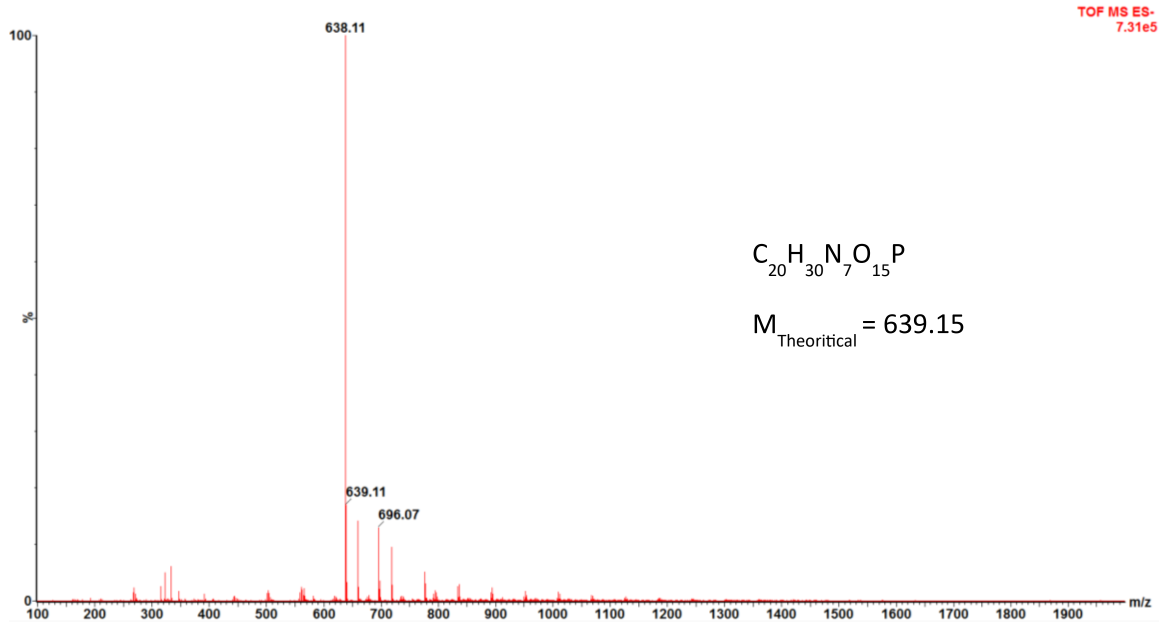

Figure S53: ESI(-)-TOF MS spectrum of CMP-Neu5Ac9N_3.

Table S3: Catalog numbers and providers of the commercial exosomes used in this study

Exosomes	Catalogue number	Provider
Human serum	HBM-PES-100/5	Hansa BioMed
HeLa	HBM-PEC-100/5	Hansa BioMed
SK-MEL-28	HBM-SK-MEL-28-100	Hansa BioMed
HEK293	HBM-HEK293-30/2	Hansa BioMed
MDA-231	EXOP-105A-1	System Bioscience
MDA-468	HBM-MDA-MB468-30/5	Hansa BioMed
SK-BR-3	HBM-SKBR3-30/2	Hansa BioMed

Certificate of Analysis

Lyophilized Exosomes from healthy donors of Human SERUM

HansaBioMed Life Sciences

LOT Specification

Lot Number	080724
Number of vials 100 ug	150
Number of vials 30 ug	
Particles in 30 ug vials	
Particles in 100 ug vials	1.70E+11
Expiration date	JUL/ 2027
Raw material batch used	HMN105835

Physical characteristic

Color	Colorless
Form	Dry
Odor	Not detectable
Solubility	Easily soluble
Consistency after reconstitution	Free of visible particles

Common EV marker expression

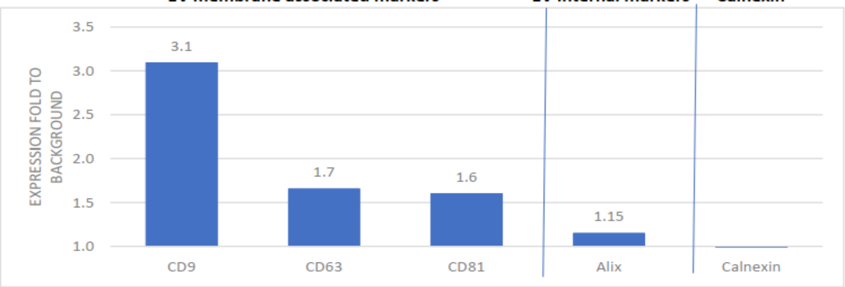

* fold to background smaller than 1 is considered negative

Particle size distribution

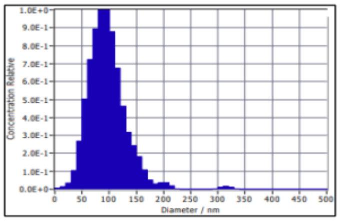

Concentration (part/ml)	1.70E+12
Stand Deviation (part/ml)	1.80E+11
Mean size (nm)	87.5
Stand Deviaton (nm)	3
Peak (nm)	89.3
Stand Deviaton (nm)	3.1

HansaBioMed Life Sciences Ltd

Mäealuse 2/1
12618, Tallinn, Estonia

Additional information:

**Application:**	Research use only, not for use in diagnostic procedures.
**Storage**	Lyophilized exosomes can be stored for 36 months at 4°C. Reconstituted exosomes can be stored at -20°C for up to one month or at -80°C for up to 6 months.
**Warranties**	HansaBioMed Life Sciences warrants that its products shall conform to the description of corresponding products as described in HBM's catalogue. This warranty is exclusive, and seller makes no other warranty, expressed or implied, including any implied warranty of merchantability or fitness for any particular purpose.

Approved by Sirin Korulu Koc

Signature

Released by Paolo Guazzi

Signature

Date: 16 07 2024
